# Efficacy and safety of therapeutic alpha-1-microglobulin RMC-035 in reducing kidney injury after cardiac surgery: a multicentre, randomised, double-blind, parallel group, phase 2a trial

**DOI:** 10.1016/j.eclinm.2024.102830

**Published:** 2024-09-16

**Authors:** Alexander Zarbock, Tobias E. Larsson, Nicolas Noiseux, C. David Mazer, Johannes Böhm, Maxime Laflamme, Klaus Matschke, Jan Burkert, Benoit de Varennes, Andrej Myjavec, Andreas Böning, Jay L. Koyner, Dan Engelman, Michael Reusch, Matthias Thielmann, AKITA investigators, Belén Adrio Nazar, Johannes Böhm, Andreas Böning, Craig Brown, Jan Burkert, Benoit de Varennes, Cara East, Dan Engelman, Antonino Ginel Iglesias, Sven Helms, Jay L. Koyner, David Kress, Maxime Laflamme, Andre Lamy, Tobias E. Larsson, Klaus Matschke, C David Mazer, Guillermo Muniz Albaiceta, Ignacio Munoz Carvajal, Andrej Myjavec, Nicolas Noiseux, Saturo Osaki, Michael Reusch, Guillermo Reyes Copa, Claudio Ronco, Vincent Scavo, Ryan Shelstad, Madhav Swaminathan, Gabor Szabo, Nicholas Teman, Matthias Thielmann, Jan Vojacek, Thorsten Wahlers, Alexander Zarbock

**Affiliations:** a Department of Anaesthesiology, Intensive Care and Pain Medicine, University Hospital Münster, Münster, Germany; b Guard Therapeutics International AB, Stockholm, Sweden; c Centre de Recherche du Centre Hospitalier de l'Université de Montréal, Montréal, Québec, Canada; d St. Michael's Hospital, University of Toronto, Toronto, Canada; e Department of Cardiovascular Surgery, Institute Insure, German Heart Center Munich, Technical University of Munich, Munich, Germany; f Institut Universitaire de Cardiologie et de Pneumologie de Québec, Québec, Canada; g Klinik für Herzchirurgie, Herzzentrum Dresden Universitätsklinik, Dresden, Germany; h Fakultni Nemocnice v Motole, Prague, Czech Republic; i McGill University Health Centre - Royal Victoria Hospital, Montréal, Canada; j Department of Cardiac Surgery, Charles University, Faculty of Medicine and University Hospital in Hradec Kralove, Hradec Kralove, Czech Republic; k Department of Cardiovascular Surgery, Justus-Liebig-University Giessen, Germany; l Section of Nephrology, University of Chicago, Chicago, IL, USA; m Division of Cardiac Surgery, Baystate Medical Center, Springfield, MA, USA; n Klinik für Thorax- und Kardiovaskuläre Chirurgie, Westdeutsches Herz- und Gefäßzentrum Essen, Universität Duisburg-Essen, Germany

**Keywords:** Acute kidney injury, Cardiac surgery, Cardiopulmonary bypass, Reperfusion injury, Alpha-1-microglobulin

## Abstract

**Background:**

Cardiac surgery invariably triggers acute kidney stress causing adverse renal outcomes. The AKITA study evaluated the efficacy and safety of RMC-035, a novel analogue of alpha-1-microglobulin, for reducing cardiac surgery-associated kidney injury.

**Methods:**

In this randomised double-blind placebo-controlled phase 2a study, we randomly assigned (1:1) adult hospitalised patients undergoing open-chest cardiac surgery at high risk for acute kidney injury (AKI) at 21 sites in North America and Europe to receive either RMC-035 (1.3 or 0.65 mg/kg) or placebo (1:1) for 2 days (5 intravenous infusions), stratified by region and renal function. Eligible patients had at least one pre-defined AKI risk factor. Patients with severe renal impairment (estimated glomerular filtration rate [eGFR] <30 mL/min/1.73 m^2^) were excluded. The co-primary efficacy and safety endpoints were AKI (Kidney Disease: Improving Global Outcomes definition) within 72 h after surgery and nature, frequency, and severity of treatment-emergent adverse events (TEAEs). Secondary endpoints included eGFR and Major Adverse Kidney Events (MAKE) up to Day 90. Randomised patients who had received at least one dose of study drug were analysed for primary and safety analyses. Participants, investigators and sponsor were masked to treatment allocation. This study is registered at ClinicalTrials.gov (NCT05126303) and EudraCT (2021-004040-19).

**Findings:**

Patient enrolment was stopped at interim analysis due to futility. Between March 31, 2022 and July 12, 2023, 177 patients (RMC-035: 89, placebo: 88) were randomised and treated. AKI rate for RMC-035 vs placebo was 50.6% (n = 45) and 39.8% (n = 35) (relative risk [RR]: 1.30, 90% confidence interval [90% CI]: 0.99, 1.71; p = 0.12). A short-lived creatinine increase was observed with the higher RMC-035 dose. Treatment with RMC-035 was associated with improved secondary renal outcomes at Day 90: placebo-adjusted eGFR change from baseline 4.3 mL/min/1.73 m^2^, 90% CI 0.51–8.12, p = 0.06; and MAKE 6.7% (n = 6) vs 15.9% (n = 14); RR: 0.41, 90% CI: 0.19, 0.88, p = 0.05. The most frequently reported TEAEs for RMC-035 were chills (30.3%), nausea (21.3%), anaemia (20.2%); and atrial fibrillation (29.5%), anaemia (20.5%), hypervolemia (14.8%) for placebo. The majority of TEAEs in both treatment groups were mild or moderate in severity. In the RMC-035 group, 26 (29.2%) patients experienced at least one severe or life-threatening TEAE and in the placebo group 16 (18.2%) patients. There were 4 deaths per treatment arm (one treatment-related, in placebo group).

**Interpretation:**

In this proof-of-concept study, RMC-035 did not reduce AKI 72 h after cardiac surgery. Evaluations may have been confounded by a drug-induced transient creatinine increase in a subgroup of patients. RMC-035 was associated with improved secondary renal outcomes. These results merit further investigation and should be interpreted with caution, as the study was not powered for these outcomes.

**Funding:**

Guard Therapeutics.

**Research in context**

**Evidence before this study**

We searched PubMed with the terms (“alpha-1-microglobulin” OR “glycoprotein”) AND (“acute kidney injury” OR “cardiac surgery associated acute kidney injury”) AND (phase 3) and filters of (clinical trial OR randomised controlled trial) for articles published in any language up to May 27, 2024. Our search identified no results.

RMC-035 is a novel therapeutic protein mimicking human alpha-1-microglobulin (A1M), harbouring potent anti-oxidative and haem-binding properties. Its efficacy for reducing kidney injury was demonstrated in several preclinical models of ischaemia- and reperfusion injury, among others. In Phase 1 clinical studies, multiple intravenous doses of RMC-035 were considered safe and generally well tolerated at plasma exposures within the range of expected pharmacological activity.

**Added value of this study**

In this proof-of-concept study the efficacy of acute treatment with the investigational drug RMC-035 (A1M mechanism) was evaluated for the first time in humans, specifically in patients undergoing open-chest cardiac surgery up to 90 days post-surgery. The study provided critical information on efficacy and safety of RMC-035 in the target population to guide its further development.

**Implications of all the available evidence**

The primary endpoint analysis in this study does not support an acute treatment benefit of RMC-035. The interpretation of this finding is confounded by an acute and short-lived increase in serum creatinine in a subgroup of patients. By contrast, secondary outcomes suggest an effect of RMC-035 on long-term kidney function. Further studies of RMC-035 are therefore warranted, including exploration of lower dose levels, to confirm its potential to reduce an irreversible loss of kidney function.

## Introduction

Acute kidney injury (AKI), defined by an increase in serum creatinine (SCr) and/or reduction of urine output (UO), is a common complication after cardiac surgery and is associated with an increased morbidity as well as short-term and long-term mortality.^1^, ^2^, ^3^, ^4^, ^5^, ^6^ The pathophysiology is multifactorial and includes ischemia-reperfusion injury, haem-induced toxicity, and oxidative stress amongst others.^7^

A common complication after cardiac surgery is a clinically significant permanent loss of renal function. Observational studies indicate that most patients with predisposing risk factors experience a loss of renal function (estimated glomerular filtration rate; eGFR) when measured in a stable phase after surgery.^8^ In turn, loss of eGFR predisposes for incident chronic kidney disease (CKD) and/or accelerated progression of pre-existing CKD, which may result in end-stage renal disease requiring chronic dialysis treatment or kidney transplantation. Accordingly, preventing or reducing a single episode of kidney stress in conjunction with cardiac surgery may not only impact short-term outcomes, but improve long-term kidney outcomes and reduce comorbidities associated with chronic renal impairment. To that end, there are no such therapies available, and clinical management of AKI mainly focuses on reducing modifiable risk factors and supportive treatment, when required.^9^

RMC-035 is a novel investigational drug that is being developed for reducing the risk for dialysis and/or an irreversible loss of kidney function in patients undergoing open cardiac surgery who are at increased risk for AKI. RMC-035 is a small therapeutic protein, mimicking endogenous alpha-1-microglobulin (A1M), with a biodistribution profile and mechanism appropriate for targeting kidney injuries: reductase activity, free radical scavenging, haem binding, and mitochondrial binding/protection.^10^ It has shown robust efficacy in a plethora of preclinical animal models, also pertinent to cardiac surgery.^11^ The safety and pharmacokinetic profile of RMC-035 were previously evaluated in Phase 1 clinical studies of healthy individuals, patients with renal impairment, and patients undergoing cardiac surgery.^12^ RMC-035 was generally well tolerated and without significant safety concerns.

The current trial (NCT05126303; EudraCT Number: 2021-004040-19) was a phase 2a study evaluating the efficacy and safety of RMC-035 in patients undergoing cardiac surgery. Furthermore, this was the first time efficacy was studied in the clinical setting using the A1M mechanism.

## Methods

The CONSORT reporting guidelines were adhered to for presentation of methods and results.^13^

### Study design

This was a multi-centre phase 2a randomised double-blind adaptive design parallel group study (AKITA study). Details of the study protocol have been described previously.^14^ The study was designed to evaluate the efficacy and safety of RMC-035 in patients at high risk for AKI following open-chest cardiac surgery, with the goal to generate proof-of-concept efficacy data and to guide further clinical development in this patient population. The study was approved by independent ethics committees (approval number 2021-778-f-A for global Principal Investigator's site, Münster, Germany) and institutional review boards and conducted in accordance with the Helsinki declaration.

### Patients

Patients presenting at large cardiovascular surgery units in Europe (Germany, Spain, Czech Republic) and North America (USA and Canada), after having provided written informed consent, were screened for key eligibility criteria as shown in Supplementary Table S1 and included adult patients of both sexes scheduled for non-emergent coronary artery bypass graft surgery and/or valve surgery (single or multiple valves) and/or ascending aorta aneurysm surgery with use of cardiopulmonary bypass. Depending upon the type of surgery, patients had to present with at least one pre-disposing risk factor for AKI (Supplementary File 1). Patients with severe renal impairment eGFR <30 mL/min/1.73 m^2^ or scheduled for emergent surgeries or off-pump surgeries or patients requiring mechanical circulatory support within one week prior to surgery were excluded.

### Randomisation and masking

A centralised computer randomisation system was used for block wise treatment allocation. Baseline stratification factors were screening eGFR (<60 vs ≥ 60 mL/min/1.73 m^2^) and region (North America, Europe). Study drug RMC-035 and placebo were identical in appearance and prepared locally by site staff unblinded to the randomisation assignment. Investigators, study personnel, patients and sponsor staff were blinded to the identity of study drug.

### Procedures

The treatment period consisted of 3 days (Day 1–3) with a subsequent treatment-free follow-up period of 27 days (Day 4–30) and an extended follow-up period of 60 days (Day 31–90). The study scheme and study visits are depicted in Supplementary Figure S1.

Dosing of study drug was performed based on renal function at screening and administration was performed via central venous catheter. Patients with eGFR ≥60 mL/min/1.73 m^2^ received 1.3 mg/kg (per dose) for the first and second dose, followed by 0.65 mg/kg (per dose) for the third, fourth, and fifth dose. Patients with eGFR <60 mL/min/1.73 m^2^ received 0.65 mg/kg (per dose) for all 5 doses. The first infusion of study drug was administered approximately 10 min before the expected initiation of cardiopulmonary bypass. Subsequent administrations started at 6, 12, 24 and 48 h, respectively, after the start of the first infusion. Patients were followed up daily until Day 4, at day of hospital discharge and 30 and 90 days after the first dose.^14^ All measurements of SCr (and corresponding GFR estimations) were performed via a single central laboratory to eliminate any confounding effects due to analytical variability. Data on UO was collected for both study groups up to a maximum of 72 h after surgery, or as long as the patient had a Foley catheter inserted.

### Outcomes

The co-primary efficacy and safety endpoints were AKI (Kidney Disease: Improving Global Outcomes definition) within 72 h after surgery and nature, frequency, and severity of treatment-emergent adverse events (TEAEs).^15^

Secondary short-term efficacy endpoints included time-corrected area under the curve (AUC) of SCr for Day 1–Day 4; AKI severity stage, duration and persistence; occurrence of AKI within 7 days after first dose; and need for renal replacement therapy (RRT) within 72 h and within 7 days after end of surgery.

Secondary endpoints reflecting changes in renal function in a stable phase after surgery included eGFR and SCr change from baseline up to Day 90 and occurrence of major adverse kidney events (MAKE) at Day 30 and 90. MAKE is a binary composite outcome defined by either death, any post-surgery dialysis, or ≥25% eGFR decline from baseline measured on Day 30 (MAKE30) and on Day 90 (MAKE90). Thus, a single patient could meet one or several MAKE criteria. In contrast to death and dialysis, the eGFR component is only considered on the day of measurement and not carried forward from Day 30 to Day 90.

Adverse events (AEs) were collected from initiation of the first study drug administration through the Day 30 study visit. Serious AEs (SAEs) were collected from the signing of informed consent until end-of-study visit. Safety was assessed by TEAEs, i.e. AEs occurring within 72 h after the last dose administration. AEs reported after this time point were considered post-treatment AEs (PTAEs). Changes in serum chemistry, haematology and vital signs were assessed daily until Day 4.

Secondary endpoints not reported here due to premature termination of the study included post-baseline changes in urine albumin to creatinine ratio and urine protein to creatinine ratio. Pharmacokinetics of RMC-035 in plasma and presence, titres and characteristics of antidrug antibodies will be analysed and reported separately.

An independent Data Monitoring Committee (DMC) performed an unblinded review of safety data at regular intervals during the study and a planned interim analysis of the primary endpoint including 50% of the planned randomised patients.

### Statistical analysis

Approximately 268 patients were planned to be dosed with either RMC-035 or placebo in a 1:1 ratio. A 30% relative risk reduction was assumed for AKI based on a placebo rate of 50% leading to test power of 80% to show statistically significant results at a two-sided significance level (alpha) of 0.1. A two-sided alpha level of 0.1 was assessed as appropriate to control for type I error and to support the decision on further development at this stage of development and in view of the selected primary endpoint.

Analyses of efficacy and safety endpoints were conducted in all randomised patients who received at least 1 dose of study drug (=modified intent-to-treat analysis set [mITT]). The primary endpoint AKI and the secondary endpoint MAKE30/90 were analysed by the Cochran-Mantel-Haenszel estimate of the common relative risk (RR) across 4 stratification groups (region and eGFR). The estimate of the common RR, 90% confidence interval (90% CI) utilising Greenland and Robins, and p-value were reported. Additionally, the proportion of patients with AKI or MAKE and its 90% CI were calculated for each treatment group. A sensitivity analysis of AKI was performed using a mixed model of repeat measures (MMRM) separately for the untransformed SCr values. This is a statistical approach used to analyse data where multiple measurements are taken on the same patients over time or under different conditions. This model combines both fixed effects and random effects. Sensitivity analyses of MAKE were performed, utilising cystatin C or a combination of SCr and cystatin C for the assessment of eGFR. The calculation of eGFR was done using the CKD-EPI 2009 formula based upon SCr.^16^

Subgroup analyses by screening eGFR (by eGFR <60 or ≥60 mL/min/1.73 m^2^) were prespecified for AKI and conducted for MAKE and eGFR change from baseline.

All statistical tests were two-sided and tested at the 10% level of significance. More details including description of analyses of other secondary endpoints can be found in Supplementary File 2.

### Role of the funding source

The funder of the study was involved in study design, data analysis, data interpretation, and writing of the report but were not involved in data collection. All authors had full access to the data and had final responsibility for the decision to submit for publication. AZ, TEL and MR verified all data in the study.

## Results

Following a sponsor-blinded data review, the DMC recommended to discontinue patient enrolment due to low probability to demonstrate efficacy on the primary endpoint. The study took place between March 31, 2022 (first patient screened) and July 12, 2023 (last patient followed up).

### Patient disposition, demographics and baseline characteristics

**Fig. 1:**
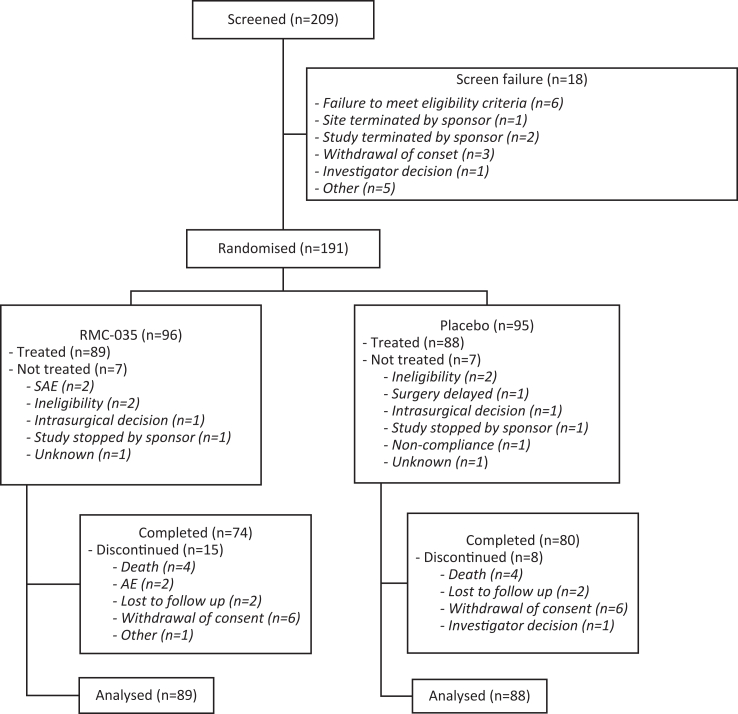
**Disposition of patients**. AE, adverse events.

A total of 209 subjects were screened between with 18 (8.6%) screen failures. Out of 191 patients randomised 177 (92.7%) were treated and included in the analysis: 89 received RMC-035 and 88 received placebo (Fig. 1). Reasons for patients being randomised but not treated were mainly delayed surgery and halt in treatment, screening, and enrolment per Sponsor decision to discontinue the trial. Demographics and baseline characteristics were generally balanced between the treatment groups (Table 1). Follow-up data from 23 patients (RMC-035: 15; Placebo: 8) who had discontinued the study prematurely are not available.

**Table 1:** Summary of demographics and baseline characteristics.

Characteristic	RMC-035 (N = 89)	Placebo (N = 88)
Age (years)		
Mean (SD)	70.2 (8.47)	70.5 (8.06)
Median (IQR)	72.0 (67.0, 76.0)	72.0 (67.0, 76.0)
Sex [n (%)]		
Male	70 (78.7)	69 (78.4)
Female	19 (21.3)	19 (21.6)
Ethnicity [n (%)]		
Hispanic or Latino	2 (2.2)	4 (4.5)
Not Hispanic or Latino	82 (92.1)	81 (92.0)
Not Reported	1 (1.1)	2 (2.3)
Unknown	4 (4.5)	1 (1.1)
Race [n (%)]		
White	86 (96.6)	85 (96.6)
Black or African American	0 (0.0)	0 (0.0)
American Indian or Alaska Native	0 (0.0)	0 (0.0)
Asian	1 (1.1)	2 (2.3)
Native Hawaiian or Other Pacific Islander	0 (0.0)	0 (0.0)
Multiple	0 (0.0)	0 (0.0)
Other	2 (2.2)	1 (1.1)
Not reported	0 (0.0)	0 (0.0)
Weight (kg)		
Mean (SD)	83.3 (18.08)	86.3 (20.49)
Median (IQR)	78.0 (70.4, 93.5)	83.5 (74.0, 96.4)
Country [n (%)]		
United States	4 (4.5)	2 (2.3)
Canada	29 (32.6)	35 (39.8)
Spain	11 (12.4)	12 (13.6)
Germany	33 (37.1)	31 (35.2)
Czech Republic	12 (13.5)	8 (9.1)
Region [n (%)]		
North America	33 (37.1)	37 (42.0)
Europe	56 (62.9)	51 (58.0)
eGFR Day −1 [n (%)]		
≥60 mL/min/1.73 m^2^	55 (61.8)	57 (64.8)
<60 mL/min/1.73 m^2^	34 (38.2)	31 (35.2)
eGFR (mL/min/1.73 m^2^)^a^		
Median (IQR)	77.0 (63.7, 90.2)	80.8 (64.6, 89.9)
Serum creatinine (mg/dL)^b^		
Median (IQR)	0.930 (0.790, 1.110)	0.945 (0.760, 1.070)
Cystatin C (mg/L)^a^		
Median (IQR)	1.110 (0.940, 1.280)	1.090 (0.910, 1.300)
Scheduled Surgery Type [n (%)]^c^		
CABG	40 (44.9)	38 (43.2)
Valve surgery	24 (27.0)	16 (18.2)
Ascending aorta aneurysm surgery	1 (1.1)	2 (2.3)
Combined surgery	24 (27.0)	32 (36.4)
AKI Risk Factors [n (%)]^d^		
3: Type 2 diabetes mellitus	40 (44.9)	45 (51.1)
4: Age ≥70 years	60 (67.4)	58 (65.9)
5: Heart failure NYHA class II or higher	60 (67.4)	54 (61.4)

The mITT set includes all randomised patients who received at least 1 dose of IMP and is based on randomised treatment group.

AKI, acute kidney injury; CABG, coronary artery bypass graft; CKD-EPI, Chronic Kidney Disease-Epidemiology Collaborations; eCRF, electronic case report form; eGFR, estimated glomerular filtration rate; IQR, interquartile range; mITT, modified intent to treat; n/N, number of patients; NYHA, New York Heart Association; SAP, Statistical Analysis Plan; SCr, serum creatinine; SD, standard deviation.

a Calculated utilising the CKD-EPI equation for SCr (2009).

b Analysed at the Central Laboratory.

c Denominator for each Surgery Type percentage = number of patients in mITT set.

d Denominator for each Risk Factor percentage = number of patients in mITT set.

The type of cardiac surgery was similar between the treatment groups, except for valve repair, which was more frequent in the RMC-035 group (Supplementary Table S2).

### Study drug exposure

Thirteen (14.6%) patients in the RMC-035 group and 7 (8.0%) in the placebo group discontinued study treatment. The most frequent reason for discontinuation of study drug was an AE (7 [7.9%] patients in the RMC-035 group; 3 [3.4%] patients in the placebo group), followed by withdrawal of consent (4 [4.5%] patients in the RMC-035 group) (Supplementary Table S3).

### Efficacy

#### Primary endpoint

Numbers (percentages) of patients with AKI in the RMC-035 and placebo arms were 45 (50.6%) and 35 (39.8%) and the overall relative risk (RR) for AKI was 1.30 (90% CI 0.99, 1.71, p = 0.12) for RMC-035 compared to placebo (Table 2). A pre-specified subgroup analysis demonstrated a significantly higher AKI rate for RMC-035 in the eGFR subgroup ≥60 mL/min/1.73 m^2^ who received the higher dose of 1.3 mg/kg (RR 1.66, 90% CI 1.17, 2.35, p = 0.015). By contrast, there was no difference in AKI rate in the pre-defined subgroup eGFR <60 mL/min/1.73 m^2^ who received the lower dose of 0.65 mg/kg (RR 0.85, 90% CI 0.54, 1.35; p = 0.57). There was no difference in AKI risk between the treatment groups by region (Supplementary Table S4).

**Table 2:** Acute kidney injury (Primary Endpoint) overall and by eGFR, duration and stage, persistence and after 7 days and major adverse kidney events at days 30 and 90.

	RMC-035 (N = 89)	Placebo (N = 88)	Relative risk^a^ (90% CI)	p-value^b^
**AKI n (%)**				
Overall	45 (50.6)	35 (39.8)	1.30 (0.99, 1.71)	0.12
By eGFR				
≥60 mL/min/1.73 m^2^	31 (56.4)	20 (35.1)	1.66 (1.17, 2.35)	0.015
<60 mL/min/1.73 m^2^	14 (41.2)	15 (48.4)	0.85 (0.54, 1.35)	0.57
AKI duration [days]^c^				
Mean (SD)	1.5 (2.60)	1.1 (3.04)		
Median (IQR)	1.0 (0.0, 20.0)	0.0 (0.0, 2.0)		0.11
By AKI stage				
Stage 1	31 (68.9%)	22 (62.9%)		
Stage 2	12 (26.7%)	12 (34.3%)		
Stage 3	2 (4.4%)	1 (2.9%)		
AKI persistence^d^	16 (18.0)	13 (14.8)	1.27 (0.72, 2.22)	0.49
AKI (7 days)^e^	54 (60.7)	40 (45.5)	1.35 (1.07, 1.72)	0.035
**MAKE at day 30 n (%)**				
Overall (any MAKE component met)	10 (11.2)	9 (10.2)	1.11 (0.54, 2.29)	0.82
Death through day 30	3 (3.4)	3 (3.4)		
Dialysis through day 30	3 (3.4)	1 (1.1)		
≥25% reduction of eGFR	7 (7.9)	5 (5.7)		
**MAKE at day 90 n (%)**				
Overall (any MAKE component met)	6 (6.7)	14 (15.9)	0.41 (0.19, 0.88)	0.047
Death through day 90	4 (4.5)	4 (4.5)		
Dialysis through Day 90^f^	3 (3.4)	2 (2.3)		
≥25% reduction of eGFR	3 (3.4)	10 (11.4)		

The mITT set includes all randomised patients who received at least 1 dose of IMP and is based on randomised treatment group.

The denominator for the proportions of patients with AKI by stage is the number of patients with an AKI in each treatment group.

AKI, acute kidney injury; CI, confidence interval; CKD-EPI, Chronic Kidney Disease-Epidemiology; eGFR, estimated glomerular filtration rate; IMP, investigational medicinal product; IQR, interquartile range; KDIGO, Kidney Disease: Improving Global Outcomes; MAKE, major adverse kidney events; mITT, modified intent to treat; n/N, number of patients; RRT, renal replacement therapy; SD, standard deviation.

a Relative Risk (RR) is the ratio of risk for AKI or MAKE in RMC-035 group divided by ratio of risk for AKI or MAKE in placebo group (RR <1 indicates decreased risk of AKI or MAKE in patients treated with RMC-035 vs placebo). For MAKE, data is presented as the overall outcome (ie, any MAKE component met) and split by the individual MAKE components. Note that a single patient can meet one or several MAKE criteria. Confidence interval utilises Greenland and Robins.

b Cochran-Mantel-Haenszel estimate with stratification factors of region (North America and Europe) and eGFR at Day −1 (≥60 and < 60 mL/min/1.73 m^2^).

c AKI duration defined as the number of days meeting the definition of AKI starting within 72 h after first dose of study drug until resolution. Denominator is the mITT set.

d AKI persistence defined as an AKI (KDIGO definition) developing within 72 h after first dose of IMP and with a duration of ≥72 h.

e AKI within 7 days after first dose of IMP.

f RRT requirement by Day 7: 1 patient in each treatment group required dialysis within 72 h after end of surgery, and 2 patients in the RMC-035 group and 1 patient in the placebo group required dialysis within 7 days after end of surgery.

#### Secondary short-term efficacy endpoints

Consistent with primary endpoint analyses, the geometric mean AUC of SCr for Day 1–4 was significantly higher for RMC-035 compared to placebo (geometric mean percent difference [90% CI]: 8.43 [3.24, 13.88]; p = 0.0067). Further analyses of short-term changes from baseline in SCr were assessed by pre-defined eGFR subgroups (Supplementary Figure S2). Importantly, the subgroup eGFR ≥60 mL/min/1.73 m^2^ who received the higher RMC-035 dose experienced an acute and short-lived rise in SCr, observed at 12 h after the first dose, persisting up to Day 7 visit (hospital discharge). By contrast, this finding was not observed in the subgroup eGFR <60 who received the lower dose.

Results on other secondary endpoints are presented in Table 2. The median duration of AKI was 1.0 in the RMC-035 group and 0.0 days in the placebo group (p = 0.11). Most AKI patients had Stage 1 or Stage 2 AKI. The risk of AKI persistence did not significantly differ between the RMC-035 group (n = 16 [18.0%]) and the placebo group (n = 13 [14.8%]; RR 1.27 [90% CI 0.72, 2.22]; p = 0.49). Numbers of patients requiring RRT within 72 h and 7 days after surgery were low and balanced.

#### Secondary efficacy endpoints reflecting changes in renal function in a stable phase after surgery

**Fig. 2:**
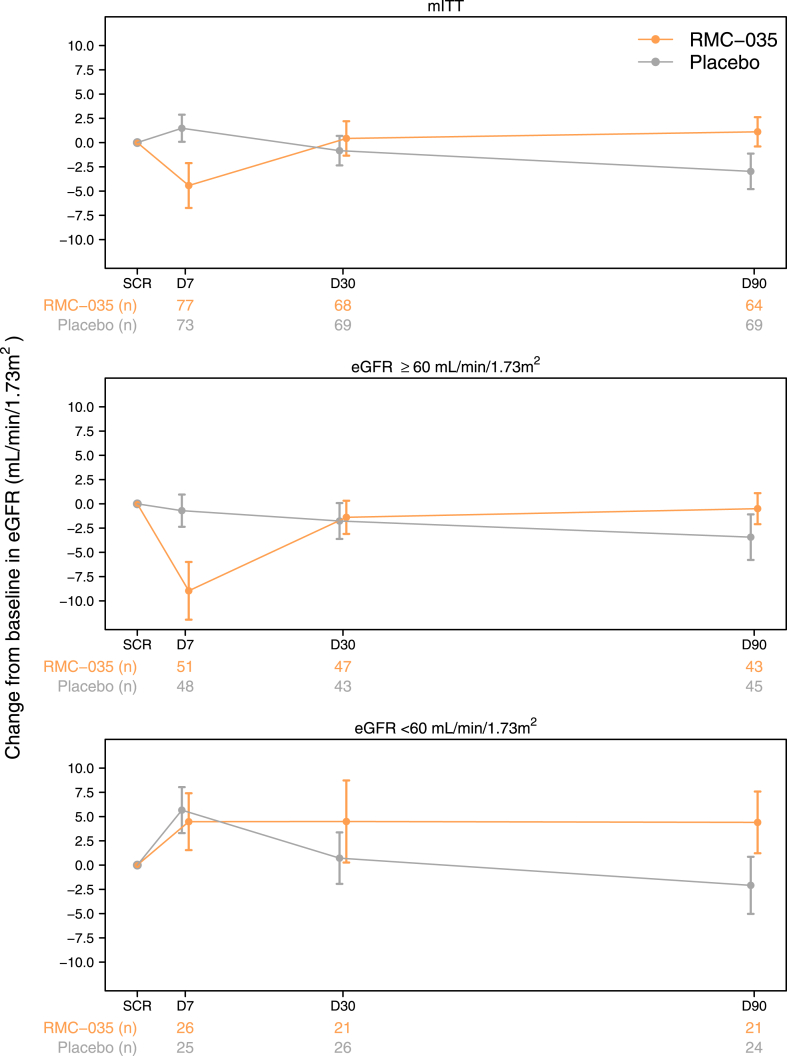
**Mean eGFR changes from baseline at Day 7, 30 and 90 overall and by eGFR subgroups**. Upper panel shows the change from baseline in eGFR (mL/min/1.73 m^2^) at randomisation, Day 7, 30 and 90 of all patients randomised to placebo or RMC-035. Middle panel shows the change from baseline in eGFR (mL/min/1.73 m^2^) at randomisation, Day 7, 30 and 90 of patients with a baseline eGFR ≥60 mL/min/1.73 m^2^. Lower panel shows the change from baseline in eGFR (mL/min/1.73 m^2^) at randomisation, Day 7, 30 and 90 of patients with a baseline eGFR <60 mL/min/1.73 m^2^. Orange: RMC-035; Grey: Placebo. Data are shown as mean ± standard error. SCR: screening; D7: Day 7; D30: Day 30; D90: Day 90; n: number of patients.

Mean eGFR changes from baseline at Day 7, 30 and 90 overall and by eGFR subgroups are presented in Fig. 2. A transient dip in eGFR was observed for RMC-035 on Day 7, however, subgroup analyses demonstrated that this was only present in the subgroup eGFR ≥60 mL/min/1.73 m^2^ receiving the higher dose and was fully reversible with gradual eGFR improvements compared to placebo during follow-up. The measured net difference between RMC-035 and placebo at Day 90 was 4.1 mL/min/1.73 m^2^ which was significant in the MMRM (4.3 mL/min/1.73 m^2^, 90% CI 0.51, 8.12, p = 0.06). The eGFR improvement at Day 90 with RMC-035 was more pronounced in the subgroup eGFR <60 mL/min/1.73 m^2^ who received the lower dose that did not cause an acute SCr increase. At Day 90 the measured net eGFR difference between RMC-035 and placebo was 6.5 mL/min/1.73 m^2^ (eGFR <60 mL/min/1.73 m^2^) and 2.9 mL/min/1.73 m^2^ (eGFR ≥60 mL/min/1.73 m^2^), respectively. This finding was consistent in the MMRM, with a significant improvement of eGFR for RMC-035 in the subgroup eGFR <60 mL/min/1.73 m^2^ (7.9 mL/min/1.73 m^2^, 90% CI 1.26, 14.55, p = 0.05).

Results on MAKE30/90 are presented in Table 2. There was a significant 59% reduction of MAKE90 with RMC-035 vs placebo (RR 0.41 [90% CI 0.19, 0.88]; p = 0.047). Sensitivity analyses confirmed these findings, and subgroup analysis demonstrated a significant reduction of MAKE90 in the subgroup of eGFR ≥60 mL/min/1.73 m^2^ (Supplementary Tables S5 and S6).

### Safety

A summary of frequently reported TEAEs (≥10% in either treatment group) is shown in Supplementary Table S7. The most frequently reported TEAEs by PT were chills (30.3%), nausea (21.3%), and anaemia (20.2%) for RMC-035, and atrial fibrillation (29.5%), anaemia (20.5%), and hypervolemia (14.8%) for placebo. TEAEs were similar across treatment groups except for chills, nausea, and pyrexia, which were more frequently reported for RMC-035 and occurred during or after administration of the fourth or fifth dose of study drug as infusion-related reactions (IRR) (Supplementary Table S8). The majority of TEAEs in both treatment groups were mild or moderate in severity. In the RMC-035 group, 26 (29.2%) patients experienced at least one severe or life-threatening TEAE and in the placebo group 16 (18.2%) patients (Table 3). The most frequently occurring treatment-emergent serious adverse events in the RMC-035 arm were five cases of hypertensive crisis reported during or shortly after the administration of the last dose of study drug (Table 4). No PTAEs occurred with a frequency ≥10% in any treatment group.

**Table 3:** Summary of treatment-emergent adverse events by maximum severity.

Parameter	RMC-035 (N = 89)		Placebo (N = 88)	
	Patients n (%)	Events n	Patients n (%)	Events n
Maximum NCI CTCAE v5.0 severity				
1—Mild	10 (11.2)	247	22 (25.0)	192
2—Moderate	39 (43.8)	192	26 (29.5)	96
3—Severe	14 (15.7)	39	10 (11.4)	17
4—Life-threatening	12 (13.5)	21	6 (6.8)	8
5—Death	1 (1.1)	1	2 (2.3)	2

TEAE, treatment-emergent adverse event; NCI CTCAE v5.0, National Cancer Institute Common Terminology for Adverse Events Version 5.0.

Patients reporting more than one TEAE are counted only once in the Patients column in the row of the greatest severity among all reported TEAEs for that patient.

**Table 4:** Summary of most frequently occurring (≥3 Patients) treatment-emergent serious adverse events.

System organ class preferred term n (%)	RMC-035 (N = 89)	Placebo (N = 88)
Patients with any SAE	20 (22.5)	15 (17.0)
Blood and lymphatic system disorders	5 (5.6)	0 (0.0)
Coagulopathy	4 (4.5)	0 (0.0)
Cardiac disorders	7 (7.9)	8 (9.1)
Cardiac arrest	2 (2.2)	2 (2.3)
Cardiac tamponade	2 (2.2)	1 (1.1)
Vascular disorders	7 (7.9)	1 (1.1)
Hypertensive crisis	5 (5.6)	0 (0.0)

The Safety Analysis Set includes all randomised patients who received at least 1 dose of IMP and is based on treatment actually received.

SAE is any AE that results in death, is life-threatening, requires inpatient hospitalisation or prolongation of existing hospitalisation, results in persistent disability/incapacity, is a congenital anomaly/birth defect, or is another medically important serious event.

AE, adverse event; IMP, investigational medicinal product; n/N, number of patients; SAE, serious adverse event.

A total of 8 patients (4.2%) died during the study period: 4 patients (4.2%) in the RMC-035 group and 4 patients (4.2%) in the placebo group (Table 2). TEAEs leading to death (not shown) were cardiac arrest (1 patient in the placebo group), systemic inflammatory response syndrome (1 patient in the RMC-035 group), and pneumonia (1 patient in the placebo group). No deaths in the RMC-035 group were reported as related to study drug. For placebo, the only fatal TEAE considered possibly related to study drug was cardiac arrest (1 patient). PTAEs leading to death (not shown) were acute cardiac failure (1 patient in the RMC-035 group), multiple organ dysfunction syndrome (1 patient in each treatment group), and septic shock failure (1 patient in each treatment group). Local safety laboratory and vital signs monitoring identified no differences between the treatment groups in changes from baseline to Day 4 apart from eGFR (Supplementary Tables S9 and S10).

## Discussion

The principal objective of this randomised, placebo-controlled, phase 2 trial was to evaluate the safety and renoprotective effect of a novel therapeutic A1M protein, RMC-035, in patients undergoing cardiac surgery. Whilst RMC-035 did not reduce post-operative AKI, analyses of secondary outcomes (eGFR, MAKE) supported its hypothesised kidney-protective effect in a stable phase after surgery.

Since a thorough dose-range evaluation was not a study objective, a start dose of 1.3 mg/kg was chosen. This was assessed as the maximum safe dose based on available clinical and nonclinical safety evaluations of RMC-035, whilst providing the greatest likelihood to demonstrate efficacy. A reduced start dose (0.65 mg/kg) was given to patients with pre-operative renal impairment due to the potential risk for kidney tubular overload (causing a transient SCr increase) as consistently identified in nonclinical toxicology studies. The lower dose was still predicted to be higher than the maximum efficacious dose.

The population studied was representative of a high-risk cardiac surgery population with regards to age and sex.

Analysis of the primary endpoint AKI within 72 h after surgery unexpectedly showed a clear trend towards higher AKI rate with RMC-035, attributed to a higher incidence of non-persistent mild AKI (stage 1). For this reason, patient enrolment was prematurely discontinued. However, further analyses indicated a differential AKI risk in the pre-defined subgroups of baseline eGFR who received different doses of study drug: the higher RMC-035 dose was associated with a significantly higher AKI rate, whereas AKI was numerically reduced with the lower dose. Moreover, the higher RMC-035 dose triggered an acute short-lived SCr increase, which was not observed with the lower dose. Taken together, these results support that the higher dose induced the potential risk of tubular RMC-035 overload (i.e. overexposure), which confounded the analyses of the primary and short-term secondary SCr-based endpoints.

The mechanism of the acute short-lived SCr increase observed with the higher RMC-035 dose is well understood based on toxicological evaluations. The kidney is the main target organ for RMC-035, and overexposure leads to its tubular precipitation, which resolves spontaneously after treatment discontinuation. This was noted in both rodents and non-human primates, with histopathological assessments demonstrating tubular precipitates of RMC-035 at high peak exposures (data not shown). Tubular precipitation of proteins leading to SCr increase is not unique to RMC-035 and is clinically known in other conditions such as multiple myeloma.^17^^,^^18^ Importantly, acute SCr changes were not observed in previous Phase 1 studies in healthy individuals at the same dose level. This indicates that patients undergoing cardiac surgery are more susceptible to tubular overload than healthy individuals, and that the exposure threshold for this event is likely to be close to the associated dose of 1.3 mg/kg. This is further supported by the current study results where the lower dose of 0.65 mg/kg did not elicit a SCr increase when administered to patients with CKD and/or renal impairment (eGFR <60 mL/min/1.73 m^2^), i.e., patients which are considered particularly susceptible to tubular overload. Furthermore, post-hoc pharmacokinetic-pharmacodynamic modelling demonstrated a clear threshold above 0.65 mg/kg, resulting in an exposure-dependent increase in SCr (data not shown). Overall, these findings provide the basis for defining 0.65 mg/kg as the maximum safe and efficacious dose in patients undergoing cardiac surgery.

Although RMC-035 did not reduce post-operative AKI, it is essential to contextualise the choice of primary endpoint in this phase 2a trial. The definition of AKI stage 1 may not be regarded as clinically relevant in the post-operative setting and is not an acceptable endpoint for regulatory approval, albeit small elevations of SCr or AKI stage 1 after cardiac surgery are associated with long-term adverse events.^19^, ^20^, ^21^ Therefore, AKI stage 1 or higher was chosen as surrogate for long-term kidney outcomes, including the anticipated endpoint MAKE90 in a confirmatory phase 3 study. This strategy enabled a lower sample size than would have been required for MAKE90, whilst providing acceptable power for secondary efficacy endpoints like eGFR change from baseline.

Importantly, evaluation of such secondary endpoints supported the hypothesised kidney-protective effect of RMC-035. Significant and clinically relevant improvements in eGFR, above 4 mL/min/1.73 m^2^, were observed in the overall RMC-035 group compared to placebo at Day 90. Subgroup analysis indicated a greater eGFR effect in the subgroup eGFR <60 mL/min/1.73 m^2^ (effect size of 6–8 mL/min/1.73 m^2^) who received the lower start dose not causing an acute SCr increase (i.e. overexposure). It remains to be determined whether patients with lower renal function (including patients with CKD) respond relatively better to treatment with RMC-035, or if the greater eGFR benefit is attributed to a lower and more appropriate dose not causing (tubular) overexposure. This question will need to be addressed in a separate dose-finding study.

Consistent with the eGFR improvement at Day 90 relative to placebo, RMC-035 treatment also reduced MAKE90, the anticipated phase 3 endpoint to support regulatory approval. Importantly, MAKE90 reduction with RMC-035 was explained by a lower proportion of patients who experienced at least 25% eGFR decline or more. Taken together, RMC-035 has the potential to prevent an overall loss of renal function (eGFR) on the population level, as well as reducing the number of patients who experience a severe loss of renal function of at least 25% (MAKE90).

Despite improvements of kidney outcomes at Day 90, irrespective of start dose of RMC-035, it is conceptually possible that doses lower than 1.3 mg/kg are renoprotective, whereas higher doses may cause harm to the kidneys in the short-term, or off-set a pharmacological benefit, due to its tubular precipitation. This could be especially important in patients with low residual renal function. This question remains unanswered since, in the face of an acute SCr increase, the subgroup who received the higher RMC-035 dose still experienced a numerical eGFR improvement at Day 90, and a statistically significant reduction of MAKE90 vs placebo, without any other renal safety signals beyond an increased frequency of biochemical AKI stage 1. Nevertheless, a short-term insult followed by complete recovery facilitated by a higher renal function at baseline cannot be excluded for the higher dose group. This risk will be appropriately mitigated in future studies of RMC-035 by administering a clinically effective dose that will not result in a short-lived SCr increase.

The apparent disconnect between short- and long-term renal outcomes in this study merits further attention.

Whereas AKI is a recognised predictor of long-term kidney outcomes, recent studies, including this one, support that a clinically relevant permanent loss of kidney function may occur without an AKI diagnosis, and vice versa.^22^ Thus, the relevance of mild AKI as a marker of true and progressive tissue kidney injury may therefore be challenged, and may vary depending on clinical setting and underlying aetiology. The limited sensitivity and specificity of especially AKI stage 1 for predicting long-term renal outcomes after cardiac surgery may be attributed to tissue-specific events, for example maladaptive kidney tubular cell repair mechanisms, which are not appropriately captured or characterised by transient changes in SCr or UO.

The safety profile of RMC-35 was generally in line with the expected background in this patient population regarding the nature and frequency of AEs, including an equal distribution of deaths between study drug and placebo. AEs clustered as IRRs were however more frequently reported with RMC-035 than placebo. These were mostly of mild or moderate severity, but included several reports of hypertensive crisis, all of which occurred after the last (fifth) drug administration. The aetiology of these events remains to be fully determined but is expected to be mitigated by adjustment of dosing regimen without loss of efficacy, since the predicted maximum efficacious dose (and required frequency of dosing) in nonclinical studies of RMC-035 is substantially lower than what was used in this study.

The overall efficacy findings and interpretation of results are strengthened by the fact that baseline demographics, AKI risk factors, type of surgical procedure as well as intra-operative risk factors were generally balanced between RMC-035 and placebo. There were also no apparent differences in intercurrent events during follow-up, or differences in use of drugs with hemodynamic actions on the kidney, e.g. blockers of the renin-angiotensin system or sodium-phosphate glucose co-transporter type 2, that would account for the observed eGFR and MAKE90 effect.

The study was not without limitations. In particular, the data should be interpreted with caution since the preplanned sample size was not reached due to early termination following the interim analysis. Further, the study was not powered for secondary endpoints or confirmatory conclusions, and no adjustment for multiplicity was performed.

In conclusion, therapeutic A1M delivery (RMC-035) did not reduce AKI after cardiac surgery, but was associated with clinically meaningful kidney-related secondary outcomes, including eGFR and MAKE at Day 90. Further studies of RMC-035 are warranted, including exploration of lower dose levels, to confirm its potential to improve kidney outcomes.

## Contributors

Participated in the design of the study: AZ, TEL, AB, JLK, DE

Analysed and interpreted the data: AZ, TEL, JLK, DE, MR.

Wrote the first draft of the manuscript: AZ, TEL, MR.

Revised the manuscript and provided scientific input: NN, CDM, JoB, ML, KM, JaB, BdV, AM, AB, JLK, DE, MT.

All authors had full access to the data and had final responsibility for the decision to submit for publication. AZ, TEL and MR verified all data in the study.

## Data sharing statement

Data collected for the study including individual patient data will not be made available.

## Declaration of interests

AZ received consulting and lecture fees and grants from German Research Foundation, BioMerieux, Baxter, Fresenius, Paion, Viatris, Bayer, Novartis, AM Pharma, and Alexion, received meeting and travel support from Sphingotec and held leadership and fiduciary roles at the German Interdisciplinary Association for Intensive and Emergency Medicine, the International Anesthesia Research Society and the journal “Der Anästhesist”. JLK received consulting fees from Biomerieux, Baxter, Alexion, SeaStar, Novartis, Guard Therapeutics and research funding from the NIH, Fresenius Medical and Biomerieux. TEL is a shareholder and employee of Guard Therapeutics. MR holds stock options and is a paid consultant to Guard Therapeutics. CDM received consulting fees from Alexion. AB was past-president of the German society for heart, thorax and vascular surgery, served on the DSMB for the INCREASE study and received consulting, lecture and advisory board fees from Guard Therapeutics, Marizyme, Abbott, and Abiomed, Data Monitoring Committee payments from Amopharma and patient fees from Guard Therapeutics. JoB received travel costs payments from Guard Therapeutics for an investigator meeting. DE is president of the ERAS Cardiac Society and was a member of Data Safety Monitoring Boards/Advisory Boards for Arthrex, Edwards Lifescience, Medella, Atricure, Pharmacosmos, Renibus, Alexion, and Genentech. AM, BdV, JaB, KM, ML, MT and NN declare no competing interests.

## Appendix A Supplementary data

Full Supplement

List of collaborators
