# Supplementary material for: Efficacy and safety of therapeutic alpha-1-microglobulin RMC-035 in reducing kidney injury after cardiac surgery: a multicentre, randomised, double-blind, parallel group, phase 2a trial
Source: eClinicalMedicine. 2024 Sep 16;76:102830. doi: 10.1016/j.eclinm.2024.102830 (PMC11421351; doi:10.1016/j.eclinm.2024.102830)
Supplement: Full Supplement [file mmc1.pdf]

Contents of Supplement

List of AKITA Investigators and Collaborators.....2
Supplementary Table 1: Key Eligibility Criteria.....4
Supplementary File 1: List of AKI risk factors.....5
Supplementary Table 2: Summary of Surgical Details.....6
Supplementary Table 3: Summary of Exposure.....7
Supplementary Table 4: Acute Kidney Injury by Region.....8
Supplementary Table 5: Major Adverse Kidney Events (Sensitivity Analyses based upon Cystatin C).....9
Supplementary Table 6: Major Adverse Kidney Events (Sensitivity Analyses based upon Serum Creatinine and Cystatin C).....10
Supplementary Table 7: Summary of Most Frequently Reported (≥10%) Treatment-Emergent Adverse Events.....11
Supplementary Table 8: Summary of Treatment-Emergent Adverse Events Suggestive of Infusion Related Reactions on Days of Treatment.....12
Supplementary Table 9: Summary of Changes from Baseline to Day 4 in Safety Laboratory Values.....13
Supplementary Table 10: Summary of Changes from Baseline to Day 4 in Vital Signs.....14
Supplementary Figure 1: Study Flow Chart.....15
Supplementary Figure 2: Change from Baseline in Serum Creatinine by eGFR Subgroup.....16
Supplementary File 2: Statistical Analysis Plan.....17

List of AKITA Investigators and Collaborators

AKITA Investigators

Country	Last Name	First Name	Institution	City
Canada	Brown	Craig	New Brunswick Heart Centre (St John's)	New Brunswick
Canada	de Varennes	Benoit	McGill University Health Centre - Royal Victoria Hospital (MUHC)	Montreal
Canada	Laflamme	Maxime	Institut Universitaire de Cardiologie et de Pneumologie de Québec (IUCPQ)	Quebec
Canada	Lamy	Andre	Hamilton General Hospital	Hamilton
Canada	Mazer	David	Saint Michael’s Hospital	Toronto
Canada	Noiseux	Nicolas	Centre hospitalier de l'Université de Montréal (CHUM)	Montreal
Czech Republic	Burkert	Jan	Fakultni nemocnice v Motole	Prague
Czech Republic	Vojacek	Jan	Fakultní nemocnice Hradec Králové	Hradec Králové
Germany	Böhm	Johannes	Deutsches Herzzentrum München	Munich
Germany	Böning	Andreas	University of Gießen	Giessen
Germany	Helms	Sven	Herz- und Diabeteszentrum Nordrhein-Westfalen (NRW)	Bad Oeynhausen
Germany	Matschke	Klaus	Herzzentrum Dresden GmbH	Dresden
Germany	Szabo	Gabor	Universitätsklinikum Halle (Saale)	Halle
Germany	Thielmann	Matthias	Universitätsklinikum Essen	Essen
Germany	Wahlers	Thorsten	Universitätsklinikum Köln	Köln
Germany	Zarbock	Alexander	Universitätsklinikum Münster	Münster
Spain	Adrio Nazar	Belén	Complejo Hospitalario Universitario de Santiago (CHUS) - Hospital Clínico Universitario	Santiago
Spain	Ginel Iglesias	Antonino	Hospital de la Santa Creu i Sant Pau	Barcelona
Spain	Muñiz Albaiceta	Guillermo	Hospital Universitario Central de Asturias (HUCA)	Oviedo
Spain	Muñoz Carvajal	Ignacio	Hospital Universitario Reina Sofía	Cordoba
Spain	Reyes Copa	Guillermo	Hospital Universitario de La Princesa	Madrid
USA	Corteville	David	Rochester Regional Health - Rochester General Hospital	Rochester, NY
USA	East	Cara	Baylor Scott and White Research Institute - Dallas	Dallas, TX
USA	Kress	David	Aurora Health Care - Aurora St. Luke’s Medical Center	Milwaukee. WI
USA	Osaki	Saturo	University of Wisconsin	Madison, WI
USA	Scavo	Vincent	Indiana Ohio Heart (Lutheran medical group)	Fort Wayne, IN
USA	Shelstad	Ryan	Bryan Heart	Lincoln, NE
USA	Swaminathan	Madhav	Duke University Hospital	Durham, NC
USA	Teman	Nicholas	University of Virginia (UVA) Health - University Hospital	Charlottesville, VA

AKITA Scientific Advisory Board

Country	Last Name	First Name	Institution	City
Germany	Böning	Andreas	University of Gießen	Giessen
USA	Engelman	Daniel	Baystate Medical Center – Department of Surgery	Springfield, MA
USA	Koyner	Jay L	University of Chicago – Section of Nephrology	Chicago, IL
Italy	Ronco	Claudio	San Bortolo Hospital and International Renal Research Institute – Department of Nephrology	Vicenza
Germany	Zarbock	Alexander	Universitätsklinikum Münster	Münster

AKITA Data Monitoring Committee (DMC)

Country	Last Name	First Name	Institution	City
Belgium	Lameire	Norbert	Professor Emeritus of Medicine & Nephrology	Destelbergen
USA	Mehta	Ravindra	University of California San Diego – Department of Medicine	San Diego, CA
USA	Irish	William	Imperium Statistical Consulting LLC	Winterville, NC

Supplementary Table 1: Key Eligibility Criteria

Patients who met all inclusion and no exclusion criteria were eligible for enrollment into the study.	
Inclusion Criteria:	
	Age was ≥18 and <85 years
	Estimated glomerular filtration rate was ≥30 mL/min/1·73 m² (at screening) using the CKD-EPI equation with SCr
	Scheduled for non-emergent CABG surgery AND/OR valve surgery (single or multiple valves) AND/OR ascending aorta aneurysm surgery with use of CPB AND AKI risk factors were present (at screening).<br>i) If only one type of surgery was scheduled, at least 2 AKI risk factors were present OR eGFR was <60 mL/min/1.73m2 (at screening) with or without additional risk factors<br>ii) If any combined surgery was scheduled, at least 1 AKI risk factor was present
Exclusion criteria	
	Scheduled for emergent surgeries
	Scheduled for CABG and/or valve surgery and/or ascending aorta aneurysm surgery combined with additional non-emergent cardiac surgeries (eg, congenital heart defects)
	Scheduled to undergo transcatheter aortic valve implantation (TAVI) or transcatheter aortic valve replacement (TAVR), or off-pump surgeries or left ventricular assist device (LVAD) implantation
	Experienced a cardiogenic shock or hemodynamic instability which required inotropes or vasopressors or other mechanical devices such as intra-aortic balloon counter-pulsation (IABP) within 24 hours prior to surgery
	Had a requirement for any of the following within 1 week prior to surgery: defibrillator or permanent pacemaker, mechanical ventilation, IABP, LVAD, other forms of mechanical circulatory support (MCS)
	Diagnosed with AKI (as defined by KDIGO criteria) within 3 months prior to surgery
	Required cardiopulmonary resuscitation within 14 days prior to cardiac surgery
	Had ongoing sepsis (as defined by SEPSIS-3, the Third International Consensus Definitions for Sepsis and Septic Shock) within the past 2 weeks or, in the opinion of the Investigator, an untreated diagnosed clinically significant infection (viral or bacterial) prior to or at screening and before randomization
	Total bilirubin or alanine aminotransferase (ALT) or aspartate aminotransferase (AST) ≥2× the upper limit of normal (ULN) at screening
	History of solid organ transplantation or RRT
	Medical condition which required active immunosuppressive treatment
	Severe allergic asthma defined as confirmed diagnosis of asthma poorly controlled while receiving high-dose inhaled corticosteroid treatment, or with requirement of a high level of treatment to maintain control
	Ongoing chemotherapy or radiation therapy for malignancy that may have impacted kidney function
	Had received an IMP within the last 90 days (or within 5 half-lives of an investigational drug, whichever is longer)
	Known allergy to RMC-035 or one of its constituents, or had previously received RMC-035

Supplementary File 1: List of AKI risk factors

• Left ventricular ejection fraction (LVEF) <35% at any time during the 3-month period before or at the time of screening as assessed by either echocardiography, cardiac magnetic resonance imaging (MRI), or nuclear scan
• Repeat surgery/history of previous open chest cavity cardiac surgery with or without CPB
• Confirmed diagnosis of type 2 diabetes (T2DM) at least 3 months prior to screening AND ongoing treatment with an approved anti-diabetic drug
• Age ≥70 years at the time of screening
• Heart failure New York Heart Association (NYHA) class II or higher at any time during the 3-month period before or at the time of screening
• Documented history of previous AKI as per KDIGO criteria longer than 3 months before date of screening independent of the etiology of AKI
• Anemia with hemoglobin ≤11 g/dL at any time during the 3-month period before or at the time of screening
• Albuminuria, defined as UACR >100 mg/g in a spot urine sample or >100 mg/24 hours in a 24-hour urine collection at any time during the 3-month period before or at the time of screening
• Estimated glomerular filtration rate was <60 mL/min/1·73 m2 using the CKD-EPI equation with serum creatinine at the time of screening

Supplementary Table 2: Summary of Surgical Details

Characteristic	RMC-035 (N=89)	Placebo (N=88)
Type of Surgery [n (%)]		
CABG	57 (64·0)	62 (70·5)
Valve Replacement	41 (46·1)	43 (48·9)
Valve Repair	12 (13·5)	5 (5·7)
Aortic Repair	9 (10·1)	12 (13·6)
Total Surgery Duration (minutes)[a]		
Mean (SD)	216·9 (61·80)	224·7 (84·36)
Median (IQR)	208·0 (166·0, 251·0)	202·5 (172·5, 252·0)
Cardiopulmonary Bypass Details		
Type [n (%)]		
Pulsatile	13·0 (14·6)	18 (20·5)
Non-pulsatile	76 (85·4)	70 (79·5)
Duration (minutes)[b]		
Mean (SD)	104·1 (43·83)	108·1 (55·62)
Median (IQR)	94·0 (75·0, 130·0)	95·0 (73·0, 124·0)
Duration of Cross-Clamp (minutes)[c]		
Mean (SD)	76·4 (35·29)	81·4 (44·20)
Median (IQR)	66·0 (51·0, 98·0)	74·0 (52·0, 94·0)

CABG = coronary artery bypass graft; IQR = interquartile range; n/N = number of subjects; SD = standard deviation.
[a] Surgery duration = skin closure date/time – initial skin incision date/time.
[b] Duration of cardiopulmonary bypass = cardiopulmonary bypass end date/time - cardiopulmonary bypass start date/time.
[c] Duration of cross-clamp = cross-clamp release date/time - cross-clamp vessel occlusion date/time.

Supplementary Table 3: Summary of Exposure

Parameter	RMC-035 (N=89)	Placebo (N=88)
Total Number of Doses Received [n (%)]		
n	89	88
1	2 (2·2)	0 (0·0)
2	3 (3·4)	3 (3·4)
3	3 (3·4)	1 (1·1)
4	7 (7·9)	5 (5·7)
5	74 (83·1)	79 (89·8)
Absolute Dose Received (mg) [mean (SD)]		
Dose 1		
eGFR ≥60 mL/min/1·73 m^2	108·4 (23·24)	116·7 (29·13)
eGFR <60 mL/min/1·73 m^2	53·9 (12·20)	51·8 (9·49)
Dose 2		
eGFR ≥60 mL/min/1·73 m^2	108·6 (23·38)	116·6 (29·39)
eGFR <60 mL/min/1·73 m^2	53·9 (12·64)	51·8 (9·49)
Dose 3	54·3 (12·03)	55·9 (13·57)
Dose 4	54·2 (11·71)	56·2 (13·60)
Dose 5	53·6 (11·70)	55·5 (13·46)
Reasons for Discontinuation of Study Treatment		
Adverse Event	7 (7·9)	3 (3·4)
Withdrawal of Consent	4 (4·5)	0 (0·0)
Investigator Decision	1 (1·1)	0 (0·0)
Subject placed on RRT	0 (0·0)	1 (1·1)
Subject placed on MCS or ECMO	0 (0·0)	2 (2·3)
Other	1 (1·1)	1 (1·1)

ECMO = extracorporeal membrane oxygenation; MCS = mechanical circulatory support; n/N = number of subjects; RRT = renal replacement therapy; SD = standard deviation.

Supplementary Table 4: Acute Kidney Injury by Region

AKI Events [n (%)]	RMC-035 (N=89)	Placebo (N=88)	Relative Risk [a] (90% CI)	p-value [b]
Overall	45 (50·6)	35 (39·8)	1·30 (0·99, 1·71)	0·12
Subgroup: Region				
North America	19 (57·6)	19 (51·4)	1·13 (0·79, 1·62)	0·57
Europe	26 (46·4)	16 (31·4)	1·47 (0·97, 2·23)	0·12

AKI = acute kidney injury; CI = confidence interval; eGFR = estimated glomerular filtration rate; IMP = investigational medicinal product; mITT = modified intent to treat; n/N = number of subjects.
The mITT set includes all randomized subjects who received at least 1 dose of IMP and is based on randomized treatment group.
[a] Relative Risk (RR) is the ratio of risk for AKI in RMC-035 group divided by risk for AKI in placebo group (RR<1 indicates decreased risk of AKI in subjects treated with IMP versus placebo). Confidence interval utilizes Greenland and Robins.
[b] Cochran-Mantel-Haenszel estimate with stratification factors of region (North America and Europe) and eGFR at Day -1 (≥60 and <60 mL/min/1.73m²).

Supplementary Table 5: Major Adverse Kidney Events (Sensitivity Analyses based upon Cystatin C)

MAKE Events [n (%)]	RMC-035 (N=89)	Placebo (N=88)	Relative Risk (90% CI)	p-value[c]
**MAKE at Day 30[a]**				
Overall	17 (19·1)	20 (22·7)	0·84 (0·52, 1·37)[b]	0·57
Death through Day 30	3 (3·4)	3 (3·4)		
Dialysis through Day 30	3 (3·4)	1 (1·1)		
≥25% reduction of eGFR	16 (18·0)	17 (19·3)		
**MAKE at Day 90[a]**				
Overall	15 (16·9)	24 (27·3)	0·62 (0·39, 1·00)[b]	0·097
Death through Day 90	4 (4·5)	4 (4·5)		
Dialysis through Day 90	3 (3·4)	2 (2·3)		
≥25% reduction of eGFR	14 (15·7)	20 (22·7)		

CI = confidence interval; CKD-EPI = Chronic Kidney Disease-Epidemiology; eGFR = estimated glomerular filtration rate; IMP = investigational medicinal product; MAKE = major adverse kidney events; mITT = modified intent to treat; n/N = number of subjects.

The mITT set includes all randomized subjects who received at least 1 dose of IMP and is based on randomized treatment group.

[a] MAKE at Day 30/90 defined as death through Day 30/90, any dialysis by Day 30/90, or a 25% reduction of eGFR at Day 30/90 compared to baseline using CKD-EPI equations for serum creatinine only.

[b] Relative Risk (RR) is the ratio of risk for MAKE at Day 30 in RMC-035 group divided by ratio of risk for MAKE at Day 30 in placebo group (RR<1 indicates decreased risk of MAKE at Day 30 in subjects treated with IMP versus placebo). Confidence interval utilizes Greenland and Robins.

[c] Cochran-Mantel-Haenszel estimate with stratification factors of region (North America and Europe) and eGFR at Day -1 (≥60 and <60 mL/min/1·73 m²).

Supplementary Table 6: Major Adverse Kidney Events (Sensitivity Analyses based upon Serum Creatinine and Cystatin C)

MAKE Events [n (%)]	RMC-035 (N=89)	Placebo (N=88)	Relative Risk (90% CI)	p-value[c]
**MAKE at Day 30[a]**				
Overall	13 (14·6)	12 (13·6)	1·09 (0·59, 1·99)[b]	0·83
Death through Day 30	3 (3·4)	3 (3·4)		
Dialysis through Day 30	3 (3·4)	1 (1·1)		
≥25% reduction of eGFR	11 (12·4)	8 (9·1)		
**MAKE at Day 90[a]**				
Overall	10 (11·2)	20 (22·7)	0·50 (0·28, 0·88)[b]	0·041
Death through Day 90	4 (4·5)	4 (4·5)		
Dialysis through Day 90	3 (3·4)	2 (2·3)		
≥25% reduction of eGFR	7 (7·9)	15 (17·0)		

CI = confidence interval; CKD-EPI = Chronic Kidney Disease-Epidemiology; eGFR = estimated glomerular filtration rate; IMP = investigational medicinal product; MAKE = major adverse kidney events; mITT = modified intent to treat; n/N = number of subjects.
The mITT set includes all randomized subjects who received at least 1 dose of IMP and is based on randomized treatment group.
[a] MAKE at Day 30/90 defined as death through Day 30/90, any dialysis by Day 30/90, or a 25% reduction of eGFR at Day 30/90 compared to baseline using CKD-EPI equations for serum creatinine only.
[b] Relative Risk (RR) is the ratio of risk for MAKE at Day 30/90 in RMC-035 group divided by ratio of risk for MAKE at Day 30/90 in placebo group (RR<1 indicates decreased risk of MAKE at Day 30/90 in subjects treated with IMP versus placebo). Confidence interval utilizes Greenland and Robins.
[c] Cochran-Mantel-Haenszel estimate with stratification factors of region (North America and Europe) and eGFR at Day -1 (≥60 and <60 mL/min/1·73 m²).

Supplementary Table 7: Summary of Most Frequently Reported (≥10%) Treatment-Emergent Adverse Events

System Organ Class<br>Preferred Term [n (%)]	RMC-035<br>(N=89)	Placebo<br>(N=88)
Any TEAE	76 (85·4)	66 (75·0)
Blood and lymphatic system disorders	33 (37·1)	24 (27·3)
Anemia	18 (20·2)	18 (20·5)
Thrombocytopenia	13 (14·6)	8 (9·1)
Cardiac disorders	41 (46·1)	35 (39·8)
Atrial fibrillation	17 (19·1)	26 (29·5)
Tachycardia	9 (10·1)	0 (0·0)
Gastrointestinal disorders	25 (28·1)	12 (13·6)
Nausea	19 (21·3)	8 (9·1)
General disorders and administration site conditions	40 (44·9)	15 (17·0)
Chills	27 (30·3)	2 (2·3)
Pyrexia	11 (12·4)	2 (2·3)
Injury, poisoning and procedural complications	25 (28·1)	16 (18·2)
Procedural pain	11 (12·4)	9 (10·2)
Metabolism and nutrition disorders	27 (30·3)	30 (34·1)
Hypervolemia	9 (10·1)	13 (14·8)
Psychiatric disorders	18 (20·2)	10 (11·4)
Delirium	9 (10·1)	4 (4·5)
Renal and urinary disorders	18 (20·2)	14 (15·9)
Acute kidney injury	12 (13·5)	11 (12·5)
Respiratory, thoracic and mediastinal disorders	23 (25·8)	23 (26·1)
Pleural effusion	9 (10·1)	12 (13·6)
Vascular disorders	29 (32·6)	13 (14·8)
Hypertension	9 (10·1)	4 (4·5)
Hypotension	10 (11·2)	9 (10·2)

n/N = number of subjects; NA = not applicable; TEAE = treatment-emergent adverse event.

Supplementary Table 8: Summary of Treatment-Emergent Adverse Events Suggestive of Infusion Related Reactions on Days of Treatment

Parameter	RMC-035 (N=89) n (%)	Placebo (N=88) n (%)
Any TEAE suggestive of IRR	32 (36·0)	5 (5·7)
Chills	27 (30·3)	2 (2·3)
Hypertensive crisis	6 (6·7)	0 (0·0)
Infusion related reaction	1 (1·1)	0 (0·0)
Pyrexia	11 (12·4)	2 (2·3)
Tremor	0 (0·0)	1 (1·1)
Strongest relationship to IMP [a]		
Not Related	2 (2·2)	4 (4·5)
Unlikely Related	1 (1·1)	1 (1·1)
Possibly Related	20 (22·5)	0 (0·0)
Probably Related	8 (9·0)	0 (0·0)
Related	1 (1·1)	0 (0·0)
Greatest NCI CTCAE v5.0 intensity [b]		
1 - Mild	9 (10·1)	4 (4·5)
2 - Moderate	16 (18·0)	1 (1·1)
3 - Severe	2 (2·2)	0 (0·0)
4 - Life-threatening	5 (5·6)	0 (0·0)
5 - Death	0 (0·0)	0 (0·0)
TEAE suggestive of IRR leading to discontinuation of study drug	1 (1·1)	0 (0·0)
Serious TEAE suggestive of IRR	5 (5·6)	0 (0·0)
Onset relative to IMP infusion – Any TEAE suggestive of IRR		
Infusion 1	0 (0·0)	0 (0·0)
Infusion 2	2 (2·2)	4 (4·5)
Infusion 3	0 (0·0)	0 (0·0)
Infusion 4	4 (4·5)	0 (0·0)
Infusion 5	21 (23·6)	0 (0·0)

AE = adverse event; IMP = investigational medicinal product; IRR = infusion related reaction; n/N = number of subjects; NCI CTCAE v5.0 = National Cancer Institute Common Terminology for Adverse Events Version 5.0; TEAE = treatment emergent adverse event.

[a] Subjects reporting more than one AE are counted only once in the row of the strongest relationship to IMP among all reported AEs for that subject.

[b] Subjects reporting more than one AE are counted only once in the row of the greatest intensity among all reported AEs for that subject.

Supplementary Table 9: Summary of Changes from Baseline to Day 4 in Safety Laboratory Values

Parameter [unit]	RMC-035 [N=89] n Mean (SD)	Placebo [N=88] n Mean (SD)
**Serum Chemistry**		
Albumin [g/L]	69<br>-11·051 (5·7667)	72<br>-10·281 (4·0738)
Blood urea nitrogen [mmol/L]	76<br>1·959 (4·5865)	80<br>1·627 (3·4829)
C-reactive protein [mg/L]	74<br>153·511 (68·1411)	72<br>168·131 (75·3464)
Calcium [mmol/L]	80<br>-0·265 (0·1628)	76<br>-0·264 (0·1684)
Chloride [mmol/L]	73<br>-0·108 (4·8231)	72<br>0·666 (13·3216)
eGFR [mL/min/1.73 m^2]	84<br>-4·416 (17·4823)	84<br>2·116 (16·3369)
Glucose [mmol/L)	74<br>2·939 (16·5944)	76<br>3·365 (13·4238)
Magnesium [mmol/L]	69<br>0·073 (0·1725)	69<br>0·087 (0·1928)
Phosphate [mmol/L]	72<br>-0·445 (2·5007)	70<br>-0·303 (0·3399)
Potassium [mmol/L]	77<br>-0·138 (0·5735)	76<br>-0·145 (0·6888)
Sodium [mmol/L]	77<br>-0·247 (4·0560)	78<br>-2·385 (6·3045)
Urate [µmol/L]	64<br>-52·504 (112·9860)	61<br>-38·812 (85·7296)
**Haematology**		
Basophils [%]	64<br>-0·221 (0·7998)	58<br>-0·377 (0·4372)
Eosinophils [%]	64<br>-0·999 (3·5845)	58<br>-1·007 (1·9644)
Lymphocytes [%]	65<br>-8·780 (19·9069)	58<br>-10·051 (7·7820)
Monocytes [%]	65<br>-1·326 (2·7413)	58<br>-0·598 (2·4550)
Neutrophils [%]	65<br>12·037 (12·4883)	57<br>7·921 (17·8843)
Erythrocyte mean corpuscular haemoglobin concentration [mmol/L]	84<br>-0·127 (0·5786)	84<br>-0·013 (0·6089)
Erythrocyte mean corpuscular haemoglobin [fmol]	84<br>-0·009 (0·0474)	84<br>0·009 (0·0487)
Erythrocyte mean corpuscular volume [fL]	84<br>-0·018 (2·8770)	84<br>1·436 (8·7526)
Erythocytes [10^{12}/L]	72<br>1·189 (4·2264)	72<br>0·924 (1·4957)
Erythrocytes distribution width [fraction of 1]	84<br>-1·260 (1·3085)	84<br>-1·422 (0·5580)
Haematocrit [L/L]	84<br>-0·563 (4·0635)	84<br>-0·600 (4·3874)
Haemoglobin [g/L]	84<br>-41·464 (19·1906)	84<br>-42·265 (20·2772)
Leukocytes [10^9/L]	84<br>-106·223 (1013·9731)	84<br>3·194 (3·7930)
Platelets [10^9/L]	84<br>-95·690 (56·6120)	84<br>-80·893 (49·7354)
**Liver Function**		
Alanine aminotransferase [µkat/L]	84<br>0·9991 (5·74390)	80<br>0·1635 (1·37768)
Asparatate aminotransferase [µkat/L]	81<br>0·6547 (3·57383)	80<br>0·3112 (1·08491)
Alkaline phosphatase [µkat/L]	73<br>-0·0418 (0·44304)	64<br>-0·1323 (0·38376)
Bilirubin, total [µmol/L]	82<br>4·2881 (19·68835)	79<br>-0·0171 (5·54093)
Bilirubin, direct [µmol/L]	40<br>2·1387 (3·45277)	29<br>1·5276 (3·74794)
Gamma glutamyl transferase [µkat/L]	80<br>0·0594 (0·99820)	76<br>0·0291 (0·35979)

eGFR = estimated glomerular filtration rate; SD = standard deviation

Supplementary Table 10: Summary of Changes from Baseline to Day 4 in Vital Signs

Parameter [unit]	RMC-035 [N=89] n Mean (SD)	Placebo [N=88] n Mean (SD)
Systolic blood pressure [mmHg]	84<br>3·4 (26·81)	85<br>-0·0 (26·18)
Diastolic blood pressure [mmHg]	84<br>1·88 (17·528)	85<br>-0·46 (17·753)
Heart rate [beats/min]	83<br>17·0 (18·98)	84<br>19·5 (15·32)
Temperature [C]	81<br>0·45 (0·740)	83<br>0·38 (0·745)
Respiratory rate [breaths/min]	74<br>2·4 (4·05)	77<br>2·8 (4·80)
Oxygen saturation [%]	82<br>-1·44 (3·594)	82<br>-1·39 (3·269)

SD = standard deviation

Supplementary Figure 1: Study Flow Chart

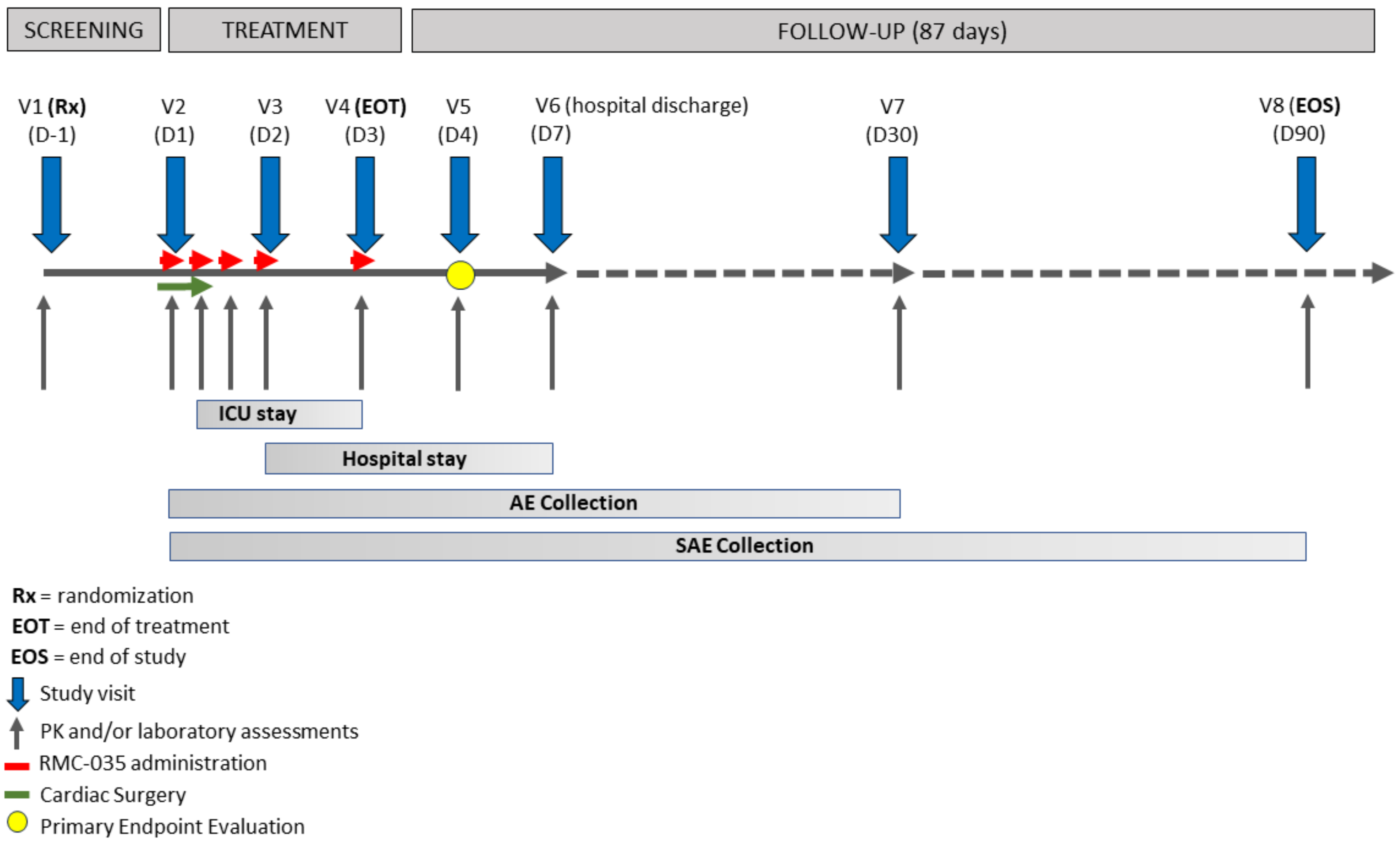

Supplementary Figure 2: Change from Baseline in Serum Creatinine by eGFR Subgroup

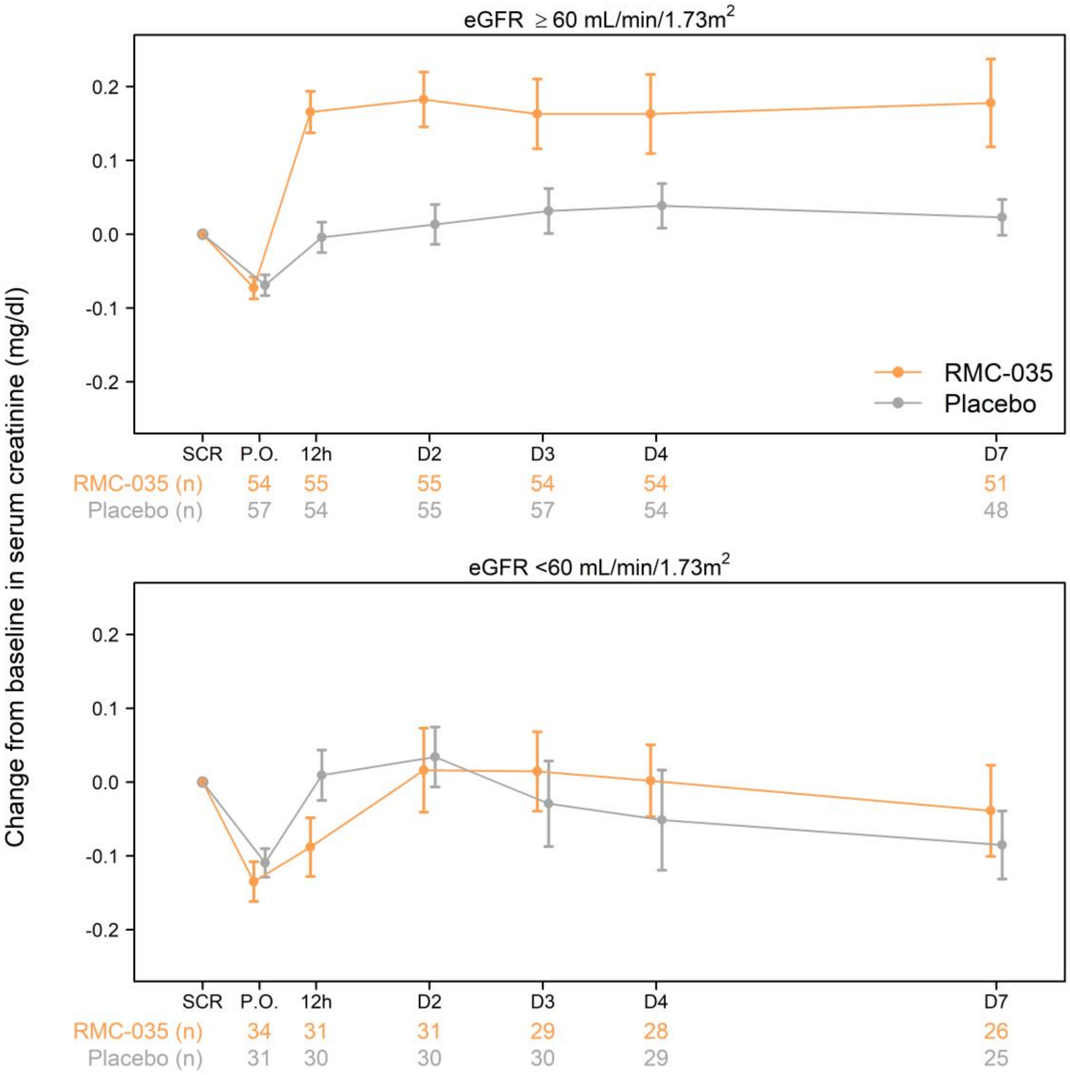

Supplementary Figure 2: Mean SCr changes from baseline at Day 7, 30 and 90 by eGFR subgroups.
Upper panel shows the change from baseline in SCr (mg/dL) at randomisation, pre-operatively, day 7, 30 and 90 of patients with a baseline eGFR ≥ 60mL/min/1.73m2. Lower panel shows the change from baseline in SCr (mg/dL) at randomisation, pre-operatively, day 7, 30 and 90 of patients with a baseline eGFR < 60mL/min/1.73m2.
Orange: RMC-035; Grey: Placebo
Data are shown as mean ±standard error
SCR: screening; P.O.: pre-operatively, D7: Day 7; D30: Day 30; D90: Day 90; n: number of patients;

Supplementary File 2: Statistical Analysis Plan

A Phase 2, Randomized, Placebo-Controlled, Double-Blind, Adaptive, Parallel Group Clinical Study to Evaluate the Efficacy and Safety of RMC-035 in Subjects at High Risk for Acute Kidney Injury Following Open-Chest Cardiac Surgery

Protocol Number:	21-ROS-05
Protocol Version:	3.1
Protocol Date:	08 October 2022

STATISTICAL ANALYSIS PLAN

Version 2.0

A Phase 2, Randomized, Placebo-Controlled, Double-Blind, Adaptive, Parallel Group Clinical Study to Evaluate the Efficacy and Safety of RMC-035 in Subjects at High Risk for Acute Kidney Injury Following Open-Chest Cardiac Surgery

STATISTICAL ANALYSIS PLAN
Version 2.0

Author: [redacted]
Principal Biostatistician
[redacted]

[redacted] Biostatistics Reviewer: [redacted]
Assistant Director, Biostatistics
[redacted]

Sponsor Approval: [redacted]
Study Statistician
Guard Therapeutics International AB

Note that the last signature date is the effective date of the plan.

Table of Contents

LIST OF ABBREVIATIONS 6

CHANGES FROM PROTOCOL 9

CHANGES FROM SAP V1 TO SAP V2 9

1. INTRODUCTION 19

2. OBJECTIVES AND ENDPOINTS 19

2.1 Objectives 19
2.1.1 Primary Objective 19
2.1.2 Key Secondary Objectives 19
2.1.3 Other Secondary Objectives 19
2.1.4 Exploratory Objective 20
2.2 Endpoints 20
2.2.1 Primary Endpoint 20
2.2.2 Key Secondary Endpoints 21
2.2.3 Other Secondary Endpoints 21
2.2.4 Exploratory Endpoints 22

3. INVESTIGATIONAL PLAN 23

3.1 Study Design 23
3.2 Treatment 24
3.2.1 Randomization Scheme and Treatment Arm Assignment 25
3.2.2 Replacement of Subjects 25
3.2.3 Blinding 25
3.2.4 Dosing Schedule 26
3.2.5 Treatment Compliance 26

4. GENERAL CONSIDERATIONS FOR DATA ANALYSIS 26

4.1 Analysis Quality Control Procedures 27
4.2 Analysis Sets 27
4.3 Assessment Windows 27
4.4 Handling of Dropouts or Missing Data 29
4.5 Multiple Comparisons 29
4.6 Data Derivations and Transformations 29
4.6.1 Primary Efficacy Endpoint Considerations 32
4.6.2 Other Efficacy Endpoint Considerations 32

5. STUDY SUBJECTS 32

5.1 Disposition of Subjects 32
5.2 Protocol Deviations 33
5.3 Demographic and Baseline Characteristics 33
5.4 Medical History 33

5.5 Prior and Concomitant Medications 33

6. EFFICACY ANALYSIS 33

6.1 Primary Efficacy Endpoint and Analysis 34
6.1.1 Supplementary Analyses of the Primary Endpoint 34
6.1.2 Sensitivity Analyses of the Primary Endpoint 35
6.2 Key Secondary Efficacy Endpoints and Analyses 35
6.3 Other Secondary Efficacy Endpoints and Analyses 37
6.4 Exploratory Efficacy Endpoints and Analyses 40
6.5 Subgroup Analysis 41

7. SAFETY ANALYSIS 41

7.1 Extent of Exposure 41
7.2 Adverse Events 41
7.2.1 Treatment-emergent Adverse Events 42
7.2.2 Post-treatment Adverse Event 42
7.2.3 Adverse Event Severity 42
7.2.4 Adverse Event Relationship to Study Medication 43
7.2.5 Serious Adverse Events 43
7.2.6 Adverse Events of Special Interest 43
7.2.7 Adverse Event Summaries 44
7.3 Clinical Laboratory Assessments 46
7.4 Vital Signs 46
7.5 ECG 47
7.6 Physical Examination 47

8. SAMPLE SIZE AND POWER CALCULATIONS 47

9. INTERIM ANALYSIS 48

9.1 Conditional Power 49
9.2 Sample Size Readjustment 50
9.3 Alpha Level 50
9.4 Conduct of Unblinded Interim Analysis 50

10. BLINDING AND UNBLINDING 51

10.1 Blinded Statistician 51
10.2 Management of Accidental Unblinding 51
10.3 Unblinding for Analysis Purposes at the End of the Trial 51

11. REFERENCES 51

12. APPENDICES 52

12.1 APPENDIX A: SCHEDULE OF ASSESSMENTS 52
12.2 APPENDIX B: CKD-EPI CALCULATIONS 56
12.3 APPENDIX C: DOSING TABLES 58
12.3.1 Table C1: Volumes of IMP and diluent for dose level 1.3 mg/kg 58

12.3.2 Table C2: Volumes of IMP and diluent for dose level 0.65 mg/kg 61
12.4 APPENDIX D: eCRF DOCUMENTATION 64
12.4.1 Table D1: Risk Factors 64
12.4.2 Table D2: Inclusion/Exclusion Criteria 64
12.5 APPENDIX E: DERIVATION OF URINE OUTPUT 69
12.6 APPENDIX F: CONDITIONAL POWER ANALYSIS 73

LIST OF ABBREVIATIONS

Abbreviation or Specialist Term	Explanation
8-OHdG	8-hydroxy-2'-deoxyguanosine
A1M	Alpha-1-microglobulin
ADA	Anti-drug antibodies
ADL	Activities of Daily Living
AE	Adverse event
AKI	Acute kidney injury
ATC	Anatomical, Therapeutic, and Chemical
AUC	Area under the curve
BPM	Beats per minute
CABG	Coronary artery bypass grafting
CCL-14	Chemokine ligand 14
CDF	Cumulative Distribution Function
CI	Confidence interval
CKD-EPI	Chronic Kidney Disease Epidemiology Collaboration
C_{max}	Maximum (peak) concentration observed
CP	Conditional power
CPB	Cardiopulmonary bypass
CRF	Case report form
CRO	Contract research organization
CTCAE	Common Terminology Criteria for Adverse Events
cTnI	Cardiac Troponin I
cTnT	Cardiac Troponin T
ECG	Electrocardiogram
eGFR	Estimated glomerular filtration rate
EOS	End-of-Study
EOT	End-of-Treatment
EQ-5D-5L	European Quality of Life 5 Domain 5-Level Score
GCP	Good Clinical Practice
GCV	Geometric coefficient of variation

**Abbreviation or Specialist Term**	**Explanation**
GM	Geometric mean
GSD	Geometric standard deviation
h	Hour(s)
IA	Interim analysis
IBW	Ideal Body Weight
ICF	Informed Consent Form
ICH	International Conference on Harmonization
ICU	Intensive care unit
IEC	Independent Ethics Committee
IGFBP7	Insulin-like growth binding factor 7
IL-18	Interleukin-18
IMP	Investigational medicinal product
IRB	Institutional Review Board
IRR	Infusion related reaction
ISR	Infusion site reaction
ITT	Intent to Treat
IV	Intravenous
KDIGO	Kidney Disease Improving Global Outcomes
KIM-1	Kidney injury molecule 1
LFABP	Liver fatty acid binding protein
MAKE	Major adverse kidney events
MAR	Missing at random
MedDRA	Medical Dictionary for Regulatory Activities
MI	Multiple imputation
mITT	Modified Intent to Treat
MMRM	Mixed model for repeated measurements
MOS	Medical outcomes study
NGAL	Neutrophil gelatinase-associated lipocalin
NSAID	Non-steroidal anti-inflammatory drugs
NT-pro-BNP	N-terminal-pro-b-type natriuretic peptide
PK	Pharmacokinetic

**Abbreviation or Specialist Term**	**Explanation**
PP	Per Protocol
PRO	Patient Reported Outcome
PT	Preferred term
PTAE	Post-treatment adverse event
Q1	First quartile
Q2	Second quartile (median)
Q3	Third quartile
RRT	Renal replacement therapy
SAE	Serious adverse event
SAF	Safety analysis set
SAP	Statistical Analysis Plan
SCr	Serum creatinine
SD	Standard deviation
SF-36	Medical Outcomes Study 36-Item Short Form Survey Instrument
SOC	System organ class
SpO2	Oxygen saturation
SUSAR	Suspected Unexpected Serious Adverse Reaction
TEAE	Treatment emergent adverse event
TESAE	Treatment emergent serious adverse event
TIMP2	Tissue inhibitor of metalloproteinase 2
UACR	Urine albumin-to-creatinine ratio
UPCR	Urine protein-to-creatinine ratio
UO	Urine output
VAS	Visual analogue scale
WHO	World Health Organization

CHANGES FROM PROTOCOL

Protocol Verbiage	Modification	Rationale
The AUC will be calculated using the **trapezoidal rule** with the **actual** times the measurements are taken.	The AUC will be calculated using the **right Riemann sum** with the **dates/times the measurements are planned**.	Updated to be consistent with planned analysis.
Section 6.3<br><br>Endpoint: SCr, cystatin C, and eGFR at 12, 24, 48, and 72 hours, respectively, and at Day 7/discharge, Day 30, and Day 90<br><br>Endpoint: Change from baseline, up to Day 7/discharge, of peak SCr and cystatin C	See changes outlined below in "Changes from SAP V1 to V2"	Updated planned analysis.

CHANGES FROM SAP V1 TO SAP V2

Endpoint or Variable	SAP Verbiage	Modification	Rationale
Introduction<br><br>(Section 1)	Original Verbiage:<br><br>This document details the statistical methods planned to perform the interim and final analyses of the study. An interim sample-size re-assessment will be performed, during which subject enrollment will continue. Sample size may be increased to a maximum of 348 subjects dosed, but will not be decreased.	Verbiage changed to:<br><br>This document details the statistical methods planned to perform the final analyses of the study. An interim sample-size re-assessment was performed and it was determined to stop subject enrollment. Updates to SAP v2 reflect any changes from the original planned SAP v1 (e.g., data might be provided as opposed to generating a statistical analysis).	Updated after determination to stop study enrollment.

Endpoint or Variable	SAP Verbiage	Modification	Rationale
Other Secondary Endpoints<br><br>(Section 2.2.3)	Original Verbiage:<br><br>MAKE at Day 30 and Day 90, defined as death, any dialysis, or ≥25% reduction of estimated glomerular filtration rate (eGFR) compared to baseline using the Chronic Kidney Disease-Epidemiology Collaboration (CKD-EPI) equations (**with either SCr, cystatin C, or both**)	Verbiage changed to:<br><br>MAKE at Day 30 and Day 90, defined as death, any dialysis, or ≥25% reduction of estimated glomerular filtration rate (eGFR) compared to baseline using the Chronic Kidney Disease-Epidemiology Collaboration (CKD-EPI) equations (**with SCr, cystatin C, and both**).	Updated for clarification and to be consistent with planned analysis.
General Considerations for Data Analysis<br><br>(Section 4)	Original Verbiage:<br><br>[redacted] will perform all efficacy and safety statistical analyses described in this SAP. [redacted] will manage the pharmacokinetic (PK) portion of the study. Descriptive statistics of drug concentrations over time will be provided.	Verbiage changed to:<br><br>[redacted] will perform all efficacy and safety statistical analyses described in this SAP. [redacted] will manage the bioanalysis of pharmacokinetic (PK) measurements of the study and descriptive statistics of drug concentrations over time will be performed and provided by [redacted]	Updated for clarification and to be consistent with planned analysis.
Table 1<br><br>(Section 4.3)		Addition of Visit Day 1 (Pre-Dose) and analysis visit windows.	Updated for thorough listing of data.
Actual Dose<br><br>(Section 4.6)		Updated definition of Actual Dose to encompass placebo as well as IMP.	Updated for clarity in presentation of data.

Endpoint or Variable	SAP Verbiage	Modification	Rationale
Demographic and Baseline Characteristics<br><br>(Section 5.3)		Added verbiage:<br><br>Surgical details for the cardiac surgery include types and location of surgery, surgical duration, CPB details, and vital assessments will be summarized and listed as well.	Updated to be consistent with planned analysis.
Analysis for Other Secondary Endpoints: SCr, cystatin C, and eGFR at 12, 24, 48, and 72 hours, respectively, and at Day 7/discharge, Day 30, and Day 90<br><br>(Section 6.3)	For SCr, cystatin C, and eGFR, robust regression will be utilized to determine the between-arm difference at each visit.<br><br>The existing 3-phase multiple imputation approach detailed above will be re-used for this analysis. In the analysis step, AUC (dependent variable) will be replaced by either VAL1, VAL2, VAL3, VAL4, VAL5, VAL6, or VAL7 representing the missing-data-free log-transformed SCr or cystatin C values at Day 1 (12 hour), Day 2 (24 hour), Day 3 (48 hour), Day 4 (72 hour), Day 7, Day 30, and Day 90. Imputed eGFR values will be calculated from the imputed SCr data, as well as the imputed cystatin C data.<br><br>Between-arms differences of the Robust regression estimates will be averaged over imputations using Rubin's rules. Between-arms differences of the Robust regression estimates will be transformed back to the original scale using $100\times(e^{(statistic)}-1)$ and reported as geometric mean percent changes (RMC-035 vs. placebo). The estimate of the geometric mean percent change from baseline per arm, 90% confidence interval (CI), and p-value will be reported.	For SCr, cystatin C, and eGFR, descriptive statistics will be produced by analysis visit on the raw untransformed data. eGFR values will be calculated from the raw untransformed SCr data and cystatin C data. This summary will include all analysis visits including the screening and Day 1 (Pre-Operative) visit. Columns for actual values, changes from baseline, and percent change from baseline (calculated as 100*(change/baseline)) will be included. Changes from baseline will be reported for all visits except screening.<br><br>For SCr, cystatin C, and eGFR, robust regression will be utilized to determine the between-arm difference at each visit. The existing 3-phase multiple imputation approach detailed above will be re-used for this analysis. Imputed eGFR values will be calculated from the imputed SCr data, as well as the imputed cystatin C data. After imputation, all observed and imputed log-transformed SCr,	Updated planned analysis.

Endpoint or Variable	SAP Verbiage	Modification	Rationale
		cystatin C, and eGFR values will be transformed back to the original scale. In the analysis step, AUC (dependent variable) will be replaced by either VAL1, VAL2, VAL3, VAL4, VAL5, VAL6, or VAL7 representing the missing-data-free SCr, cystatin C, or eGFR values at Day 1 (12 hour), Day 2 (24 hour), Day 3 (48 hour), Day 4 (72 hour), Day 7, Day 30, and Day 90. The untransformed baseline values will be subtracted from the resulting values in order to obtain, for each subject, absolute changes from baseline to each of the 7 aforementioned timepoints.<br><br>The analysis phase will be therefore slightly different from that done for AUC in the sense that the model will be applied to the absolute change from baseline. The untransformed baseline values will be used as an independent variable. Between-arms differences of the Robust regression estimates will be averaged over imputations using Rubin's rules. The estimated mean change from baseline per arm and respective 90% CI and p-value will be calculated. Additionally, the estimated between-arm difference will be calculated, along with the respective 90% CI and p-value.	

Endpoint or Variable	SAP Verbiage	Modification	Rationale
		A sensitivity analysis will be performed using a mixed model for repeated measurements (MMRM) separately for each of the untransformed SCr, cystatin C, and eGFR values. The (untransformed) change from baseline values for Day 1 (12 hour), Day 2 (24 hour), Day 3 (48 hour), Day 4 (72 hour), Day 7/Discharge, Day 30 & Day 90 will be modelled in the response vector. A 14-level visit-by-treatment class variable and a 7-level visit-by-screening and visit-by-pre-operation covariate will be included. In addition, region will be used as a fixed factor. For the sensitivity analysis only, subjects without a screening value will use the Day 1 pre-operative value for screening. Additionally for the sensitivity analysis only, subjects without a Day 1 pre-operative value will use the screening value for Day 1 pre-operative. The estimated mean change from baseline per arm and respective 90% CI and p-value will be calculated. Additionally, the estimated between-arm difference will be calculated, along with the respective 90% CI and p-value.	

Endpoint or Variable	SAP Verbiage	Modification	Rationale
Analysis for Other Secondary Endpoints: Change from baseline, up to Day 7/discharge, of peak SCr and cystatin C<br><br>(Section 6.3)	The results of the combined analyses of imputations completed with PROC MIANALZE above will be utilized for this analysis.	The raw untransformed data will be utilized for this analysis. The dependent variable is peak change from baseline (peak value-baseline value). The untransformed baseline values will be used as an independent variable, as well as region. The estimated between-arm difference will be calculated, along with the respective 90% CI and p-value.	Updated planned analysis.
Analysis for Other Secondary Endpoint: MAKE by Day 30 and 90<br><br>(Section 6.3)	• Any dialysis **BY** Day X; or<br>• A ≥25% reduction of estimated glomerular filtration rate (eGFR) **BY** Day X compared to baseline using the Chronic Kidney Disease-Epidemiology Collaboration (CKD-EPI) equations	• Any dialysis **THROUGH** Day X; or<br>• A ≥25% reduction of estimated glomerular filtration rate (eGFR) **ON** Day X compared to baseline using the Chronic Kidney Disease-Epidemiology Collaboration (CKD-EPI) equations	Updated for clarification and to be consistent with planned analysis.
Analysis for Other Secondary Endpoint: MAKE by Day 30 and 90<br><br>(Section 6.3)		Added verbiage:<br>A sensitivity analysis utilizing the alternative definition of MAKE X will be performed as described above, where the alternative definition utilizes cystatin C only. A second sensitivity analysis utilizing the alternative definition of MAKE X will be performed as described above, where the alternative definition utilizes SCr and cystatin C. See Appendix B for the three calculations.	Updated for clarification and to be consistent with planned analysis.

Endpoint or Variable	SAP Verbiage	Modification	Rationale
Analysis for Other Secondary Endpoint: Post-baseline changes in urine albumin to creatinine ratio (UACR) and urine protein to creatinine ratio (UPCR) at Day 4, Day 30, and Day 90<br><br>(Section 6.3)	Original SAP Verbiage:<br><br>This endpoint will be analyzed as described in Section 6.2 for the key secondary AUC endpoint.	Removed original SAP verbiage and replaced with:<br><br>The data will be reported separately.	Updated after determination to stop study enrollment and to discontinue collection of UACR/UPCR values.
Exploratory Endpoint: Pharmacokinetics of RMC-035 in plasma (AUC and C_{max})<br><br>(Section 6.3)	Original Verbiage:<br><br>[redacted] will manage the pharmacokinetic portion of the study. Descriptive statistics of drug concentrations over time will be provided.	Verbiage changed to:<br><br>[redacted] will conduct bioanalysis of PK samples with PK parameters and reporting carried out by CTC. Descriptive statistics of drug concentrations over time will be provided by dose group and by renal function at baseline.	Updated to be consistent with planned analysis.
Appendix B		Addition of Appendix B: CKD-EPI CALCULATIONS	Updated for clarity.
Appendix C		Moved Appendix B (DOSING TABLES) to Appendix C	Updated for inclusion of other appendices.
Appendix D		Addition of Appendix C: eCRF Documentation. Appendix C includes a table for Risk Factors and Inclusion/Exclusion criteria	Updated for clarity.

Endpoint or Variable	SAP Verbiage	Modification	Rationale
Appendix E		Moved Appendix C (Derivation of Urine Output) to Appendix E	Updated for inclusion of other appendices
Appendix F		Moved Appendix D (Sample Size Assessment Utilizing Primary Endpoint (AKI)) to Appendix F	Updated for inclusion of other appendices
Exploratory Endpoint: Presence and titers of ADA at Day 1 (pre-surgery), Day 30, and Day 90<br><br>(Section 6.3)	Original SAP Verbiage:<br><br>Summary tables will be provided, including a shift table for presence of ADA between Visit 2 (Day 1/pre-dose) and Visits 7 (Day 30) and 8 (EOS). ADA samples obtained beyond the protocol specified time window for Visits 7 and 8 will be accepted. For ADA evaluation, presence of ADA at a visit will be defined as follows:<br><br>Presence of ADA at a visit is negative if:<br><br>• Initial result is reported as negative, or<br>• Initial result is reported as positive and confirmatory result is reported as negative<br><br>Presence of ADA at a visit is positive if:<br><br>• Both initial and confirmatory result is reported as positive<br><br>If the confirmatory result is missing, presence of ASA will be identical to the initial reported result. Otherwise, presence of ASA will not be evaluable.	Removed original SAP verbiage and replaced with:<br><br>Presence and titers of ADA and ADA characterization will be reported separately	Updated after determination to stop study enrollment.
Exploratory Endpoint: Index ICU stay (in Days)	Original SAP Verbiage:	Removed original SAP verbiage and replaced with:<br><br>Data will be presented separately.	Updated after determination to stop study enrollment.

Endpoint or Variable	SAP Verbiage	Modification	Rationale
(Section 6.3)	Descriptive statistics (n, mean, standard deviation, Q1, median, Q3, minimum, maximum) will be displayed by treatment group.		
Exploratory Endpoint: Index hospital stay (in Days)<br><br>(Section 6.3)	Original SAP Verbiage:<br><br>Descriptive statistics (n, mean, standard deviation, Q1, median, Q3, minimum, maximum) will be displayed by treatment group.	Removed original SAP verbiage and replaced with:<br><br>Data will be presented separately.	Updated after determination to stop study enrollment.
Exploratory Endpoint: Nature of subject discharge facility<br><br>(Section 6.3)	Original SAP Verbiage:<br><br>Descriptive statistics (n, mean, standard deviation, Q1, median, Q3, minimum, maximum) will be displayed by treatment group.	Removed original SAP verbiage and replaced with:<br><br>Data will be presented separately.	Updated after determination to stop study enrollment.
Exploratory Endpoint: Change from baseline to Day 90 for Medical Outcomes Study (MOS) 36-Item Short Form Survey Instrument (SF-36)<br><br>(Section 6.3)	Original SAP Verbiage:<br><br>SF-36 includes one multi-item scale that assesses eight health concepts with two summary measures and is an indicator of overall health status. Lower scores indicate more disability and higher scores indicate less disability.<br><br>Total scores (by eight health concepts, two summary measures, and overall) will be summarized by treatment group and analysis visit. Actual values and changes from baseline will be tabulated separately. All total scores and sub-scale scores will be listed.	Removed original SAP verbiage and replaced with:<br><br>These parameters will be reported separately.	Updated after determination to stop study enrollment.
Exploratory Endpoint: Change from baseline to Day 90 for European Quality of Life 5 Domain 5-Level Score (EQ-5D-5L)	EQ-5D-5L is a health-related quality of life measurement and consists of five dimensions, and each dimension has five response levels.<br><br>Descriptive statistics (frequency and proportions) by domain (5 questions), and treatment group will be	Removed original SAP verbiage and replaced with:<br><br>These parameters will be reported separately.	Updated after determination to stop study enrollment.

Endpoint or Variable	SAP Verbiage	Modification	Rationale
(Section 6.3)	displayed, as well as a shift table. All responses will be listed.		
Exploratory Endpoint: The European Quality Visual Analog Scale (VAS)<br><br>(Section 6.3)	Original SAP Verbiage:<br><br>VAS is a quantitative measure of health outcome that reflect the subject's own judgement. A number between 0-100 is selected, where 100 is "the best health you can imagine" and 0 is "the worst health you can imagine".<br><br>For the 0-100 VAS, descriptive statistics (n, mean, standard deviation, Q1, median, Q3, minimum, maximum) will be displayed by treatment group for actual value and change from baseline. Scores will be listed.	Removed original SAP verbiage and replaced with:<br><br>These parameters will be reported separately.	Updated after determination to stop study enrollment.
Subgroup Analysis<br><br>(Section 6.5)		Removed verbiage:<br><br>If at least 20 subjects have a history of diabetes, the history of diabetes (yes/no) will be included in the subgroup analysis as well.	Updated to be consistent with planned analysis.

1. INTRODUCTION

Acute Kidney Injury (AKI) is a clinical syndrome characterized by rapid loss of renal function and is associated with increased morbidity and short and long-term mortality. This study is being conducted to provide proof-of-concept efficacy data and provide targeted protection against oxidative stress and heme toxicity in this patient population, with the aim to prevent or ameliorate post-surgery renal injury.

This statistical analysis plan (SAP) is based on Protocol 21-ROS-05, titled "A Phase 2, Randomized, Placebo-Controlled, Double-Blind, Adaptive, Parallel Group Clinical Study to Evaluate the Efficacy and Safety of RMC-035 in Subjects at High Risk for Acute Kidney Injury Following Open-Chest Cardiac Surgery". See the study protocol for full details.

This document details the statistical methods planned to perform the final analyses of the study. An interim sample-size re-assessment was performed and it was determined to stop subject enrollment. Updates to SAP v2 reflect any changes from the original planned SAP v1 (e.g., data might be provided as opposed to generating a statistical analysis).

2. OBJECTIVES AND ENDPOINTS

2.1 Objectives

2.1.1 Primary Objective

The primary objectives of this study are:

- To evaluate the efficacy of RMC-035 for prevention of AKI (Kidney Disease Improving Global Outcomes [KDIGO] definition) in subjects undergoing coronary artery by-pass graft (CABG) and/or valve surgery and/or aorta surgery with additional risk factors for developing cardiac surgery associated AKI
- To evaluate the safety and tolerability of RMC-035

2.1.2 Key Secondary Objectives

The key secondary objectives of this study are:

- To evaluate RMC-035 for the prevention of post-operative decline (within 72 hours) in renal function
- To evaluate RMC-035 for the reduction of post-operative AKI duration

2.1.3 Other Secondary Objectives

The other secondary objectives of this study are:

- To evaluate RMC-035 for preserving post-surgery renal function up to Day 90
- To evaluate RMC-035 for the prevention of post-operative dialysis up to Day 90

- To evaluate RMC-035 for the prevention of major adverse kidney events (MAKE) at Days 30 and 90, respectively
- To further evaluate RMC-035 for the
    - Prevention of AKI within 72 hours (based on cystatin C and/or Urine Output [UO])
    - Persistence and severity of AKI within 72 hours (based on serum creatinine [SCr] and/or UO or cystatin C and/or UO)
    - Prevention, persistence, and severity of AKI within 7 days (based on SCr and/or UO or cystatin C and/or UO)
- To evaluate RMC-035 for reducing post-operative albuminuria and proteinuria up to Day 90
- To evaluate the pharmacokinetics of RMC-035
- Identification and characterization of anti-drug-antibodies (ADA) developed after intravenous administration of RMC-035

2.1.4 Exploratory Objective

The exploratory objectives of this study are:

- To evaluate post-baseline changes in kidney and cardiac biomarkers
- To evaluate changes in immunologic biomarkers
- To evaluate the length of post-operative stay in Intensive Care Unit (ICU) and overall hospitalization time
- To evaluate health-related Quality of Life

2.2 Endpoints

2.2.1 Primary Endpoint

The primary efficacy endpoint is:

- AKI within 72 hours after first dose of IMP based on SCr and/or UO (AKI of any stage/severity according to KDIGO definition), where severity of AKI defined as the following:
    - Stage 1: SCr 1.5 to 1.9 times baseline within 7 days, OR ≥0.3 mg/dL (≥26.5 µmol/L) OR urine output <0.5 mL/kg/h for 6 to <12 hours
    - Stage 2: SCr 2.0-2.9 times baseline within 7 days OR urine output <0.5 mL/kg/h for ≥12 hours
    - Stage 3: SCr 3.0 times baseline within 7 days, OR increase in SCr 4.0 mg/dL (≥353.6 µmol/L), OR initiation of renal replacement therapy OR urine output <0.3 mL/kg/h for ≥24 hours OR anuria for ≥12 hours

The primary safety endpoint is:

- Nature, frequency, and severity of treatment-emergent adverse events (TEAEs)

2.2.2 Key Secondary Endpoints

The key secondary endpoints of the study are:

- Time-corrected area under the curve (AUC) of SCr for Day 1 to Day 4 (72 hours after first dose of IMP)
- Duration of AKI defined as the number of days meeting the definition of AKI (KDIGO definition) starting within 72 hours after first dose of IMP until resolution

2.2.3 Other Secondary Endpoints

The other secondary endpoints of the study are:

- Post-baseline changes in renal function
    - SCr, cystatin C, and eGFR (both calculated using SCr and cystatin C) at 12, 24, 48, and 72 hours, respectively, and at Day 7/discharge, Day 30 and Day 90
    - Change from baseline, up to Day 7/discharge, of peak SCr and cystatin C
    - Time-corrected AUC of Cystatin C for Day 1 to Day 4 (72 hours after first dose of IMP)
- Need for renal replacement therapy
    - Dialysis treatment (for any reason) within 72 hours and within 7 days after end of surgery
    - Dialysis-free days from end of surgery to Day 30 and Day 90, respectively
- MAKE at Day 30 and Day 90, defined as death, any dialysis, or ≥25% reduction of estimated glomerular filtration rate (eGFR) compared to baseline using the Chronic Kidney Disease-Epidemiology Collaboration (CKD-EPI) equations (with SCr, cystatin C, and both)
- AKI Characteristics
    - AKI within 72 hours after first dose of IMP based on cystatin C and/or UO (AKI of any stage/severity defined as cystatin C ≥1.5 baseline, OR UO <0.5 mL/kg/h for ≥6 hours)
    - AKI within 7 days after first dose of IMP (based on SCr and/or UO criteria, or cystatin C and/or UO criteria)
    - AKI persistence, defined as an AKI (KDIGO definition) developing within 72 hours after first dose of IMP and with a duration of ≥72 hours. Persistence will also be assessed per AKI severity stage*
    - AKI severity stage* within 72 hours and within 7 days after first dose of IMP
        - *Severity of AKI defined as the following:
            - Stage 1: SCr 1.5 to 1.9 times baseline within 7 days, OR ≥0.3 mg/dL (≥26.5 µmol/L) OR urine output <0.5 mL/kg/h for 6 to <12 hours
            - Stage 2: SCr 2.0-2.9 times baseline within 7 days OR urine output <0.5 mL/kg/h for ≥12 hours

  - Stage 3: SCr 3.0 times baseline within 7 days, OR increase in SCr 4.0 mg/dL (≥353.6 µmol/L), OR initiation of renal replacement therapy OR urine output <0.3 mL/kg/h for ≥24 hours OR anuria for ≥12 hours

- Post-baseline changes in urine albumin to creatinine ratio (UACR) and urine protein to creatinine ratio (UPCR) at Day 4, Day 30, and Day 90
- Pharmacokinetics of RMC-035 in plasma (AUC and C_{max})
- Presence and titers of ADA at Day 1 (pre-surgery), Day 30, and Day 90
- Characteristics of ADA developed at Day 30 and Day 90 with regards to isotype, neutralizing capacity, and cross-reactivity with endogenous alpha-1-microglobulin (A1M)

2.2.4 Exploratory Endpoints

The exploratory endpoints for post-baseline changes in kidney and cardiac biomarkers are:

- Kidney Biomarkers: Urine kidney injury molecule 1 (KIM-1), neutrophil gelatinase-associated lipocalin (NGAL), tissue inhibitor of metalloproteinase 2 (TIMP2), insulin-like growth binding factor protein 7 (IGFBP7), chemokine ligand 14 (CCL-14), interleukin-18 (IL-18), liver fatty acid binding protein (LFABP) and 8-hydroxy-2'-deoxyguanosine (8-OHdG)
- Cardiac Biomarkers: Plasma N-terminal-pro-hormone BNP (NT-pro-BNP) and cardiac troponin I and T (cTnI, cTnT)

The exploratory endpoint for changes in immunologic biomarkers is:

- Immunologic biomarkers to explore the background and mechanism of potential IRRs including, but not limited to, markers of complement activation, cytokine release, and mast cell activation.

The exploratory endpoints for hospitalization time and discharge facility are:

- Length of index ICU stay and index hospital stay
  - Index ICU stay (in Days) defined as the duration of stay in the ICU immediately following surgery or recovery room post-surgery until ICU discharge
  - Index hospital stay (in Days) is defined as the duration of stay in the hospital from the day of surgery to hospital discharge for the index surgery
- Nature of subject discharge facility (e.g., home, skilled nursing facility, rehabilitation center)

The exploratory endpoints for health-related Quality of Life assessments are:

- Change from baseline to Day 90 in the following Patient Reported Outcomes (PROs):
  - Medical Outcomes Study (MOS) 36-Item Short Form Survey Instrument (SF-36)
  - European Quality of Life 5 Domain 5-Level Score (EQ-5D-5L)

3. INVESTIGATIONAL PLAN

3.1 Study Design

This is a Phase 2, randomized, double-blind, adaptive, parallel group clinical study that will evaluate RMC-035 compared to placebo in approximately 268 subjects at high risk for AKI following open-chest cardiac surgery (based on predefined risk factors). An interim sample-size re-assessment will be performed, during which subject enrollment will continue. The study consists of 8 visits for each subject with four study periods:

- Screening Period: 30 days (Day-30 to -1)
- Treatment Period: 3 days (Day 1-3)
- Follow-Up Period: 27 days (Day 4-30)
- Extended Follow-Up Period: 60 days (Day 31-90)

A short overview for study schedule is in Figure 1.

Figure 1: Study Flow Chart

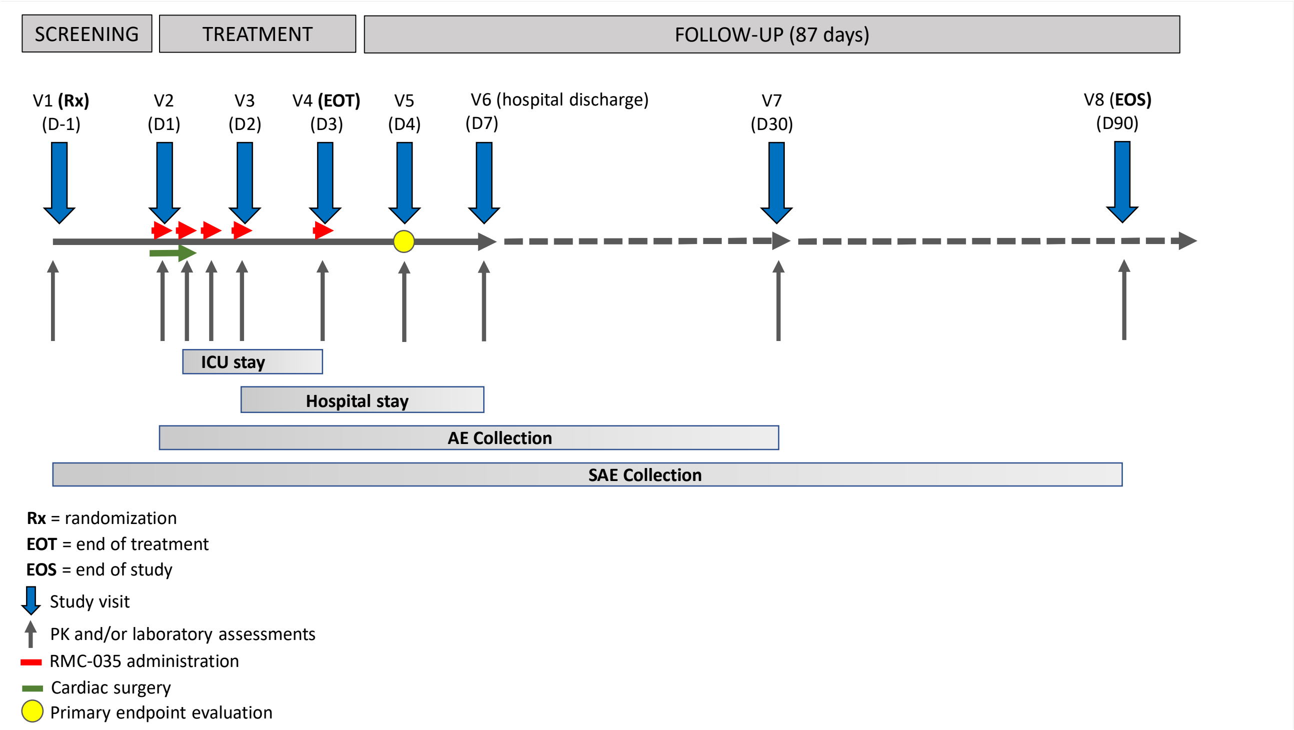

Eligible subjects who comply with all inclusion criteria and none of the exclusion criteria (refer to study protocol for details) will be randomized on Day -1, Visit 1 to receive either RMC-035 or placebo in a 1:1 randomization ratio. Both region (North America and Europe) and Day -1 eGFR calculated using local laboratory results (≥60 and <60 mL/min/1.73m^2) will be used as stratification factors to ensure a balanced randomization within these groups.

Surgery will occur on Day 1 (Visit 2). The first administration of IMP will be given during surgery, to be started approximately 10 minutes before the expected initiation of cardiopulmonary bypass (CPB), as an IV infusion. The start time of the first infusion is time 0, the reference point for further dose administrations and protocol-mandated assessments. In total, 5 doses of RMC-035 or matching placebo will be administered during the treatment period. Administration of subsequent IV infusions start at 6, 12, 24 and 48 hours, respectively, after the start of the first dose administration.

IMP will be administered per the schedule below:

- The first (0h) and second (6h) doses are administered as IV infusions over 60 minutes
- The third (12h), fourth (24h) and fifth (48h) doses are administered as IV infusions over 30 minutes

At a minimum, all subjects will remain hospitalized for the first 72 hours post-surgery (until completion of Visit 5). Subjects will initially be followed up for one month after surgery until the next study visit (Day 30, Visit 7). A final study visit will be conducted at 3 months (Day 90) after surgery (Visit 8, End of Study [EOS]). Upon completion of all EOS visit procedures, subjects will have completed the study.

See Appendix A for the Schedule of Assessments.

3.2 Treatment

The IMPs for this study are RMC-035 or matching placebo. RMC-035 6.0 mg/mL Concentrate for Solution for Infusion (RMC-035) contains the pharmacologically active protein RMC-035, a recombinant variant of endogenous human A1M. The formulation of the matching placebo is identical to RMC-035 except that it contains no active ingredient.

Dose levels of IMP will be predefined based on renal function (eGFR calculated using the CKD-EPI equation with SCr) at screening (Day -1):

- Subjects with eGFR ≥60 mL/min/1.73m^2 will receive 1.3 mg/kg (per dose) for the first and second dose, followed by 0.65 mg/kg (per dose) for the third, fourth, and fifth dose
- Subjects with eGFR >30 and <60 mL/min/1.73m^2 will receive 0.65 mg/kg (per dose) for all 5 doses

The start time of the first infusion of IMP is time 0, the reference point for further dose administrations. In total, 5 doses of IMP or matching placebo will be administered during the treatment period. Administration of subsequent IV infusions will start at 6, 12, 24, and 48 hours, respectively, after the start of the first dose administration.

The first (0h) and second (6h) dose are administered as IV infusions over 60 minutes. The third (12h), fourth (24h) and fifth (48h) doses are administered as IV infusions over 30 minutes.

3.2.1 Randomization Scheme and Treatment Arm Assignment

Each subject who enters the screening period, which starts when the subject signs the ICF, will receive a unique subject identification number. This number will be used to identify the subject throughout the study and must be used on all study documentation related to the subject.

Eligible subjects will be randomized prior to surgery to receive either RMC-035 or placebo in a 1:1 randomization ratio using a centralized randomization process. Both region (North America and Europe) and Day -1 eGFR calculated using local laboratory results (≥60 and <60 mL/min/1.73m^2) will be used as stratification factors to ensure a balanced randomization within these groups.

Approximately 30 centers located in North America and Europe will participate in this study. During the conduct of the study, additional regions, countries, or sites may be added, as necessary.

A central randomization system, Medidata Rave Randomization & Trial Supply Management (RTSM)®, will be used for this study.

3.2.2 Replacement of Subjects

Subjects who are randomized but do not receive the first dose of IMP may be replaced; these subjects (randomized but not dosed) will be followed for safety and for the following endpoints:

- AKI within 72 hours after the first dose of IMP (Visit 4, EOT)
- Time-corrected AUC of SCr for Day 1 to Day 4 (72 hours after first dose of IMP)
- MAKE at Day 30 (Visit 7) and Day 90 (Visit 8, EOS)

Additional assessments are not required to be collected.

Subjects included to replace others will be randomized (i.e., may not be automatically allocated to the same arm to the replaced subject).

Any subject receiving at minimum the first dose of IMP will not be replaced.

3.2.3 Blinding

RMC-035 Concentrate for Solution for Infusion and placebo are filled in glass vials and are visually identical. Vials of each kind will be labelled and packaged separated from each other in a non-blinded way. Blinding will be assured at the time of labelling of the infusion syringe.

The Investigator, study personnel, and subject will be blinded to the identity of the IMP (RMC-035 or placebo). The investigational pharmacist will be responsible for the preparation of IMP for each subject and will be unblinded to the randomization assignment.

The treatment code for a given randomized subject will be provided in the event of a medical emergency requiring knowledge of the treatment assigned to the subject. The time, date, subject number and reason for obtaining any of these codes, and therefore breaking the blind, must be documented in the study file. The treatment code can only be requested by the Investigator or other persons designated as sub-investigators. No subjects or other study personnel will be made aware of the treatment given unless a medical emergency necessitates such disclosure.

Except for determining whether a Suspected Unexpected Serious Adverse Reaction (SUSAR) requires expedited reporting to regulatory authorities, unblinding of the study medication should only be considered for subject safety or when critical therapeutic decisions are contingent upon knowing the blinded IMP assignment. Any unblinding by the investigational staff must be reported immediately to the Sponsor or designee and must include an explanation of why the study medication was unblinded. If possible, the Sponsor and/or designee should be contacted prior to unblinding of the study medication.

3.2.4 Dosing Schedule

Surgery will occur on Day 1 (Visit 2). The first administration of Investigational Medicinal Product (IMP: RMC-035 or Placebo) will be given during surgery, approximately 10 minutes before the expected initiation of cardiopulmonary bypass (CPB), as an intravenous (IV) infusion. The start time of the first infusion is time 0, the reference point for further dose administrations and protocol-mandated assessments. In total, 5 doses of IMP will be administered during the treatment period. Administration of subsequent IV infusions start at 6, 12, 24, and 48 hours, respectively, after time 0 (start of the first dose).

Administration of RMC-035 or placebo:

- The first (0h) and second (6h) doses are administered as IV infusions over 60 minutes
- The third (12h), fourth (24h), and fifth (48h) doses are administered as IV infusions over 30 minutes

Due to the pharmacokinetics of RMC-035, the predefined dose levels (prior to surgery), are based on renal function (eGFR by CKD-EPI equation with SCr) at screening (Day -1, Visit 1):

- Subjects with eGFR ≥60 mL/min/1.73m^2 will receive 1.3 mg/kg (per dose) for the first and second dose, followed by 0.65 mg/kg (per dose) for the third, fourth, and fifth dose
- Subjects with eGFR >30 and <60 mL/min/1.73m^2 will receive 0.65 mg/kg (per dose) for all five doses

3.2.5 Treatment Compliance

When subjects are dosed at the investigational site, they will receive IMP directly from the Investigator or designee, under medical supervision to guarantee treatment compliance. The date and start and stop time of each infusion as well as the volume administered will be recorded in the source documents and transcribed to the CRF.

4. GENERAL CONSIDERATIONS FOR DATA ANALYSIS

Apart from the interim results, the analysis will be done upon study completion and database lock.

All continuous data will be summarized using N, mean, standard deviation (SD), first quartile (Q1), median (Q2), third quartile (Q3), minimum, and maximum value. Summary statistics for categorical variables will contain count and percentage. For log-normal endpoints, geometric mean (GM), geometric standard deviation (GSD), and geometric coefficient of variation (GCV) will also be produced. Summary results will be provided for each treatment group. All

tabulations will be based on pooled data across centers.

All statistical tests will be two-sided and tested at the 10% level of significance. If required, the nominal alpha level for the final analysis of the primary endpoint will be adjusted using Equation (11) of Mehta and Pocock (2009) as detailed in Section 9.3.

Analyses will be performed using SAS® software for Windows, version 9.4 or higher (SAS, Cary, NC), except where other software may be deemed more appropriate.

Subject data will be listed, sorted by treatment group, and subject number. When applicable, listings will be additionally sorted by visit and assessment date/time.

[redacted] will perform all efficacy and safety statistical analyses described in this SAP. [redacted] will manage the bioanalysis of pharmacokinetic (PK) measurements of the study and descriptive statistics of drug concentrations over time will be performed and provided by [redacted].

4.1 Analysis Quality Control Procedures

Once all the source verification is complete, all queries are resolved, and the database has been updated appropriately, the database will be locked and made available to [redacted] Biostatistics for final analysis.

Data may be pulled by [redacted] Biostatistics for the interim analysis at a time when source verification and query resolution is ongoing.

All SAS programs used to create analysis data sets, tables, and listings are double programmed. The SAS outputs will be compared, and the programs will be updated until the outputs match.

4.2 Analysis Sets

The Intent to Treat (ITT) set will consist of all randomized subjects.

The Modified Intent to Treat (mITT) set will consist of all subjects in the ITT set who received at least 1 dose of IMP. The mITT set will be used for the primary analysis of efficacy endpoints. Subjects will be included in the efficacy analysis based on randomized treatment group.

The Safety Analysis (SAF) set will consist of all subjects in the mITT set; however, subjects will be included in the analysis based on treatment actually received.

The Per Protocol (PP) set will consist of all subjects in the mITT set who did not have any major protocol deviations or other conditions (e.g., insufficient exposure) that were judged to influence the efficacy analysis. Prior to database lock, subjects who had a major protocol deviation will be identified in a blinded manner by the study team.

The PK Analysis (PK) set will be used for the PK evaluation and will consist of all randomized subjects with no relevant protocol deviations affecting the evaluation of the PK parameters.

4.3 Assessment Windows

SAP analysis visit windows will be constructed for data collected at protocol specified visits after the first dose of IMP administration. The analysis visit windows are wider than the protocol-defined visit windows to ensure inclusion of data points in the analysis. The date and

time of data collection will be used to determine the analysis visit window relative to the date and time of the first dose of IMP. Table 1 shows the post-baseline analysis visit windows.

Relative days in the study (ADY) will be calculated according to the following rule:

- Concerned date < reference date: ADY = concerned date – date of 1st IMP administration
- Concerned date ≥ reference date: ADY = concerned date – date of 1st IMP administration + 1

Per parameter and analysis window, the value closest to the target relative day will be used in analysis tables, other values will only be listed. If more than one value is located at the same distance from the target, then the latest in time will be selected.

Table 1: Analysis Visit Windows

Visit	Target	Analysis Visit Window	
		**Lower limit**	**Upper limit**
Day 1 (Pre-dose)[1]	Day 1 prior to start of 1st IMP administration	Day of 1st IMP administration at 00:00	Day/time prior to start of 1st IMP administration
Day 1 (12h)	12 hours after 1st IMP administration	6 hours after 1st IMP administration	<18 hours after 1st IMP administration
Day 2 (24h)	ADY = 2<br>(24 hours after 1st IMP administration)	18 hours after 1st IMP administration	<36 hours after 1st IMP administration
Day 3 (48h)	ADY = 3<br>(48 hours after 1st IMP administration)	36 hours after 1st IMP administration	<60 hours after 1st IMP administration
Day 4 (72h)	ADY = 4<br>(72 hours after 1st IMP administration)	60 hours after 1st IMP administration	96 hours after 1st IMP administration
Day 7	ADY = 7	ADY = 5	ADY = 9
Discharge	No window		
Day 30	ADY = 30	ADY = 23	ADY = 37
Day 90	ADY = 90	ADY = 83	ADY = 97

Assessments with a date associated with relative days (ADY) 2, 3, and 4 without a time will also be assigned to Day 2, 3 and 4 visits if no other assessment with both date and time have assigned

[1] This visit is only to be used in the descriptive analysis of SCr and cystatin C (and corresponding eGFR values) over time

to that visit. If there are multiple assessments with the same relative days and no time, the average will be used.

The data collected at the visit "Discharge" will be summarized in two ways. First, the data will be summarized with the CRF visit "Discharge". Second, this visit will be remapped to an analysis visit based on the date and time of collection.

For analysis endpoints with cutoff values (e.g., AKI within 72 hours), the windows will not be used at the cutoff value (i.e., AKI within 72 hours, not 96 hours).

4.4 Handling of Dropouts or Missing Data

Missing demographic, baseline information, and safety data will be treated as missing; no method for imputation is planned.

Details of any missing data imputation or sensitivity analyses will be specified in the analysis methods for each of the efficacy endpoints, described in Section 6.

4.5 Multiple Comparisons

No adjustment for multiplicity will be performed.

4.6 Data Derivations and Transformations

The following derivations will be used in this study:

Study Day: date of assessment – date of first dose of IMP (+1 for assessments done on or after date of first date of IMP).

Baseline Observation: the last non-missing value prior to IMP administration.

Note baseline samples for serum creatinine and cystatin C will be collected at Day -1 (screening) for central laboratory analysis which will provide the baseline values for the purpose of relevant endpoint assessments. Only if the value at screening is missing, the definition above will be used.

Change from baseline: assessment result at specific post-dose timepoint – baseline assessment result.

Ideal body weight (IBW):

- Males: IBW = 50kg + (0.91kg x number of centimeters [cm] over 1.52m)
- Females: IBW = 45.5kg + (0.91kg x number of centimeters [cm] over 1.52m)

Time-corrected area under the curve (AUC): the area under the SCr [or cystatin C] concentration versus time curve following drug administration will be calculated using timepoints at Day 1 (12 hour), Day 2 (24 hour), Day 3 (48 hour) and Day 4 (72 hour). The individual log-transformed SCr [or cystatin C] values will be determined, and the AUC (utilizing planned times) will be calculated using the right Riemann sum. The formula for the right Riemann sum is:

$$A = \sum_{i=1}^{n} \Delta x_i f(x_i)$$

where Δx_i is the width of each of the n rectangles (0.5, 1, and 1, respectively) and $f(x_i)$ is the height at the right endpoint of its base, or

A = 0.5 x SCr(12h) + 0.5 x SCr(24h) + 1 x SCr(48h) + 1 x SCr(72h)

The time-corrected AUC (log-scale) is calculated as AUC/3.

Geometric calculations:

- Geometric mean (GM) is calculated as exp(m), where m is the arithmetic mean of the log-transformed data.
- Geometric standard deviation (GSD) is calculated as exp(s), where s is the standard deviation of the log-transformed data.
- Geometric coefficient of variation (GCV) is calculated as $\sqrt{\exp(s^2) - 1}$, where s is the standard deviation of the log-transformed data.

Estimate glomerular filtration rate (eGFR): this will be calculated utilizing the Chronic Kidney Disease-Epidemiology Collaboration (CKD-EPI) equation for SCr, Cystatin C, and SCr, and Cystatin C. Refer to Appendix B for further information.

Actual Dose: the actual dose for RMC-035 and placebo will be calculated as a relative dose, as well as an absolute dose. Note when all 50 mL are administered, expected dose and actual dose will be the same.

- Actual Relative Dose (mg/kg):

$$\left(\frac{\left(\frac{volume\ administered\ (mL)}{50\ mL}\right)\ x\ expected\ volume\ of\ IMP\ or\ Placebo\ (mL)\ x\ 6.0\ mg/mL\ concentrate}{body\ weight\ at\ screening\ (kg)} \right)$$

- Actual Absolute Dose (mg):

$$\left(\frac{volume\ administered\ (mL)}{50\ mL}\right)\ x\ expected\ volume\ of\ IMP\ or\ Placebo\ (mL)\ x\ 6.0\ mg/mL\ concentrate$$

Due to the pharmacokinetics of RMC-035, the pre-defined dose levels (prior to surgery), are based on renal function (eGFR by CKD-EPI equation with SCr) at screening (Day -1, Visit 1):

- Subjects with eGFR ≥60 mL/min/1.73m^2 will receive 1.3 mg/kg (per dose) for the first and second dose, followed by 0.65 mg/kg (per dose) for the third, fourth, and fifth dose
- Subjects with eGFR >30 and <60 mL/min/1.73m^2 will receive 0.65 mg/kg (per dose) for all five doses

To determine the expected volume of IMP (mL) for each subject at each of the five doses, the screening eGFR and weight will be utilized with the same formulas used to dose subjects at the beginning of the study (found below), rounding to one decimal place after the calculation has been made.

- Subjects with eGFR ≥60 mL/min/1.73m^2:
  Expected volume (mL) for first two doses = (1.3 mg/kg x weight (kg))/6.0 mg/mL
  Expected volume (mL) for last three doses = (0.65 mg/kg x weight (kg))/6.0 mg/mL
- Subjects with eGFR >30 and <60 mL/min/1.73m^2:
  Expected volume (mL) for all five doses = (0.65 mg/kg x weight (kg))/6.0 mg/mL

The Dosing Tables containing body weight, dose (mg), volume of IMP (mL), and volume of 0.9% sodium chloride (mL) used can be found in Appendix C.

Duration:

- Duration of surgery (hours): the duration between the beginning of surgery and end of surgery (exact date/time of skin closure – exact date/time of initial skin incision)
- Index ICU stay (days): the duration of stay in the ICU immediately following surgery or recovery room post-surgery until ICU discharge (date of ICU discharge – date of ICU admission + 1)
- Index hospital stay (days): the duration of stay in the hospital from the day of surgery to hospital discharge for the index surgery (date of hospital discharge for the index surgery – beginning date of surgery + 1)
- Duration of AKI (days): the number of days meeting the definition of AKI starting within 72 hours after first dose of IMP until resolution (observed resolution date – AKI onset date + 1).

  Note that AKI onset should be within 72 hours of first dose of IMP. Duration of AKI will be calculated for all subjects. Subjects who do not develop AKI will have a duration of 0 days. Subjects who die without meeting AKI criteria will have a duration of 0 days. If status regarding AKI is not available, the subject will be classified as not having AKI. If resolution is not reached by Day 90, it is assumed they are resolved at Day 91. The minimum duration of AKI for a subject with AKI is 1. A subject without AKI has a duration of 0.

Acute Kidney Injury (AKI): AKI (yes/no) is defined as a subject meeting any of the AKI severity stages, where the AKI Severity Stages are:

Stage	Serum Creatinine	Urine Output
1	1.5 to 1.9 times baseline within 7 days<br>OR<br>≥0.3 mg/dL (≥26.5 µmol/L)	<0.5 mL/kg/h for 6 to <12 hours
2	2.0-2.9 times baseline within 7 days	<0.5 mL/kg/h for ≥12 hours
3	3.0 times baseline within 7 days<br>OR<br>4.0 mg/dL (≥353.6 µmol/L)<br>OR<br>initiation of renal replacement therapy	<0.3 mL/kg/h for ≥24 hours<br>OR<br>anuria for ≥12 hours

Serum Creatinine and Urine Output results are to be rounded to one decimal place before the Stage is determined.

AKI persistence: persistent AKI (yes/no) is defined as an AKI developing within 72 hours after first dose of IMP and with a duration of ≥72 hours. Persistence will also be assessed per AKI severity stage.

Fridericia's formula for QT interval correction (QTcF):

$$QTcF\ (msec) = QT\ (msec)\ x\ \left(\frac{Ventricular\ Rate\ (bpm)}{60}\right)^{1/3}$$

4.6.1 Primary Efficacy Endpoint Considerations

For the primary endpoint of AKI, missing data and untimely assessments will be handled as follows:

- If subject is missing the screening (Day -1) SCr value, the last non-missing value prior to the first dose of IMP will be used.
- Subjects with no SCr and no UO will be classified as not having AKI unless the subject has dialysis treatment.
- Any death within 72 hours after first dose of IMP will be classified as not having AKI unless the subject had met the AKI criteria before expiring.

4.6.2 Other Efficacy Endpoint Considerations

For the secondary endpoint dialysis treatment (for any reason) within 72 hours and within 7 days after end of surgery, missing data and untimely assessments will be handled as follows:

- Any death within 72 hours after first dose of IMP will be classified as not having dialysis unless the subject had dialysis before expiring.

For the secondary endpoint dialysis-free days from end of surgery to Day 30 and Day 90, missing data and untimely assessments will be handled as follows:

- Any periods without evidence of dialysis are considered free of dialysis, including periods where subjects expire.

5. STUDY SUBJECTS

5.1 Disposition of Subjects

A table of frequency counts and percentages of all subjects in the ITT, mITT, PP, and Safety sets will be provided. Subject disposition including study completion status and reasons for early termination will be tabulated by treatment group and overall. A by subject listing will be provided.

Completion of the study is defined as the time at which the last randomized subject completes the final follow-up period visit, is considered lost to follow-up, withdraws consent, or dies.

5.2 Protocol Deviations

Distribution of the types of protocol deviations and the number of subjects in the mITT set that deviate from the protocol will be tabulated by study treatment. A listing of all protocol deviations will be provided.

5.3 Demographic and Baseline Characteristics

Demographic data will be collected and will include gender, age, race, and ethnicity. Demographic data, risk factors for AKI, and other relevant baseline characteristics will be summarized by treatment group and overall using summary statistics for the subjects in the mITT set. Surgical details for the cardiac surgery including types and location of surgery, surgical duration, CPB details, and vital assessments will be summarized and listed as well. Refer to Appendix D for a listing of risk factors.

5.4 Medical History

Medical/surgical history and disease characteristics, a listing of relevant past diseases (within 5 years prior to Screening) and current diseases (ongoing at the time of Screening), and the details of active diseases will be obtained from medical records and recorded in the CRF.

All medical conditions and surgical procedures will be classified by system organ class (SOC) and preferred term (PT) using the Medical Dictionary for Regulatory Activities (MedDRA). The number and percent of subjects with each medical condition and surgical procedure will be presented for each SOC and PT for the mITT set for each study treatment and overall.

5.5 Prior and Concomitant Medications

Prior prescription and non-prescription medications (e.g., over-the-counter drugs and herbal supplements) taken within 30 days prior to scheduled date of surgery up to the date of consent will be obtained and all medications (including anesthesia) given before and during surgery will be collected. Medications are classified as prior if the stop date is before or on the day the ICF is signed.

Prior and concomitant medications will be coded using World Health Organization (WHO) drug classifications. The number and percent of subjects in the mITT set using concomitant medications will be tabulated by Anatomical, Therapeutic, and Chemical (ATC) level 4 and preferred name for each study treatment and overall.

6. EFFICACY ANALYSIS

Efficacy analyses will be performed in mITT set and will consist of all subjects in the ITT set who received at least 1 dose of IMP. Additionally, the primary and each of the key secondary endpoints will also be analyzed using the PP set, only if there are differences between the mITT and PP sets.

6.1 Primary Efficacy Endpoint and Analysis

The primary efficacy endpoint is AKI within 72 hours after first dose of IMP based on SCr and/or UO. If one of the two results (SCr or UO) indicates a subject has AKI (based on the stages below) and the other doesn't, the subject will be considered as having AKI.

Stage	Serum Creatinine	Urine Output
1	1.5 to 1.9 times baseline within 7 days<br>OR<br>≥0.3 mg/dL (≥26.5 µmol/L)	<0.5 mL/kg/h for 6 to <12 hours
2	2.0-2.9 times baseline within 7 days	<0.5 mL/kg/h for ≥12 hours
3	3.0 times baseline within 7 days<br>OR<br>4.0 mg/dL (≥353.6 µmol/L)<br>OR<br>initiation of renal replacement therapy	<0.3 mL/kg/h for ≥24 hours<br>OR<br>anuria for ≥12 hours

This endpoint will be analyzed by the Cochran-Mantel-Haenszel estimate of the common relative risk (RMC-035 vs. placebo) across the four stratification groups formed by region (North America and Europe) and eGFR at Day -1 (≥60 and <60 mL/min/1.73m^2). The estimate of the common relative risk, 90% confidence interval (CI) utilizing Greenland and Robins, and p-value will be reported. In addition, the proportion of subjects with AKI within 72 hours after first dose of IMP and its 90% CI will be calculated for each treatment group.

The hypotheses to be tested are as follows:

- Null hypothesis: the proportion of subjects developing AKI within 72 hours after first dose of IMP is the same for the RMC-035 and the placebo treatment groups ($p_{RMC\text{-}035} = p_{placebo}$)
- Alternative hypothesis: the proportion of subjects developing AKI within 72 hours after first dose of IMP is different for the RMC-035 and the placebo treatment groups ($p_{RMC\text{-}035} \neq p_{placebo}$)

The frequency and proportion for AKI within 72 hours after first dose of IMP will be reported by strata and treatment group, as well as by treatment group (pooling all four strata).

See Appendix E for the derivation of urine output utilizing disjoint intervals in determining whether a subject has AKI. Other imputations for the primary endpoint can be found in Section 4.6.1.

6.1.1 Supplementary Analyses of the Primary Endpoint

The first supplementary analysis will be performed for AKI within 72 hours after first dose of IMP based on SCr ONLY, as described above in Section 6.1.

The second supplementary analysis will be performed for AKI within 72 hours after first dose of IMP as described above in Section 6.1; the only difference being any deaths within 72 hours will be counted as having AKI.

The third supplementary analysis will be performed for AKI within 72 hours after first dose of IMP as described above in Section 6.1; the only difference being UO is not derived from the actual body weight, but from the ideal body weight.

The fourth supplementary analysis will be performed for AKI within 72 hours after first dose of IMP, as described above in Section 6.1; the only difference being UO will be derived from rolling 6-hour intervals, instead of disjoint intervals. See Appendix E for the derivation of urine output utilizing rolling intervals in determining whether a subject has AKI.

6.1.2 Sensitivity Analyses of the Primary Endpoint

The primary endpoint will also be analyzed using the PP set, assuming there are differences between the mITT and PP sets.

6.2 Key Secondary Efficacy Endpoints and Analyses

The following key secondary endpoints will be analyzed on the mITT set.

- Time-corrected area under the curve (AUC) of SCr for Day 1 to Day 4 (72 hours after first dose of IMP)
- Duration of AKI defined as the number of days meeting the definition of AKI starting within 72 hours after first does of IMP until resolution

Time-corrected area under the curve (AUC) of SCr for Day 1 to Day 4 (72 hours after first dose of IMP)

For the key secondary AUC endpoint, Robust regression using Huber weights with a cut-off of 2 will be utilized on AUC for serum creatine, adjusting for baseline log-transformed SCr and region. The geometric weighted least square means of the time-corrected AUC will be obtained for each RMC-035 and placebo by transforming the model estimates back to the original scale. Between-arms differences of the Robust regression estimates will be transformed back to the original scale using $100\times(e^{(statistic)}-1)$ and reported as geometric mean percent changes. The estimate of the geometric mean percent change from baseline per arm, 90% confidence interval (CI), and p-value will be reported. The relative difference (RMC-035 vs. placebo) and its 90% CI will also be reported.

In order to handle missing data, the analysis will be performed over 3 phases: an imputation phase, analysis phase, and pooling phase, as described below.

Imputation phase

The first step of the imputation phase will create data with a monotone data structure having imputed 20 datasets separately within each treatment arm. The number of burn-in iterations will be set to 200, and observations will be sampled every 200 iterations within the same chain for each imputed dataset. An autocorrelation plot will be produced to confirm that NITER is long enough to ensure imputations are independent and a trace plot also produced to confirm there are no systematic trends.

```
proc mi data=data nimpute=20 seed=2019301 out=mono;
      var BASE VAL1-VAL7;
      mcmc chain=single nbiter=200 niter=200 impute=monotone;
      by TRT;
run;
```

where BASE, and VAL1-VAL7 are the log-transformed SCr values at baseline and at Day 1 (12 hour), Day 2 (24 hour), Day 3 (48 hour), Day 4 (72 hour), Day 7, Day 30, and Day 90.

In the second step of the imputation phase, data will be multiply imputed using a regression method sequentially imputing data across successive visits separately by treatment arm from each dataset imputed in the first step. This analysis assumes data are missing at random conditional on previous SCr recordings, which is the same assumption used in a MMRM model and therefore would be expected to give equivalent results (O'Kelly & Ratitch).

```
proc mi data=mono nimpute=1 SEED=2019301 OUT=imputed;
      class TRT REGION;
      var REGION BASE VAL1-VAL7;
      monotone regression;
      by TRT _imputation_;
run;
```

where BASE, and VAL1-VAL7 are the log-transformed SCr values at baseline and at Day 1 (12 hour), Day 2 (24 hour), Day 3 (48 hour),Day 4 (72 hour), Day 7, Day 30, and Day 90.

Missing data are therefore multiply imputed conditional on outcomes previously observed for the same patient and based on the trajectory of outcomes of other patients in the same treatment arm.

Analysis phase

In the analysis phase, the AUC will be calculated for each patient within each imputed dataset. Each time point will be given the weight stated in Section 4.6. Then AUC of the log-transformed data will be analyzed using Robust Regression with independent variables of treatment and log-transformed baseline SCr. M-estimation will be used with Huber weights and a cut-off value of 2 with the median method used to estimate the scale parameter.

```
proc robustreg data=auc method=m (wf=huber(c=2) scale=med) outest=est1
covout;
      by _imputation_;
      class TRT REGION;
      model AUC = BASE TRT REGION;
run;
```

Pooling phase

In the pooling phase, estimated treatment effects and associated standard errors from each imputation will be combined using Rubin's rules to provide an overall treatment effect, associated 90% confidence interval (CI), and 2-sided p-value.

```
proc mianalyze data=est1;
      modeleffects estimate;
      stderr stderr;
      ods output ParameterEstimates=est2;
run;
```

Sensitivity analysis

A sensitivity analysis will be performed using a mixed model for repeated measurements (MMRM) on the log-transformed SCr values. The log-transformed values at Day 1 (12 hour), Day 2 (24 hour), Day 3 (48 hour) and Day 4 (72 hour) will be modelled in the response vector. An 8-level visit-by-treatment class variable and a 4-level visit-by-baseline covariate will be included (i.e., between arm differences will only be allowed at the post-baseline visits). In addition, region will be used as a fixed factor. The AUC will be obtained as a function of the model parameters utilizing SAS estimate statements.

Duration of AKI (within 72 hours) defined as the number of days meeting the primary endpoint until resolution

The key secondary AKI duration endpoint will be analyzed using the Wilcoxon Rank-Sum test with stratification groups formed by region (North America and Europe) and eGFR at Day -1 (≥60 and <60 mL/min/1.73m^2). The estimate of median difference, 90% confidence interval (CI) for the median difference utilizing Hodges-Lehman, and p-value will be reported.

6.3 Other Secondary Efficacy Endpoints and Analyses

SCr, cystatin C, and eGFR at 12, 24, 48, and 72 hours, respectively, and at Day 7/discharge, Day 30, and Day 90

For SCr, cystatin C, and eGFR, descriptive statistics will be produced by analysis visit on the raw untransformed data. eGFR values will be calculated from the raw untransformed SCr data and cystatin C data. This summary will include all analysis visits including the screening and Day 1 (Pre-Operative) visit. Columns for actual values, changes from baseline, and percent change from baseline (calculated as 100*(change/baseline)) will be included. Changes from baseline will be reported for all visits except screening.

For SCr, cystatin C, and eGFR, robust regression will be utilized to determine the between-arm difference at each visit. The existing 3-phase multiple imputation approach detailed above will be re-used for this analysis. After imputation, all observed and imputed log-transformed SCr and cystatin C values will be transformed back to the original scale. Imputed eGFR values will be calculated from the imputed SCr data, as well as the imputed cystatin C data. In the analysis step, AUC (dependent variable) will be replaced by either VAL1, VAL2, VAL3, VAL4, VAL5, VAL6, or VAL7 representing the missing-data-free SCr, cystatin C, or eGFR values at Day 1 (12 hour), Day 2 (24 hour), Day 3 (48 hour), Day 4 (72 hour), Day 7, Day 30, and Day 90. The untransformed baseline values will be subtracted from the resulting values in order to obtain, for each subject, absolute changes from baseline to each of the 7 aforementioned timepoints.

The analysis phase will be therefore slightly different from that done for AUC in the sense that the model will be applied to the absolute change from baseline. The untransformed baseline values will be used as an independent variable. Robust regression estimates will be averaged

over imputations using Rubin's rules. The estimated mean change from baseline per arm and respective 90% CI and p-value will be calculated. Additionally, the estimated between-arm difference will be calculated, along with the respective 90% CI and p-value.

A sensitivity analysis will be performed using a mixed model for repeated measurements (MMRM) separately for each of the untransformed SCr, cystatin C, and eGFR values. The (untransformed) change from baseline values for Day 1 (12 hour), Day 2 (24 hour), Day 3 (48 hour), Day 4 (72 hour), Day 7/Discharge, Day 30 & Day 90 will be modelled in the response vector. A 14-level visit-by-treatment class variable and a 7-level visit-by-screening and visit-by-pre-operation covariate will be included. In addition, region will be used as a fixed factor. For the sensitivity analysis only, subjects without a screening value will use the Day 1 pre-operative value for screening. Additionally for the sensitivity analysis only, subjects without a Day 1 pre-operative value will use the screening value for Day 1 pre-operative. The estimated mean change from baseline per arm and respective 90% CI and p-value will be calculated. Additionally, the estimated between-arm difference will be calculated, along with the respective 90% CI and p-value.

Change from baseline, up to Day 7/discharge, of peak SCr and cystatin C

The raw untransformed data will be utilized for this analysis. The dependent variable is peak change from baseline (peak value-baseline value). The untransformed baseline values will be used as an independent variable, as well as region. The estimated between-arm difference will be calculated, along with the respective 90% CI and p-value.

Time-corrected AUC of Cystatin C for Day 1 to Day 4 (72 hours after first dose of IMP)

This endpoint will be analyzed as described in Section 6.2 for the key secondary AUC endpoint, utilizing cystatin C instead of serum creatinine.

Dialysis treatment (for any reason) within 72 hours and within 7 days after end of surgery

This endpoint will be analyzed as described in Section 6.1.

The frequency and proportion for dialysis treatment within 72 hours and within 7 days after first dose of IMP will be reported by strata and treatment group, as well as by treatment group (pooling all strata).

Dialysis-free days from end of surgery to Day 30 and Day 90, respectively

This endpoint will be analyzed as described in Section 6.2 for the key secondary AKI duration endpoint.

Dialysis-free duration in 30 and 90 days, is the sum of all dialysis-free durations of a subject in the time periods, respectively. Number of dialysis-free days will be calculated for all subjects. If status regarding dialysis is not available, the subject will be classified as not having undergone dialysis.

Dialysis-free days will be counted when the subject achieves at least 7 consecutive days without dialysis.

For the Day 30 analysis, subjects who may have become dialysis independent (defined as

7 consecutive dialysis-free days) between Days 24 and 30 will need to be followed for the number of additional days to determine whether 7 consecutive dialysis-free days have occurred. Therefore, daily dialysis recording for these subjects may need to be extended into the period from Day 31 to Day 36). This will be similar for the analysis of Day 90.

The number and frequency of dialysis-free subjects from end of surgery to Day 30 will be provided, as well as for Day 90.

MAKE by Day 30 and 90

A subject will be considered as having a MAKE at Day X, where X=30 and 90, if the subject experiences:

- Death through Day X; or
- Any dialysis through Day X; or
- A ≥25% reduction of estimated glomerular filtration rate (eGFR) on Day X compared to baseline using the Chronic Kidney Disease-Epidemiology Collaboration (CKD-EPI) equations (**utilizing SCr only**)

The analysis for Day 30 and Day 90 will be examined separately.

This endpoint will be analyzed as described in Section 6.1.

Additionally, the number and frequency of subjects who meet each of the three criteria listed above will be displayed separately.

A sensitivity analysis utilizing the alternative definition of MAKE X will be performed as described above, where the alternative definition utilizes cystatin C only. A second sensitivity analysis utilizing the alternative definition of MAKE X will be performed as described above, where the alternative definition utilizes SCr and cystatin C. See Appendix B for the three calculations.

AKI within 72 hours after first dose of IMP based on cystatin C and/or UO (AKI of any stage/severity defined as cystatin C ≥1.5 times baseline, OR UO <0.5 mL/kg/h for ≥6 hours)

This endpoint will be analyzed as described in Section 6.1.

The frequency and proportion for AKI within 72 hours after first dose of IMP will be reported by strata and treatment group, as well as by treatment group (pooling all strata).

AKI within 7 days after first dose of IMP (based on SCr and/or UO criteria, or cystatin C and/or UO criteria)

This endpoint will be analyzed as described in Section 6.1. The frequency and proportion for AKI within 7 days after first dose of IMP will be reported by strata and treatment group, as well as by treatment group (pooling all strata).

AKI persistence, defined as an AKI developing within 72 hours after first dose of IMP and with a duration of ≥72 hours.

This endpoint will be analyzed as described in Section 6.1. The frequency and proportion for AKI persistence will be reported by strata and treatment group, as well as by treatment group (pooling all strata).

In addition, persistence will be tabulated by AKI severity and treatment group.

AKI severity stage within 72 hours and within 7 days after first dose of IMP.

Descriptive statistics (frequency and proportions) will be displayed by treatment group. The highest stage a subject reaches within the respective timeframe will be used. For example, a subject developing AKI stage 1 and subsequently stage 2 and/or 3 will counted as 3.

Post-baseline changes in urine albumin to creatinine ratio (UACR) and urine protein to creatinine ratio (UPCR) at Day 4, Day 30, and Day 90

The data will be reported separately.

Pharmacokinetics of RMC-035 in plasma (AUC and C_{max})

[redacted] will conduct bioanalysis of PK samples with PK parameters and reporting carried out by CTC. Descriptive statistics of drug concentrations over time will be provided by dose group and by renal function at baseline.

Presence and titers of ADA at Day 1 (pre-surgery), Day 30, and Day 90

Presence and titers of ADA and ADA characterization will be reported separately.

Characteristics of ADA developed at Day 30 and Day 90 with regards to isotype, neutralizing capacity, and cross-reactivity with endogenous alpha-1-microglobulin (A1M)

If ADAs are present, the presence and further characterization will be reported separately.

6.4 Exploratory Efficacy Endpoints and Analyses

Kidney Biomarkers

Urine kidney injury molecule 1 (KIM-1), neutrophil gelatinase-associated lipocalin (NGAL), tissue inhibitor of metalloproteinase 2 (TIMP2), insulin-like growth binding factor protein 7 (IGFBP7), chemokine ligand 14 (CCL-14), interleukin-18 (IL-18), liver fatty acid binding protein (LFABP) and 8-hydroxy-2'-deoxyguanosine (8-OHdG) may be analyzed and reported separately.

Cardiac Biomarkers

Plasma N-terminal-pro-hormone BNP (NT-proBNP) and cardiac troponin I and T (cTnI, cTnT) may be analyzed and reported separately.

Immunologic Biomarkers

Biomarkers may be analyzed and reported separately.

Index ICU stay (in Days)

Data will be presented separately.

Index hospital stay (in Days)

Data will be presented separately.

Nature of subject discharge facility

Data will be presented separately.

Change from baseline to Day 90 for Medical Outcomes Study (MOS) 36-Item Short Form Survey Instrument (SF-36)

These parameters will be reported separately.

Change from baseline to Day 90 for European Quality of Life 5 Domain 5-Level Score (EQ-5D-5L)

These parameters will be reported separately.

The European Quality Visual Analog Scale (VAS)

These parameters will be reported separately.

6.5 Subgroup Analysis

A subgroup analysis for the primary endpoint will be conducted by Day -1 eGFR (≥60 and <60 mL/min/1.73m^2) and region (North America and Europe).

7. SAFETY ANALYSIS

Safety analyses will be conducted on the Safety Analysis Set (SAF), which includes all randomized subjects who received at least 1 dose of RMC-035. To characterize the safety profile, the number and percentage of subjects with TEAEs and AEs per dose group will be tabulated per System Organ Class (SOC) and Preferred Term (PT) according to the Medical Dictionary for Regulatory Activities (MedDRA) version 24.1.

Descriptive statistics will be provided for laboratory tests (hematology, biochemistry, urinalysis) and vital signs (pulse rate, respiratory rate and blood pressure) by visit and for the changes from baseline to each visit split by dose group.

All adverse events (AEs) will be collected from initiation of the first IMP administration through the Day 30 study visit. All serious adverse events (SAEs) will be collected from the signing of the informed consent form (ICF) through the end of study visit.

7.1 Extent of Exposure

The total number of infusions received, the expected total infusion dose, and the actual infusion dose administered will be summarized and presented.

7.2 Adverse Events

An adverse event (AE) is any untoward medical occurrence in a clinical study subject, temporally associated with the use of medicinal product, whether or not considered related to this IMP. An AE can therefore be any unfavorable and unintended sign (including an abnormal

laboratory finding), symptom, or disease (new or exacerbated) temporally associated with the use of medicinal product.

All AEs will be collected from initiation of the first IMP administration through the Day 30 study visit.

All SAEs will be collected from the signing of the ICF through the EOS visit.

7.2.1 Treatment-emergent Adverse Events

A treatment-emergent adverse event (TEAE) is any AE that occurs after the initial IMP administration through 72 hours (inclusive) after the final IMP administration.

7.2.2 Post-treatment Adverse Event

A post-treatment adverse event (PTAE) is any AE that occurs later than 72 hours after the final IMP administration.

7.2.3 Adverse Event Severity

The intensity of an event describes the degree of impact upon the subject and/or the need for, and extent of, medical care necessary to treat the event. Intensity will be assessed according to the CTCAE scale v5.0, as follows:

Grade 1 Mild; asymptomatic or mild symptoms; clinical or diagnostic observations only; intervention not indicated.

Grade 2 Moderate; minimal, local or noninvasive intervention indicated; limiting age-appropriate instrumental Activities of Daily Living (ADL).

Grade 3 Severe or medically significant but not immediately life-threatening; hospitalization or prolongation of hospitalization indicated; disabling; limiting self-care ADL.

Grade 4 Life-threatening consequences; urgent intervention indicated

Grade 5 Death related to AE

Instrumental ADL refers to preparing meals, shopping for groceries or clothes, using the telephone, managing money, etc. Self-care ADLs refer to bathing, dressing and undressing, feeding self, using the toilet, taking medications, and not bedridden.

For events not listed in the CTCAE scale, intensity grading will occur according to the following scale:

Grade 1 Mild; asymptomatic or mild symptoms; clinical or diagnostic observations only; intervention not indicated.

Grade 2 Moderate; minimal, local or noninvasive intervention indicated; limiting age-appropriate instrumental Activities of Daily Living (ADL).

Grade 3 Severe or medically significant but not immediately life-threatening; hospitalization or prolongation of hospitalization indicated; disabling; limiting self-care ADL.

Grade 4 Life-threatening consequences; urgent intervention indicated

Grade 5 Death related to AE

An AE of severe intensity may not be considered serious.

7.2.4 Adverse Event Relationship to Study Medication

The investigator is obligated to assess and record the relationship between the IMP and each occurrence of each AE/SAE according to the following definitions:

- **Not related (unlikely)**
  - Does not follow a reasonable temporal sequence from drug administration
  - Is readily explained by the subject's clinical state or by alternative etiologies such as underlying disease(s), concomitant therapy and other risk factors
- **Related (possible/probable)**
  - Follows a reasonable temporal sequence from IMP administration
  - Cannot be reasonably explained by the known characteristics of the subject's clinical state

Alternative etiology should be provided for all AEs assessed as possibly related to IMP.

The Investigator will use clinical judgment to determine the relationship.

7.2.5 Serious Adverse Events

A serious adverse event (SAE) is defined as an AE that meets at least one of the following serious criteria:

- Results in death
- Is life-threatening
- Requires inpatient hospitalization or prolongation of existing hospitalization
- Results in persistent disability/incapacity
- Is a congenital anomaly/birth defect
- Other medically important serious event

7.2.6 Adverse Events of Special Interest

Injection Site Reaction (ISR) and Infusion Related Reaction (IRR) of grade 3 or higher are considered Adverse Events of Special Interest and must be reported within 24 hours following the SAE. The CTCAE grading for ISR and IRR are provided in Table 2 below.

Table 2: CTCAE Grading for Injection Site Reactions (ISR) and Infusion Related Reactions (IRR)

CTCAE Term	Grade 1	Grade 2	Grade 3	Grade 4	Grade 5	Definition
MedDRA PT: Injection site reaction<br>MedDRA SOC: General disorders and administration site conditions	Tenderness with or without associated symptoms (e.g., warmth, erythema, itching)	Pain; lipodystrophy; edema; phlebitis	Ulceration or necrosis; severe tissue damage; operative intervention indicated	Life-threatening consequences; urgent intervention indicated	Death	A disorder characterized by an intense adverse reaction (usually immunologic) developing at the site of an injection.
MedDRA PT: Infusion related reaction<br>MedDRA SOC: Injury, poisoning and procedural complications	Mild transient reaction; infusion interruption not indicated; intervention not indicated	Therapy or infusion interruption indicated but responds promptly to symptomatic treatment (e.g., antihistamines, NSAIDS, narcotics, IV fluids); prophylactic medications indicated for ≤24 hrs	Prolonged (e.g., not rapidly responsive to symptomatic medication and/or brief interruption of infusion); recurrence of symptoms following initial improvement; hospitalization indicated for clinical sequelae	Life-threatening consequences; urgent intervention indicated	Death	A disorder characterized by adverse reaction to the infusion of pharmacological or biological substances.

Abbreviations: CTCAE=Common Terminology Criteria for Adverse Events; IV=intravenous; MedDRA=Medical Dictionary for Regulatory Activities; NSAID=non-steroidal anti-inflammatory drug; SOC=system organ class

7.2.7 Adverse Event Summaries

The number and percentage of subjects with TEAEs and PTAEs per dose group will be tabulated per System Organ Class (SOC) and Preferred Term (PT) according to the Medical Dictionary for Regulatory Activities (MedDRA) version 24.1.

For treatment-emergent AEs (TEAEs), the following will be summarized and presented for the Safety set:

i. An overall summary of TEAEs, which includes:

   a. the number and percentage of subjects experiencing a TEAE

   b. the number and percentage of subjects experiencing a TEAE by strongest relationship to investigational product

   c. the number and percentage of subjects experiencing a TEAE by greatest intensity

d. the number and percentage of subjects experiencing a TEAE leading to permanent discontinuation of the IMP

e. the number and percentage of subjects experiencing a treatment emergent serious adverse event (TESAE)

f. the number and percentage of subjects experiencing a TESAE by strongest relationship to investigational medicinal product

g. the number and percentage of subjects experiencing a TEAE leading to death

h. the number and percentage of subjects experiencing a TEAE Injection Site Reaction (h) of grade 3 or higher

i. the number and percentage of subjects experiencing a TEAE Infusion Related Reaction (IRR) of grade 3 or higher

j. the number and percentage of subjects experiencing a TEAE suggestive of IRRs on days of treatment

ii. the number and percentage of subjects experiencing a TEAE by SOC, PT, and the strongest relationship to investigational product

iii. the number and percentage of subjects experiencing a TEAE by SOC, PT, and the greatest intensity

iv. the number and percentage of subjects experiencing a TEAE leading to permanent discontinuation of the IMP by SOC and PT

v. the number and percentage of subjects experiencing a TESAE by SOC and PT

vi. the number and percentage of subjects experiencing a TESAE by SOC and PT, and the strongest relationship to the investigational medicinal product

vii. the number and percentage of subjects experiencing a TEAE leading to death

viii. the number and percentage of subjects experiencing a TEAE Injection Site Reaction (ISR) of grade 3 or higher by SOC and PT

ix. the number and percentage of subjects experiencing a TEAE Infusion Related Reaction (IRR) of grade 3 or higher by SOC and PT

x. the number and percentage of subjects experiencing a TEAE suggestive of IRRs on days of treatment by PT and Site

The same tables described above will be presented for post-treatment AEs (PTAEs) for the Safety set, as applicable.

In the overall summary of TEAEs and PTAEs tables (i), besides tabulating the number and percentage of subjects, the total number of TEAE episodes will also be provided. If a subject has repeated episodes of a particular TEAE, all episodes will be counted in the summary table.

In the remaining summary tables, the incidences of TEAEs and PTAEs will be calculated by dividing the number of subjects who have experienced the event by the total number of subjects in the SAF set. Thus, the incidence of TEAEs is shown in terms of the total number of subjects

and not in terms of the total number of episodes. If a subject has repeated episodes of a particular TEAE or PTAE, only the most severe episode, or the episode with the strongest causal relationship to IMP, will be counted in the summary tables.

A subject with more than one type of TEAE or PTAE in a particular SOC will be counted only once in the total of subjects experiencing TEAEs or PTAEs in that particular SOC. Since a subject could have more than one type of TEAE/PTAE within a particular SOC, the sum of subjects experiencing different TEAEs/PTAEs within the SOC could appear larger than the total number of subjects experiencing TEAEs/PTAEs in that SOC. Similarly, a subject who has experienced a TEAE/PTAE in more than one SOC will be counted only once in the total number of subjects experiencing AEs in all SOCs.

All occurrences of all AEs will be listed for each subject, grouped by treatment group. The listing will contain the following information: treatment group, event verbatim term, SOC, PT, maximum intensity, relationship to investigational product, date and day of onset, date and day of resolution, treatment given to treat the adverse event, the outcome, whether the event was an SAE, and which serious criteria were applicable, whether it led to permanent discontinuation of the IMP, whether it is a TEAE, and whether it is a PTAE. Listings will be sorted by subject identification number, onset date, SOC, and PT. If the onset date is completely missing, then these events will be presented first. If the onset date is missing a month or a day, then these events will be presented before any complete dates.

7.3 Clinical Laboratory Assessments

Laboratory assessments will include hematology, clinical chemistry, liver function labs, urinalysis, urine albumin and protein creatinine ratio, as well as other screening tests.

Continuous laboratory parameters will be summarized by parameter, treatment group, and analysis visit. Actual values and changes from baseline will be tabulated separately by each visit, split by dose group. This tabulation will include the maximum and the minimum post-baseline. Shift tables will be produced for laboratory reference ranges using the lowest and highest post-baseline level.

Laboratory parameter results from unscheduled visits will be excluded from table summaries but will be included in data listings. When there are repeat measurements for a given visit, only the last measurement will be used in the table summaries.

Listings will include flags for values outside of the reference ranges, and clinical significance if a laboratory result is deemed abnormal, if available.

Box plots per analysis visit and treatment arm will be produced for each continuous laboratory parameter. One page will be produced for each parameter. This plot will include the reference ranges as horizontal lines.

A frequency table of the number and percent of subjects with potentially clinically significant liver value elevations will be provided.

7.4 Vital Signs

Vital signs will include body temperature (°C), systolic and diastolic blood pressure (mmHg), heart rate (bpm) and respiratory rate (breaths per min), and oxygen saturation (SpO2).

Continuous vital signs will be summarized by parameter, treatment group and analysis visit. Actual values and changes from baseline will be tabulated separately by each visit, split by dose group. This tabulation will include the maximum and the minimum post-baseline. Shift tables will be produced for potentially clinically significant vital sign values (worst values):

- Systolic blood pressure (mmHg): ≥ 140, ≥ 160, ≥ 180, < 90, < 60
- Diastolic blood pressure (mmHg): ≥ 90, ≥ 100, ≥ 120, < 60, < 45
- Heart rate (bpm): >100, > 115, > 130, < 60, < 50, < 40
- Body temperature (°C): > 38, < 35

Unscheduled visits will be excluded from the summaries but will be included in the data listings.

Box plots per analysis visit and treatment arm will be produced for each continuous vital sign. One page will be produced for each parameter. This plot will include the reference ranges as horizontal lines.

7.5 ECG

Continuous electrocardiogram interval measurements will be summarized by parameter, treatment group and analysis visit. Actual values and changes from baseline will be tabulated separately by each visit, split by dose group. This tabulation will include the maximum and the minimum post-baseline. Shift tables will be produced for potentially clinically significant ECG (worst values):

- QTcF (ms): > 450, > 480, > 500
- Change from baseline in QTcF (ms): >30, >60

Box plots per analysis visit and treatment arm will be produced for each continuous electrocardiogram interval measurements. One page will be produced for each parameter. This plot will include the reference ranges as horizontal lines.

7.6 Physical Examination

The Investigator or qualified designee will conduct the exams, determine findings, and assess any abnormalities as to clinical significance. The physical examination performed at screening should be comprehensive; all other physical examinations may be abbreviated and symptom driven.

8. SAMPLE SIZE AND POWER CALCULATIONS

For the primary endpoint, AKI within 72 hours after first dose of IMP, RMC-035 has been assumed to lead to a 30% relative risk reduction vs. placebo. The event rate in the placebo group has been assumed to be 50%. A sample size of 268 subjects dosed leads to a test power of 80% to show statistically significant results at a two-sided significance level (alpha) of 0.10. An interim analysis (IA) of the primary endpoint will be performed when 50% of the planned subjects have completed Visit 6. Conditional power (CP) given 'the current trend' will be calculated. Sample size may be increased to a maximum of 348 subjects dosed. Sample size will not be decreased.

It is planned that approximately 380 subjects (approximately 30% screen failure assumed) will be enrolled to ensure 268 subjects dosed with either RMC-035 or placebo in a 1:1 ratio. Based on results at an interim analysis, up to 348 subjects may be dosed, requiring approximately 500 subjects to be enrolled.

9. INTERIM ANALYSIS

An interim analysis (IA) of the primary endpoint (see Section 6.1) will be performed on the mITT set when 50% (n=134) of the planned randomized subjects have completed the study through discharge (Visit 6) and data have been reviewed and are available. Serum creatinine and urine output collected through Visit 6 will be included in the analysis. Conditional power (CP), the probability that the final analysis will be statistically significant, will be calculated. Sample size may be increased to a maximum of 348 randomized subjects. Sample size will not be decreased. The study may be stopped at the IA for futility reasons. Study eligibility criteria may also be modified.

Given the unblinded nature of this IA, an unblinded Data Monitoring Committee (DMC) of independent individuals not involved in any other study activities will be utilized. The Sponsor and all other CRO personnel will remain blinded. The unblinded materials, data, and analysis results will be stored securely and separately from blinded clinical material and will not be accessible by blinded personnel. Results of the IA will not be shared with the Sponsor or blinded CRO personnel; only a decision on whether sample size expansion criteria have been met will be shared with the blinded team.

Further details on the dissemination of IA results and rules governing adaptations will be provided in the Data Monitoring Committee (DMC) charter.

The following will be provided at the interim analysis:

- Table 14.1.3 Summary of Demographics – mITT Set
- Table 14.1.4 Summary of Baseline Characteristics – mITT Set
- Table 14.1.7 Summary of Surgical Details – mITT Set
- Table 14.2.1.1.1 Summary of Primary Endpoint: AKI based on SCr and/or UO, utilizing disjoint intervals – mITT Set
  Supplementary Analyses of Primary Endpoint
  - Table 14.1.1.1.6 (AKI based on SCr only) – mITT Set
  - Table 14.1.1.1.7 (AKI based on SCr and/or UO, Counting Deaths as AKI) – mITT Set
  - Table 14.1.1.1.8 (AKI based on SCr and/or UO, Using Ideal Body Weight instead of Actual Body Weight) – mITT Set
  - Table 14.1.1.1.9 (AKI based on SCr and/or UO, utilizing rolling intervals) – mITT Set
- Table 14.2.2.1.1 Summary of Key Secondary Endpoint: Time-Corrected AUC of Serum Creatinine for Day 1 to Day 4 (72 Hours After First Dose of IMP) – mITT Set
- Table 14.2.3.6 Summary of Key Secondary Endpoint: Time-Corrected AUC of Cystatin C for Day 1 to Day 4 (72 Hours After First Dose of IMP) – mITT Set

- Table 14.2.3.1 Summary of Other Secondary Endpoint: Serum Creatinine at Hour 12, 24, 48, and 72, and Day 7, 30, and 90 – mITT Set
- Table 14.2.3.2 Summary of Other Secondary Endpoint: Cystatin C at Hour 12, 24, 48, and 72, and Day 7, 30, and 90 – mITT Set
- Table 14.2.3.3 Summary of Other Secondary Endpoint: eGFR (calculated from Serum Creatinine) at Hour 12, 24, 48, and 72, and Day 7, 30, and 90 – mITT
- Table 14.2.3.4 Summary of Other Secondary Endpoint: eGFR (calculated from Cystatin C) at Hour 12, 24, 48, and 72, and Day 7, 30, and 90 – mITT Set
- Table 14.2.3.8 Summary of Other Secondary Endpoint: Dialysis-Free Days from End of Surgery to Day 30 and Day 90 – mITT Set
- Table F: Sample Size Assessment Utilizing Primary Endpoint (AKI) – mITT (see Section 9.1 and Appendix F)
- Figure 14.2.3.1 Lineplot of Geometric Means and Standard Errors (Descriptive Statistics) of Serum Creatinine – mITT Set
- Figure 14.2.3.2 Lineplot of Geometric Means and Standard Errors (Descriptive Statistics) of Cystatin C – mITT Set
- Figure 14.2.3.3 Lineplot of Geometric Means and Standard Errors (Descriptive Statistics) of eGFR (calculated from Serum Creatinine) – mITT Set
- Figure 14.2.3.4 Lineplot of Geometric Means and Standard Errors (Descriptive Statistics) of eGFR (calculated from Cystatin C) – mITT Set

In addition to the efficacy IA described above, one exploratory interim analysis of cardiac and kidney biomarkers is planned in the protocol (as of version 3.0). This biomarker IA is independent from the IA of the primary endpoint, and it is not subject to review and decision making by the DMC. Further details on the biomarker IA and the associated measures to minimize impact on data integrity will be documented separately.

9.1 Conditional Power

The conditional power at the interim analysis will be calculated as follows:

$$\mathrm{CP} = 1 - \Phi\left(\frac{z_{\alpha}\sqrt{n_2} - z_1\sqrt{n_1}}{\sqrt{\tilde{n}_2}} - \frac{z_1\sqrt{\tilde{n}_2}}{\sqrt{n_1}}\right)$$

where Φ is the CDF for $N(0,1)$, α is 0.10, $z_{\propto}$ is 1.645, and $z_1 = \frac{ln\ (RR_{MH})}{\sqrt{var[ln(RR_{MH})]}}$ is the z-score corresponding to the log-transformed relative risk statistic, and $\tilde{n}_2 = n_2 - n_1$ is the incremental sample size between stage 1 (interim analysis after n_1 subjects) and stage 2 (final analysis after n_2 subjects).

Where the estimate of the common relative risk is computed as:

$$RR_{MH} = {(\sum_h n_{h11}\ n_{h2.}/n_h)}\Big/{(\sum_h n_{h21}\ n_{h1.}/n_h)}$$

with 100(1-α/2)% confident limits:

$$(RR_{MH}\ x\exp(-z\hat{\delta}),\ RR_{MH}\ x\exp(z\hat{\delta}))$$

and

$$\hat{\delta}^2 = \widehat{Var}(\log((RR_{MH})) = \frac{\sum_h (n_{h1.}\ n_{h2.}\ n_{h.1-}\ n_{h2.}/n_{h11}\ n_{h21}\ n_h)/n_h^2}{(\sum_h n_{h11}\ n_{h2.}/n_h)\ (\sum_h n_{h21}\ n_{h1.}/n_h)}$$

Appendix F contains the table shell that will be produced at the time of the IA which includes the estimated treatment effect, sample size used, and conditional power.

9.2 Sample Size Readjustment

If the conditional power at the interim analysis is ≥80%, it is recommended to proceed with the planned original sample size of n=268.

If the conditional power at the interim analysis is <80%, it is recommended to proceed with either the planned original sample size, planned maximum sample size, and/or amend eligibility criteria or terminate the study.

9.3 Alpha Level

If required, the nominal alpha level for the final analysis of the primary endpoint will be adjusted using Equation (11) of Mehta and Pocock (2009):

$$2 * (1 - \Phi(\max(z_{\alpha'}(n_2^*)^{-0.5}\left[\frac{(n_2^* - n_1)}{(n_2 - n_1)}(z_{\alpha}\sqrt{n_2} - z_1\sqrt{n_1}) + z_1\sqrt{n_1}\right])))$$

where z_1 is the interim analysis z statistic, z_{α} is 1.645, n_2 is equal to 268 (the planned sample size), n_1 is the sample size used at the unblinded interim analysis, and n_2^* is the final sample size if it is increased. Applying this equation, the only situations where it will be necessary to use a 2-sided significance level lower than 10% is when the sample size is increased and Z_1, the interim standardized normal test statistic associated with the log-transformed relative risk statistic, is less than 1.0956 – corresponding to increasing the sample size when the interim conditional power of statistical significance at a 268 patient analysis is < 44.6%. Refer to Section 9 for additional details regarding the interim analysis.

9.4 Conduct of Unblinded Interim Analysis

This analysis will be conducted by an unblinded programming team which is separate from the blinded statistician and study team, and the unblinded statistician will oversee and review the programming and unblinded results.

The unblinded statistician will confirm the interim analysis details included in the study protocol/SAP/DMC Charter.

The unblinded statistician will minimize the risk of communication of interim results and maintain the integrity of the trial blinding.

The unblinded statistician will proceed with the unblinding of the interim study database according to [redacted] SOP and produce the outputs outlined in the DMC Charter, unless otherwise specified by the Sponsor.

10. BLINDING AND UNBLINDING

10.1 Blinded Statistician

The blinded statistician will define the eligible subjects to be included in each population based on blinded information.

The blinded statistician and blinded programming team will create and validate data sets using dummy randomization data.

The blinded statistician will confirm database lock with the study data manager so that the unblinded final statistical analysis may begin.

10.2 Management of Accidental Unblinding

If a study subject is accidentally unblinded or access to unblinded data is inadvertently granted, the team will follow [redacted] SOP [redacted] which includes notification and assessment of impact with the Sponsor.

10.3 Unblinding for Analysis Purposes at the End of the Trial

Once the confirmation of database lock has occurred, the study statistician will request the production randomization list be exported from RTSM and sent to the blinded statistical team. Upon receipt, the blinded statistical team will be considered unblinded and carryout the final analysis. The statistical team will continue to keep all analysis and unblinding to themselves, only sharing requested information with appropriate members of the sponsor team. The statistical team will not share any unblinded information with anyone until informed to do so by the sponsor.

11. REFERENCES

Gao, P., J. H. Ware and C. Mehta (2008). "Sample size re-estimation for adaptive sequential design in clinical trials." J Biopharm Stat **18**(6): 1184-1196.

Mehta, C. R. and S. J. Pocock (2011). "Adaptive increase in sample size when interim results are promising: A practical guide with examples." Statistics in Medicine **30**(28): 3267-3284.

Khwaja, A. (2012). "KDIGO clinical practice guidelines for acute kidney injury." Nephron Clin Pract **120**(4): c179-184.

12. APPENDICES

12.1 APPENDIX A: SCHEDULE OF ASSESSMENTS

Assessments	Screening	Treatment								Follow-Up			
**Visit Number**	**1** [h]	2 Day of Surgery						**3**	**4** (EOT)	**5** [v]	**6** (discharge)[w]	**7** [x]	**8** (EoS) [x, y]
**Visit Day**	**-30 to -1**	**1**						**2** (24h)	**3** (48h)	**4** (72h)	**7**	**30**	**90**
**Allowed visit window (days)**	**±0**	**±0**						**±0**	**±0**	**±0** [q]	**±2**	**±3**	**±7**
**Visit hour (h)**		**pre**	**0h**	**1h**	**2h**	**6h** [z]	**12h** [z]						
Informed consent	x												
Inclusion/exclusion criteria	x	x											
Medical history	x												
Demographics	x												
Weight and height [a]	x							x	x	x			
Physical examination [b]	x									x			
Pregnancy test (WOCBP only) [c]	x	x											
Hematology lab [d]	x							x	x	x			
Clinical chemistry lab [d]	x							x	x	x			
Liver function lab [d]	x							x	x	x			
Serum creatinine (SCr) [e]	x	x					x	x	x	x	x	x	x
Serum Cystatin C [f]	x	x					x	x	x	x	x	x	x
UACR and UPCR [g]	x	x								x		x	x
Urinalysis		x								x			
Randomization [h]	x												
Record renal replacement therapy								x	x	x		x	x
IMP administration [i]			x [j]			x [k]	x [l]	x [l]	x [l]				
Plasma PK sampling		x		x	x			x, x, x [m]	x, x, x [m]				
12-lead electrocardiogram	x	x						x	x	x			
Vital signs [n]	x	x						x	x	x			

Assessments	Screening	Treatment								Follow-Up			
**Visit Number**	**1** [h]	2 Day of Surgery						**3**	**4** (EOT)	**5** [v]	**6** (discharge)[w]	**7** [x]	**8** (EoS) [x, y]
**Visit Day**	**-30 to -1**	**1**						**2** (24h)	**3** (48h)	**4** (72h)	**7**	**30**	**90**
**Allowed visit window (days)**	**±0**	**±0**						**±0**	**±0**	**±0** [q]	**±2**	**±3**	**±7**
**Visit hour (h)**		**pre**	**0h**	**1h**	**2h**	**6h** [z]	**12h** [z]						
Surgery assessments [o]				←		→							
Discharge from ICU [p]							←				→		
Urine output [q]		←								→			
Urine sampling for biomarkers [r]		x				x		x	x				
Plasma/serum sampling for biomarkers [r]		x						x		x			
Plasma sampling for immunologic biomarkers [s]		x						x, x [s]	x, x [s]				
HRQoL Assessments [t]	x												x
ADA assessment		x										x	x
Concomitant medication recording [u]	←												→
AE recording			←									→	
SAE recording	←												→

Abbreviations: ADA = anti-drug antibody; AE = adverse event; AKI = acute kidney injury; EOS = End of Study; EOT = End-of-Treatment; EQ-5D-5L = European Quality of Life 5 Domain 5-Level Score; HRQoL = health related quality of life; ICU = intensive care unit; IMP = investigational medicinal product; PK = pharmacokinetic; PRO = patient reported outcome; SAE = serious adverse event; SCr = serum creatinine; WOCBP = woman of childbearing potential; UACR = urine albumin to creatinine ratio; UAPR = urine albumin to protein ratio

a. Height only measured at screening (Visit 1). Weight during ICU stay only required if possible.

b. The initial physical examination performed at screening should be comprehensive; all other physical examinations may be abbreviated and symptom driven.

c. A serum pregnancy test completed during the screening period within 48 hours prior to surgery does not need to be repeated on the day of surgery. If the serum pregnancy test occurs more than 48 hours prior to the date of surgery, a serum or urine pregnancy test will also be performed on Day 1 prior to surgery.

d. <u>Hematology Labs:</u> Hematocrit, Hemoglobin (Hb), Mean corpuscular volume (MCV), Mean corpuscular hemoglobin (MCH), Mean corpuscular hemoglobin concentration, Red cell distribution width, Red blood cells, Platelets, Leucocytes (including Neutrophils, Monocytes, Lymphocytes, Eosinophils, Basophils)
<u>Clinical Chemistry Labs:</u> Albumin, Calcium, Chloride, Serum creatinine (SCr), C-reactive protein (CRP), Sodium, estimated glomerular filtration rate (eGFR), Magnesium, Phosphate, Potassium, Blood urea nitrogen (BUN), Uric acid, Glucose
<u>Liver Function Labs:</u> Alanine aminotransferase (ALAT), Alkaline phosphatase (ALP), Aspartate aminotransferase (AST), Bilirubin (total and conjugated), Gamma glutamyltransferase (GGT)

e. The screening sample for SCr must be collected on Day -1 (or day of surgery, see **footnote h**) and will be analyzed locally (to evaluate eligibility and determine correct start dose of RMC-035 according to renal function) and centrally (as baseline for endpoint assessment). All SCr samples collected during hospital stay will be analyzed both locally (to support AKI evaluation) and in a central lab (for the purpose of endpoint assessments). Day 30 and 90 samples will be analyzed centrally.

f. Cystatin C samples will be collected and analyzed in a central lab only for the purpose of endpoint assessments

g. UACR: screening sample will be collected as a spot urine sample and analyzed locally to evaluate albuminuria as an eligibility criterion (in the absence of historical albuminuria data within 3 months prior to randomization). UACR and UPCR: In-hospital samples (Day 1, Visit 2 and Day 4, Visit 5) will be collected either as a First Morning Void (FMV) sample or drawn directly from a Foley catheter and analyzed in a central lab only. Follow-up samples (Day 30, Visit 7 and Day 90, Visit 8) will be collected as FMV samples as possible and analyzed in a central lab only.

h. Randomization must occur on Day -1, ie the day before surgery is intended. All screening assessments may be performed on Day 1 prior to surgery, including randomization. These assessments must be completed prior to any pre-surgical activities, such as administration of fluids or medications, including anesthesia.

i. All 5 doses to be calculated using the same weight measurement that is used for randomization / stratification. IMP will be permanently discontinued in subjects developing AKI stage 2 or higher as per KDIGO guidelines (Appendix 3) or per criteria in Section 5.3.1.

j. IV infusion over 60 minutes, first infusion should start approximately 10 minutes before expected onset of CPB (time point 0 is defined as start of IMP administration)

k. IV infusion over 60 minutes at 6 h (±30 min) after the start of first infusion

l. IV infusion over 30 minutes at 12 h, 24 h and 48 h (±30 min) after the start of first infusion

m. PK sampling at Day 2 and 3 should occur 30 min (±5 min) and 90 min (±15 min) from start of IMP infusion

**Plasma PK Sampling**<br>Study Day	 Time Window	**Predose**<br>≤30 min	**30 min**<br>±5 min	**1 h**<br>±5 min	**90 min**<br>±15 min	**2 h**<br>±15 min
Day 1	Start of Infusion 1 (t=0 h)	x		x		x
Day 2	Start of Infusion 4 (t=24 h)	x	x		x	
Day 3	Start of Infusion 5 (t=48 h)	x	x		x	

n. Vital signs: body temperature, blood pressure, heart rate, respiratory rate, SpO2

o. Data points to collect are type of CPB pump (pulsatile or non-pulsatile, if applicable) and duration of CPB (exact time of initiation and end of CPB), duration of surgery (beginning of surgery defined as exact time of initial skin incision, end of surgery defined as exact time of skin closure), blood loss volume, administration of any fluids during surgery (blood products [red blood cells, plasma, cryoprecipitate, platelets, etc.], crystalloids, colloids, and others), target body temperature during CPB and time at temperature range, duration of cross clamp (minutes), number, position, and graft source bypasses performed, length of time with mean arterial pressure <50 mmHg, valve surgery type (replacement or repair), replacement valve origin (bioprosthetic or mechanical), aortic repair type, and time of admission to the ICU.

p. Time of discharge from ICU to hospital ward, another treatment facility or home

q. Only required as long as Foley catheter is in place

r. Urine samples for evaluation of exploratory urinary (kidney) biomarkers and plasma/serum samples for evaluation of cardiac biomarkers

**Biomarker Sampling**<br>Time Window (in relation to Start of Infusion 1 (t=0 h))	**Predose**<br>≤60 min	**6 h**<br>±30 min	**24 h**<br>±30 min	**48 h**<br>±30 min	**72 h**<br>±30 min
Urine	x	x	x	x	
Plasma/serum	x		x		x

s. Plasma samples for assessment of immunologic biomarkers will be collected at the intervals described below, respectively:

**Immunology Biomarker Sampling**<br>Study Day	 Time Window	**Predose**<br>≤30 min	**90 min**<br>±20 min
Day 1	Start of Infusion 1 (t=0 h)	x	
Day 1	Start of Infusion 2 (t=6 h)		
Day 1	Start of Infusion 3 (t=12 h)		
Day 2	Start of Infusion 4 (t=24 h)	x	x
Day 3	Start of Infusion 5 (t=48 h)	x	x

t. PRO HRQoL assessment: SF 36 and EQ-5D-5L. PRO HRQoL assessments to be performed as early as possible in the screening period.

u. Medications taken within 30 days prior to the day surgery is intended are to be collected. Use of contrast agent within 72 hours prior to the day surgery is intended should be documented as a prior/concomitant medication. When possible, type and quantity of contrast agent should be recorded.

v. Visit 5 must occur at 72 hours from start of first infusion of IMP, with a scheduling window of +/-2 hours.

w. Visit 6 and all associated assessments should occur on the day of hospital discharge. In case subject is discharged on Day 4, discharge (Visit 6) assessments performed prior to discharge on that day are acceptable.

x. Visit may be performed by qualified and trained study staff at the subject's home or other suitable location, where appropriate

y. In case of subject withdrawal, subject should be encouraged to undergo all EOS assessments as an Early Termination visit.

z. Assessments must be performed prior to IMP administration

[redacted]

12.2 APPENDIX B: CKD-EPI CALCULATIONS

The 2009 CKD-EPI calculation for SCr is:

$$eGFR = A * \left(\frac{Scr}{B}\right)^{C} * 0.993^{age} \qquad [if\ African\ American, multiply\ by\ 1.159]$$

Where A, B, and C are:

FEMALE		MALE	
SCr ≤ 0.7	A = 144	SCr ≤ 0.9	A = 141
	B = 0.7		B = 0.9
	C = -0.329		C = -0.411
SCr > 0.7	A = 144	SCr > 0.9	A = 141
	B = 0.7		B = 0.9
	C = -1.209		C = -1.209

The 2012 CKD-EPI calculation for Cystatin C is:

$$eGFR = 133 * \left(\frac{Scys}{0.8}\right)^{A} * 0.996^{age}\ x\ B$$

Where A and B:

	FEMALE	MALE
Scys ≤ 0.8	A = -0.499	A = -0.499
	B = 0.932	B = 1
Scys > 0.8	A = -1.328	A = -1.328
	B = 0.932	B = 1

The 2012 CKD-EPI calculation for SCr and Cystatin C is:

$$eGFR = A * \left(\frac{Scr}{B}\right)^{C} * \left(\frac{Scys}{0.8}\right)^{D} * 0.995^{age} \qquad [if\ African\ American, multiply\ by\ 1.08]$$

Where A, B, C, and D are:

FEMALE	SCr ≤ 0.7	SCr > 0.7
Scys ≤ 0.8	A = 130	A = 130
	B = 0.7	B = 0.7
	C = -0.248	C = -0.601
	D = -0.375	D = -0.375
Scys > 0.8	A = 130	A = 130
	B = 0.7	B = 0.7
	C = -0.248	C = -0.601
	D = -0.711	D = -0.711

MALE	SCr ≤ 0.9	SCr > 0.9
Scys ≤ 0.8	A = 135	A = 135
	B = 0.9	B = 0.9
	C = -0.207	C = -0.601
	D = -0.375	D = -0.375
Scys > 0.8	A = 135	A = 135
	B = 0.9	B = 0.9
	C = -0.207	C = -0.601
	D = -0.711	D = -0.711

12.3 APPENDIX C: DOSING TABLES

12.3.1 Table C1: Volumes of IMP and diluent for dose level 1.3 mg/kg

NOTE! This table is used for dilution of

- **1st** and **2nd** dose for subjects with **eGFR ≥60 mL/min/1.73 m^2**.

Body Weight	Dose (mg)	Volume of IMP (mL)	Volume of 0.9% Sodium Chloride (mL)
**50**	65.0	**10.8**	39.2
**51**	66.3	**11.1**	39.0
**52**	67.6	**11.3**	38.7
**53**	68.9	**11.5**	38.5
**54**	70.2	**11.7**	38.3
**55**	71.5	**11.9**	38.1
**56**	72.8	**12.1**	37.9
**57**	74.1	**12.4**	37.7
**58**	75.4	**12.6**	37.4
**59**	76.7	**12.8**	37.2
**60**	78.0	**13.0**	37.0
**61**	79.3	**13.2**	36.8
**62**	80.6	**13.4**	36.6
**63**	81.9	**13.7**	36.4
**64**	83.2	**13.9**	36.1
**65**	84.5	**14.1**	35.9
**66**	85.8	**14.3**	35.7
**67**	87.1	**14.5**	35.5
**68**	88.4	**14.7**	35.3
**69**	89.7	**15.0**	35.1
**70**	91.0	**15.2**	34.8
**71**	92.3	**15.4**	34.6
**72**	93.6	**15.6**	34.4
**73**	94.9	**15.8**	34.2
**74**	96.2	**16.0**	34.0
**75**	97.5	**16.3**	33.8
**76**	98.8	**16.5**	33.5
**77**	100.1	**16.7**	33.3
**78**	101.4	**16.9**	33.1
**79**	102.7	**17.1**	32.9
**80**	104.0	**17.3**	32.7
**81**	105.3	**17.6**	32.5
**82**	106.6	**17.8**	32.2
**83**	107.9	**18.0**	32.0

**Body Weight**	**Dose (mg)**	**Volume of IMP (mL)**	**Volume of 0.9% Sodium Chloride (mL)**
**84**	109.2	**18.2**	31.8
**85**	110.5	**18.4**	31.6
**86**	111.8	**18.6**	31.4
**87**	113.1	**18.9**	31.2
**88**	114.4	**19.1**	30.9
**89**	115.7	**19.3**	30.7
**90**	117.0	**19.5**	30.5
**91**	118.3	**19.7**	30.3
**92**	119.6	**19.9**	30.1
**93**	120.9	**20.2**	29.9
**94**	122.2	**20.4**	29.6
**95**	123.5	**20.6**	29.4
**96**	124.8	**20.8**	29.2
**97**	126.1	**21.0**	29.0
**98**	127.4	**21.2**	28.8
**99**	128.7	**21.5**	28.6
**100**	130.0	**21.7**	28.3
**101**	131.3	**21.9**	28.1
**102**	132.6	**22.1**	27.9
**103**	133.9	**22.3**	27.7
**104**	135.2	**22.5**	27.5
**105**	136.5	**22.8**	27.3
**106**	137.8	**23.0**	27.0
**107**	139.1	**23.2**	26.8
**108**	140.4	**23.4**	26.6
**109**	141.7	**23.6**	26.4
**110**	143.0	**23.8**	26.2
**111**	144.3	**24.1**	26.0
**112**	145.6	**24.3**	25.7
**113**	146.9	**24.5**	25.5
**114**	148.2	**24.7**	25.3
**115**	149.5	**24.9**	25.1
**116**	150.8	**25.1**	24.9
**117**	152.1	**25.4**	24.7
**118**	153.4	**25.6**	24.4
**119**	154.7	**25.8**	24.2
**120**	156.0	**26.0**	24.0
**121**	157.3	**26.2**	23.8
**122**	158.6	**26.4**	23.6
**123**	159.9	**26.7**	23.4

**Body Weight**	**Dose (mg)**	**Volume of IMP (mL)**	**Volume of 0.9% Sodium Chloride (mL)**
**124**	161.2	**26.9**	23.1
**125**	162.5	**27.1**	22.9
**126**	163.8	**27.3**	22.7
**127**	165.1	**27.5**	22.5
**128**	166.4	**27.7**	22.3
**129**	167.7	**28.0**	22.1
**130**	169.0	**28.2**	21.8
**131**	170.3	**28.4**	21.6
**132**	171.6	**28.6**	21.4
**133**	172.9	**28.8**	21.2
**134**	174.2	**29.0**	21.0
**135**	175.5	**29.3**	20.8
**136**	176.8	**29.5**	20.5
**137**	178.1	**29.7**	20.3
**138**	179.4	**29.9**	20.1
**139**	180.7	**30.1**	19.9
**140**	182.0	**30.3**	19.7
**141**	183.3	**30.6**	19.5
**142**	184.6	**30.8**	19.2
**143**	185.9	**31.0**	19.0
**144**	187.2	**31.2**	18.8
**145**	188.5	**31.4**	18.6
**146**	189.8	**31.6**	18.4
**147**	191.1	**31.9**	18.2
**148**	192.4	**32.1**	17.9
**149**	193.7	**32.3**	17.7
**150**	195.0	**32.5**	17.5
**151**	196.3	**32.7**	17.3
**152**	197.6	**32.9**	17.1
**153**	198.9	**33.2**	16.9
**154**	200.2	**33.4**	16.6
**155**	201.5	**33.6**	16.4
**156**	202.8	**33.8**	16.2
**157**	204.1	**34.0**	16.0

12.3.2 Table C2: Volumes of IMP and diluent for dose level 0.65 mg/kg

Note! This table is used for dilution of

- **all doses** for subjects with **eGFR >30 and <60 mL/min/1.73 m^2** and
- **3rd, 4th**, and **5th** dose for subjects **eGFR ≥60 mL/min/1.73 m^2**

Body Weight	Dose (mg)<br>(for 0.65 mg/kg)	Volume of IMP<br>(mL)	Volume of 0.9% Sodium Chloride (mL)
**50**	32.5	**5.4**	44.6
**51**	33.2	**5.5**	44.5
**52**	33.8	**5.6**	44.4
**53**	34.5	**5.7**	44.3
**54**	35.1	**5.9**	44.2
**55**	35.8	**6.0**	44.0
**56**	36.4	**6.1**	43.9
**57**	37.1	**6.2**	43.8
**58**	37.7	**6.3**	43.7
**59**	38.4	**6.4**	43.6
**60**	39.0	**6.5**	43.5
**61**	39.7	**6.6**	43.4
**62**	40.3	**6.7**	43.3
**63**	41.0	**6.8**	43.2
**64**	41.6	**6.9**	43.1
**65**	42.3	**7.0**	43.0
**66**	42.9	**7.2**	42.9
**67**	43.6	**7.3**	42.7
**68**	44.2	**7.4**	42.6
**69**	44.9	**7.5**	42.5
**70**	45.5	**7.6**	42.4
**71**	46.2	**7.7**	42.3
**72**	46.8	**7.8**	42.2
**73**	47.5	**7.9**	42.1
**74**	48.1	**8.0**	42.0
**75**	48.8	**8.1**	41.9
**76**	49.4	**8.2**	41.8
**77**	50.1	**8.3**	41.7
**78**	50.7	**8.5**	41.6
**79**	51.4	**8.6**	41.4
**80**	52.0	**8.7**	41.3
**81**	52.7	**8.8**	41.2
**82**	53.3	**8.9**	41.1
**83**	54.0	**9.0**	41.0

Body Weight	Dose (mg) (for 0.65 mg/kg)	Volume of IMP (mL)	Volume of 0.9% Sodium Chloride (mL)
**84**	54.6	**9.1**	40.9
**85**	55.3	**9.2**	40.8
**86**	55.9	**9.3**	40.7
**87**	56.6	**9.4**	40.6
**88**	57.2	**9.5**	40.5
**89**	57.9	**9.6**	40.4
**90**	58.5	**9.8**	40.3
**91**	59.2	**9.9**	40.1
**92**	59.8	**10.0**	40.0
**93**	60.5	**10.1**	39.9
**94**	61.1	**10.2**	39.8
**95**	61.8	**10.3**	39.7
**96**	62.4	**10.4**	39.6
**97**	63.1	**10.5**	39.5
**98**	63.7	**10.6**	39.4
**99**	64.4	**10.7**	39.3
**100**	65.0	**10.8**	39.2
**101**	65.7	**10.9**	39.1
**102**	66.3	**11.1**	39.0
**103**	67.0	**11.2**	38.8
**104**	67.6	**11.3**	38.7
**105**	68.3	**11.4**	38.6
**106**	68.9	**11.5**	38.5
**107**	69.6	**11.6**	38.4
**108**	70.2	**11.7**	38.3
**109**	70.9	**11.8**	38.2
**110**	71.5	**11.9**	38.1
**111**	72.2	**12.0**	38.0
**112**	72.8	**12.1**	37.9
**113**	73.5	**12.2**	37.8
**114**	74.1	**12.4**	37.7
**115**	74.8	**12.5**	37.5
**116**	75.4	**12.6**	37.4
**117**	76.1	**12.7**	37.3
**118**	76.7	**12.8**	37.2
**119**	77.4	**12.9**	37.1
**120**	78.0	**13.0**	37.0
**121**	78.7	**13.1**	36.9
**122**	79.3	**13.2**	36.8
**123**	80.0	**13.3**	36.7

**Body Weight**	**Dose (mg) (for 0.65 mg/kg)**	**Volume of IMP (mL)**	**Volume of 0.9% Sodium Chloride (mL)**
**124**	80.6	**13.4**	36.6
**125**	81.3	**13.5**	36.5
**126**	81.9	**13.7**	36.4
**127**	82.6	**13.8**	36.2
**128**	83.2	**13.9**	36.1
**129**	83.9	**14.0**	36.0
**130**	84.5	**14.1**	35.9
**131**	85.2	**14.2**	35.8
**132**	85.8	**14.3**	35.7
**133**	86.5	**14.4**	35.6
**134**	87.1	**14.5**	35.5
**135**	87.8	**14.6**	35.4
**136**	88.4	**14.7**	35.3
**137**	89.1	**14.8**	35.2
**138**	89.7	**15.0**	35.1
**139**	90.4	**15.1**	34.9
**140**	91.0	**15.2**	34.8
**141**	91.7	**15.3**	34.7
**142**	92.3	**15.4**	34.6
**143**	93.0	**15.5**	34.5
**144**	93.6	**15.6**	34.4
**145**	94.3	**15.7**	34.3
**146**	94.9	**15.8**	34.2
**147**	95.6	**15.9**	34.1
**148**	96.2	**16.0**	34.0
**149**	96.9	**16.1**	33.9
**150**	97.5	**16.3**	33.8
**151**	98.2	**16.4**	33.6
**152**	98.8	**16.5**	33.5
**153**	99.5	**16.6**	33.4
**154**	100.1	**16.7**	33.3
**155**	100.8	**16.8**	33.2
**156**	101.4	**16.9**	33.1
**157**	102.1	**17.0**	33.0

12.4 APPENDIX D: eCRF DOCUMENTATION

12.4.1 Table D1: Risk Factors

AKI Risk Number	AKI Risk Description
AKIRISK1	Left ventricular ejection fraction (LVEF) <35% at any time during the 3-month period before or at the time of screening
AKIRISK2	Repeat surgery/history of previous open chest cavity cardiac surgery with or without CPB
AKIRISK3	Confirmed diagnosis of type 2 diabetes (T2DM) at least 3 months prior to screening AND ongoing treatment with an approved anti-diabetic drug
AKIRISK4	Age ≥70 years at the time of screening
AKIRISK5	Heart failure New York Heart Association (NYHA) class II or higher at any time during the 3-month period before or at the time of screening
AKIRISK6	Documented history of previous AKI as per KDIGO criteria longer than 3 months before date of screening
AKIRISK7	Anemia with hemoglobin ≤11 g/dL at any time during the 3-month period before or at the time of screening
AKIRISK8	Albuminuria, defined as urine albumin-to-creatinine ratio (UACR) >100 mg/g in a spot urine sample or > 100 mg/24 hour in a 24-hour urine collection at any time during the 3-month period before or at the time of screening.
AKIRISK9	Estimated glomerular filtration rate is <60 mL/min/1.73 m2 using the CKD-EPI equation with SCr at the time of screening

12.4.2 Table D2: Inclusion/Exclusion Criteria

Inclusion/Exclusion Number	Inclusion/Exclusion Description
Inclusion 1	Institutional Review Board (IRB)-/Independent Ethics Committee (IEC)-approved written Informed Consent and privacy language as per national regulations has been obtained from the subject prior to any study-related procedures
Inclusion 2	Subject has the ability to understand and comply with the study requirements and is able to provide written informed consent
Inclusion 3	Subject age is ≥18 and <85 years

Inclusion 4	Estimated glomerular filtration rate (eGFR) is ≥30 mL/min/1.73 m2 (at screening) using the Chronic Kidney Disease-Epidemiology Collaboration (CKD-EPI) equation with SCr
Inclusion 5a and 5b	Subject is scheduled for non-emergent CABG surgery AND/OR valve surgery (single or multiple valves) AND/OR ascending aorta aneurysm surgery with use of CPB AND AKI risk factors are present (at screening) as specified below:<br>a. If only one type of surgery is scheduled at least two AKI risk factors should be present OR eGFR should be <60 mL/min/1.73m2 (at screening) with or without additional risk factors<br>b. If any combined surgery is scheduled at least one AKI risk factor should be present<br><u>Risk factors for AKI are defined below:</u><br>• Left ventricular ejection fraction (LVEF) <35% at any time during the 3-month period before or at the time of screening as assessed by either echocardiography, cardiac magnetic resonance imaging (MRI) or nuclear scan.<br>• Repeat surgery/history of previous open chest cavity cardiac surgery with or without CPB<br>• Confirmed diagnosis of type 2 diabetes (T2DM) at least 3 months prior to screening AND ongoing treatment with an approved anti-diabetic drug<br>• Age ≥70 years at the time of screening<br>• Heart failure New York Heart Association (NYHA) class II or higher at any time during the 3-month period before or at the time of screening<br>• Documented history of previous AKI as per KDIGO criteria longer than 3 months before date of screening independent of the etiology of AKI<br>• Anemia with hemoglobin ≤11 g/dL at any time during the 3-month period before or at the time of screening<br>• Albuminuria, defined as urine albumin-to-creatinine ratio (UACR) >100 mg/g in a spot urine sample or > 100 mg/24 hour in a 24-hour urine collection at any time during the 3-month period before or at the time of screening.<br>• Estimated glomerular filtration rate is <60 mL/min/1.73 m2 using the CKD-EPI equation with SCr at the time of screening

Inclusion 6a and 6b	Female subject is either:<br>a. Of non-childbearing potential<br>• Postmenopausal (defined as at least 1 year without any menses) prior to screening OR<br>• Documented surgically sterile or status post hysterectomy (at least 1 month prior to screening)<br><br>b. Of childbearing potential<br>• Agree not to try to become pregnant throughout the treatment period, and for 7, days after the final Investigational Medicinal Product (IMP) administration<br>• Must have a negative serum pregnancy test at screening<br>• If sexually active, agree to consistently use a highly effective form of birth control (see Appendix 1 of the study protocol) starting at screening and throughout the treatment period, for 7 days after final IMP administration. If required by local law, 2 highly effective methods of birth control must be used, 1 of which must be a barrier method
Inclusion 7	Female subject must not be breastfeeding starting at screening, throughout the treatment period and for 7 days after the final IMP administration
Inclusion 8	Female subject must not donate ova starting at screening, throughout the treatment period and for 7 days after the final IMP administration
Inclusion 9	Male subject and their female spouse/partner(s) who are of childbearing potential must be using a condom starting at screening and continue to do so throughout the treatment period and for 7 days after final IMP administration. If required by local law, 2 highly effective methods of birth control must be used, 1 of which must be a barrier method.
Inclusion 10	Male subjects must not donate sperm starting from screening, throughout the treatment period for up to 7 days after final IMP administration
Inclusion 11	Subject agrees not to participate in another interventional study from the time of signing the informed consent until the EOS visit
Exclusion 1	Subject has any medical condition that in the opinion of the Investigator makes the subject unsuitable for study participation
Exclusion 2	Subject is scheduled for emergent surgeries (e.g., aortic dissection)

Exclusion 3	Subject is scheduled for CABG and/or valve surgery and/or ascending aorta aneurysm surgery combined with additional non-emergent cardiac surgeries (e.g., congenital heart defects)
Exclusion 4	Subject is scheduled to undergo transcatheter aortic valve implantation (TAVI) or transcatheter aortic valve replacement (TAVR), or off-pump surgeries or left ventricular assist device (LVAD) implantation
Exclusion 5	Subject experiences a cardiogenic shock or hemodynamic instability which require inotropes or vasopressors or other mechanical devices such as intra-aortic balloon counter-pulsation (IABP) within 24 hours prior to surgery
Exclusion 6	Subject has a requirement for any of the following within one week prior to surgery: defibrillator or permanent pacemaker, mechanical ventilation, IABP, LVAD, other forms of mechanical circulatory support (MCS)
Exclusion 7	Subject has been diagnosed with AKI (as defined by KDIGO criteria) within 3 months prior to surgery
Exclusion 8	Required cardiopulmonary resuscitation within 14 days prior to cardiac surgery
Exclusion 9	Ongoing sepsis (as defined by SEPSIS-3, the Third International Consensus Definitions for Sepsis and Septic Shock) within the past 2 weeks or, in the opinion of the Investigator, an untreated diagnosed clinically significant infection (viral or bacterial) prior to or at screening and before randomization.
Exclusion 10	Total bilirubin or alanine aminotransferase (ALT) or aspartate aminotransferase (AST) ≥ 2 times the upper limit of normal (ULN) at screening
Exclusion 11	Subject has a history of solid organ transplantation
Exclusion 12	Subject has a history of renal replacement therapy (RRT)
Exclusion 13	Subject has a medical condition which requires active immunosuppressive treatment
Exclusion 14	Subject has severe allergic asthma defined as confirmed diagnosis of asthma poorly controlled while receiving high-dose inhaled corticosteroid treatment, or with requirement of a high level of treatment to maintain control
Exclusion 15	Subject has an ongoing chemotherapy or radiation therapy for malignancy that may have an impact on kidney function as assessed by the medical monitor
Exclusion 16	Subject has received an investigational medicinal product within the last 90 days (or within 5 half-lives of the investigational drug, whichever is longer)

Exclusion 17	Subject has a known allergy to RMC-035 or one of its constituents, or has previously received RMC-035

12.5 APPENDIX E: DERIVATION OF URINE OUTPUT

Deriving Records for Analysis

- For each subject and urine output record, calculate the start time of the collection and the stop time of collection in minutes compared to the date and time of the first dose of IMP
  - (Start Date/Time – First Dose of IMP Date/Time)/60 = urine start (min)
  - (End Date/Time – First Dose of IMP Date/Time)/60 = urine stop (min)

Subject	Total volume (mL)	Urine Start (min)	Urine Stop (min)	Duration (min)
1001-001	1100	-10	45	55
1001-001	45	44	103	59

- Gaps between urine collection records:
  - ≤30 minute gaps
    - Urine output records with a collection start date/time ≤30 minutes after the collection end date/time of the previous record, then the start date/time of the record will be equal to the end date/time of the previous record. The justification for the transformation is: the accumulated urine in the tubes over the time of replacement of the urine drainage bag will appear in the output of the subsequent sample.

Subject	Total volume (mL)	Urine Start (min)	Urine Stop (min)	Adjusted Urine Start (min)	Adjusted Urine Stop (min)	Adjusted Duration (min)
1001-001	1100	-10	45	-10	45	55
1001-001	45	44	103	45	103	60

  - >30 minute gaps
    - If there is a gap between the start date/time of one record and the end date/time of the previous record, then then a record with a missing volume will be added from the end date/time of the record prior to the gap to the start date/time of the record after the gap.

<u>Data without empty record:</u>

Subject	Total volume (mL)	Urine Start (min)	Urine Stop (min)	Adjusted Urine Start (min)	Adjusted Urine Stop (min)	Adjusted Duration (min)
1001-001	1100	-10	45	-10	45	55
1001-001	45	44	103	45	103	60
1001-001	80	145	205	145	205	60
1001-001	100	206	266	205	266	61

<u>Data WITH empty record:</u>

Subject	Total volume (mL)	Urine Start (min)	Urine Stop (min)	Adjusted Urine Start (min)	Adjusted Urine Stop (min)	Adjusted Duration (min)
1001-001	1100	-10	45	-10	45	55
1001-001	45	44	103	45	103	60
1001-001				103	145	42
1001-001	80	145	205	145	205	60
1001-001	100	206	266	205	266	61

- Now that the urine output records per subject are derived in minutes and gaps between collections are corrected, the urine output records for the analysis of AKI need to be defined:
    - For each record, create a urine volume per minute. Thus, there will be a record for each minute of the adjusted duration (min):

$$Urine\ Volume_{mL\ Per\ 1\ minute} = \frac{Total\ Urine\ Volume\ (mL)}{Adjusted\ Duration\ (min)}$$

For example, the first record for Subject 1001-001 will be transformed to a volume per minute as follows:

Subject	Volume per minute (mL)	Minute of Adjusted Duration
1001-001	20	-10
1001-001	20	-9
1001-001	20	-8
…		
1001-001	20	42
1001-001	20	43
1001-001	20	44
1001-001	20	45

- Please be aware that a missing result and a total volume of 0 mL are NOT the same. A total volume of 0 mL means the subject did not have any urine collected for that time frame.

- The primary endpoint specifies that AKI is defined as >First dose of IMP Date/Time and ≤72 hours (4,320 minutes) after the first dose of IMP. Thus, for each subject, records where the minute is <0 or >4,320 should be removed.

**Subject**	**Volume per minute (mL)**	**Minute of Adjusted Duration**
1001-001	20	1
1001-001	20	2
1001-001	20	3
…		
1001-001	20	42
1001-001	20	43
1001-001	20	44
1001-001	20	45
…		
1001-001	0.75	57
1001-001	0.75	58
1001-001	0.75	59
1001-001	0.75	60

- Now, the records need to transformed back into hours. The urine volume per hour will be summarized by summing the volume per minute using the sequential records in minutes with a maximum of 60 records. For example, the first 60 records per subject will be the first hour of urine collection. For Subject 1001-101, the first hour would be derived as follows:

$$First\ Hour\ Volume_{mL} = (20\ mL\ per\ minute * 45\ minutes) + (0.75\ mL\ per\ minute * 15\ minutes)$$

$$= 911.25\ mL$$

**Subject**	**Hour**	**Total volume (mL)**	**Missing Values**
1001-001	1	911.25	0

- Since there are missing records due to gaps >30 minutes between records, there may not be enough records to calculate the urine volume per hour. Thus, use the following derivations:
    - If >20 minutes (i.e., 20 records) of the urine volume per minute is missing in a 1 hour period, then the total volume per hour will be set to missing.
    - Otherwise, impute the total volume per hour as follows:

$$Urine\ Volume_{mL\ per\ hour} = Urine\ volume_{mL} * \frac{60}{60 - \#\ of\ missing\ records},$$
$$0 \le missing\ values\ \le 20$$

  - **Note: a total volume of 0 mL is NOT considered a missing result.**

- Finally, the urine total volume needs to be adjusted for the subject's weight:

$$Volume_{mL\ per\ kg} = \frac{Total\ Volume_{mL\ per\ hour}}{Weight\ (kg)}$$

The weight of the subject is collected at Screening, Day 2, Day 3, and Day 4. The following weight adjustments will be used for the following intervals:

  - $0<\text{Hours}\leq24$: Adjust using weight at Screening
  - $24<\text{Hours}\leq48$: Adjust using weight at Day 2
  - $48<\text{Hours}\leq72$: Adjust using weight at Day 3

If weight is missing at Screening, then the weight at Day 2 will be used. If weight is missing at any other visit after Screening, then the last non-missing weight will be used.

Derivation of AKI Utilizing DISJOINT Intervals (Utilized in the Primary Analysis):

Review the urine volume per hour (mL) in 6 hour intervals (e.g., hours 1-6, 7-12, 13-18, …, 67-72) for each subject. If within a 6 hour consecutive non-missing interval a subject has all urine volumes per hour <0.5 mL/kg/hour, then the subject will be flagged as having AKI. For AKI stage, repeat this process using 12 and 24 hour consecutive intervals.

Derivation of AKI Utilizing ROLLING Intervals (Utilized in a Sensitivity Analysis):

Review the urine volume per hour (mL) in 6 hour intervals (e.g., hours 1-6, 2-7, 3-8, …, 67-72) for each subject. If within a 6 hour consecutive non-missing interval a subject has all urine volumes per hour <0.5 mL/kg/hour, then the subject will be flagged as having AKI. For AKI stage, repeat this process using 12 and 24 hour consecutive intervals.

12.6 APPENDIX F: CONDITIONAL POWER ANALYSIS

Table F: Sample Size Assessment Utilizing Primary Endpoint (AKI) – mITT Set

	Value
Results used from Cochran-Mantel-Haenszel estimate [1]	
Sample size used at the interim analysis (n1)	x
Estimated RR	x.xx
90 % CI	x.xx,x.xx
Log (RR)	x.xxx
Variance (Log-RR)	xx.xxx
Z1	x.xxx
Computed Values	xx
Conditional power (CP) [2]	

CI, confidence interval; RR, relative risk.
The Modified Intent to Treat Set (mITT) includes all randomized subjects who received at least 1 dose of IMP and is based on randomized treatment group.

[1] Cochran-Mantel-Haenszel estimate with stratification factors of region (North America and Europe) and eGFR at Day -1 (≥60 and <60 mL/min/1.73m^2) (alpha=0.10).

[2] CP = 1 – Phi([zalpha * sqrt(n2) – z1 * sqrt(n1)]/sqrt(tilden2) – (z1 * sqrt(tilden2))/sqrt(n1));
where Phi is the cumulative density function (CDF) for the standard normal N(0,1), alpha is 0.10, zalpha is 1.645, z1 is the z-score corresponding to the Log RR statistic z1=-ln(RR)/sqrt[Var{ln(RR)}], n1 is n of interim analysis, n2 is n of final analysis (268), tilden2 is n2 - n1 (incremental sample size between interim and final analysis).

NOTE: Conditional Power is not provided as a percentage, but on the 0-1 scale.
