# Supplementary material for: Efficacy and safety of therapeutic alpha-1-microglobulin RMC-035 in reducing kidney injury after cardiac surgery: a multicentre, randomised, double-blind, parallel group, phase 2a trial
Source: eClinicalMedicine. 2024 Sep 16;76:102830. doi: 10.1016/j.eclinm.2024.102830 (PMC11421351; doi:10.1016/j.eclinm.2024.102830)
Supplement: List of collaborators [file mmc2.docx]

| **First and middle name** | **Surname** |
| --- | --- |
| Belén | Adrio Nazar |
| Johannes | Böhm |
| Andreas | Böning |
| Craig | Brown |
| Jan | Burkert |
| Benoit | de Varennes |
| Cara | East |
| Dan | Engelman |
| Antonino | Ginel Iglesias |
| Sven | Helms |
| Jay L | Koyner |
| David | Kress |
| Maxime | Laflamme |
| Andre | Lamy |
| Tobias E | Larsson |
| Klaus | Matschke |
| C David | Mazer |
| Guillermo | Muniz Albaiceta |
| Ignacio | Munoz Carvajal |
| Andrej | Myjavec |
| Nicolas | Noiseux |
| Saturo | Osaki |
| Michael | Reusch |
| Guillermo | Reyes Copa |
| Claudio | Ronco |
| Vincent | Scavo |
| Ryan | Shelstad |
| Madhav | Swaminathan |
| Gabor | Szabo |
| Nicholas | Teman |
| Matthias | Thielmann |
| Jan | Vojacek |
| Thorsten | Wahlers |
| Alexander | Zarbock |
